# Insomnia in the Italian Population During Covid-19 Outbreak: A Snapshot on One Major Risk Factor for Depression and Anxiety

**DOI:** 10.3389/fpsyt.2020.579107

**Published:** 2020-12-15

**Authors:** Valeria Bacaro, Marco Chiabudini, Carlo Buonanno, Paola De Bartolo, Dieter Riemann, Francesco Mancini, Chiara Baglioni

**Affiliations:** ^1^Human Sciences Department, University of Rome Guglielmo Marconi, Rome, Italy; ^2^School of Cognitive Psychotherapy, Association of Cognitive Psychology, Rome, Italy; ^3^Lab of Experimental Psychophysiology, IRCCS S. Lucia, Rome, Italy; ^4^Department of Clinical Psychology and Psychophysiology/Sleep, Medicine, Centre for Mental Disorders, University Medical Centre, Freiburg, Germany

**Keywords:** insomnia, sleep, Italian, COVID-19, home confinement, depression, anxiety

## Abstract

**Objectives:** One of the largest clusters of Covid-19 infections was observed in Italy. The population was forced to home confinement, exposing individuals to increased risk for insomnia, which is, in turn, associated with depression and anxiety. Through a cross-sectional online survey targeting all Italian adult population (≥18 yrs), insomnia prevalence and its interactions with relevant factors were investigated.

**Methods:** The survey was distributed from 1st April to 4th May 2020. We collected information on insomnia severity, depression, anxiety, sleep hygiene behaviors, dysfunctional beliefs about sleep, circadian preference, emotion regulation, cognitive flexibility, perceived stress, health habits, self-report of mental disorders, and variables related to individual difference in life changes due to the pandemic's outbreak.

**Results:** The final sample comprised 1,989 persons (38.4 ± 12.8 yrs). Prevalence of clinical insomnia was 18.6%. Results from multivariable linear regression showed that insomnia severity was associated with poor sleep hygiene behaviors [β = 0.11, 95% CI (0.07–0.14)]; dysfunctional beliefs about sleep [β = 0.09, 95% CI (0.08–0.11)]; self-reported mental disorder [β = 2.51, 95% CI (1.8–3.1)]; anxiety [β = 0.33, 95% CI (0.25–0.42)]; and depression [β = 0.24, 95% CI (0.16–0.32)] symptoms.

**Conclusion:** An alarming high prevalence of clinical insomnia was observed. Results suggest that clinical attention should be devoted to problems of insomnia in the Italian population with respect to both prevention and treatment.

## Introduction

On 21st February 2020 several cases of Covid-19 disease emerged in a specific area of Lombardy in Italy. In the following days, the infection spread within the North of the country. On Monday 9th March the entire country was declared “protected area” and in the “lockdown” condition. Particularly, the following rule was established: possibility to go out only for reasons of certified necessity such as for food shopping, work needs, purchase of drugs, or other health reasons. The lockdown was gradually released on 4th of May 2020. This represented one of the largest and most severe clusters of Covid-19 infections worldwide.

This situation exposed most individuals to unprecedented stress, alarming mental health experts for the risk of a major diffusion of psychological disorders within the population (1, 2). Sleep is a fundamental psychophysiological process for brain function and mental health [e.g., (3, 4)]. Home confinement may have played an important role in changing and negatively impacting sleep quality, thus leading to major risk factor for acute insomnia (5). Insomnia, on the other hand, is linked with increased risk for negative health outcomes (6, 7), including depression and anxiety (8, 9). The present paper focuses on insomnia in the Italian population during the lockdown, as a key vulnerability condition for mental health.

Insomnia disorder is defined as difficulties initiating or maintaining sleep, or early morning awakening associated with impaired daytime functioning, which occurs at least 3 nights a week for 3 months [DSM-5, (10)]. Insomnia is associated with reduced quality of life (11–13), increased risk for cardiovascular diseases (14) and for psychopathology, specifically for depression and anxiety (8, 9, 15, 16). Insomnia is considered a key transdiagnostic factor for mental health (3).

A task force of experts in the field (5) pointed out an alarming situation for which during the lockdown condition individuals' sleep quality was potentially challenged by several processes. This could represent a risk factor for the development of acute insomnia which can turn into chronic insomnia and finally exposing individuals to higher risk for psychopathology.

These processes could be summarized as follows:

- Demographic aspects as age and gender are identified as risk factors for insomnia, with women having increased risk compared to men and prevalence rates increasing with age [for a review see (15)].
- Individual differences in personality traits are associated to variability in resilience ability and adaptation to adverse events. Emotion regulation and cognitive flexibility seem to be relevant factors in stress response and adjustment [e.g., (17, 18)]. Gross (19) defines emotion regulation as the process by which people influence emotions they have, when they have them, and how they experience and express them. Particularly, systematic use of expressive suppression [a form of response modulation that consists in inhibiting an ongoing emotion, Gross (20)] has been often conceptualized as a maladaptive response to stressful situations and it is linked with higher risk for psychopathology (21). In turn, systematic use of cognitive reappraisal [a form of response modulation which involves changing the way one thinks about a potentially emotion eliciting event, Gross (20)] is considered a factor of resilience [e.g., (18)]. Cognitive flexibility is defined as the ability to shift between “cognitive sets” or strategies in response to changes in the environment (22). Dispositional use of expressive suppression and lower levels of cognitive flexibility have been associated to poor sleep quality [e.g., (23, 24)].

Furthermore, Stress is the most commonly studied precipitating factor of insomnia (25). Acute stressors, as home confinement and uncertainty regarding future, usually trigger acute insomnia (26).

- Healthy sleep behaviors, such as regular sleep times, use of bed for sleeping only, avoiding using electronic devices in beds; regular physical activity, healthy eating, and alcohol habits, are considered to be protective factors for sleep problems during home confinement (5).
- Individual different levels of knowledge about sleep, consequences of poor sleep, and insomnia may be associated to vulnerability to insomnia (27).

Additionally, specific life conditions could have exposed individuals to increased stress and acute insomnia. For example, de Girolamo et al. (1) identified specific subgroups at risk for mental disorders including those with health related work; individuals tested positive for the virus; relatives of persons who died in this period; individuals with current mental disorders. Further relevant subgroups might be: people with past insomnia and/or mental disorder (28); individuals living alone [(exposed to loneliness, (29)] or living in large families constricted in shared spaces; parents [(who needed to combine family and work responsibilities in home confinement, (5)]; young adults living with parents [exposed to social isolation from peers, (30)]; and individuals with evening chronotype [vulnerable to acute insomnia, (31)]. Finally, as the pandemic spread differently within the country, those in the North may have experienced higher stress compared to people living in the Center and the South of Italy.

**Figure 1:**
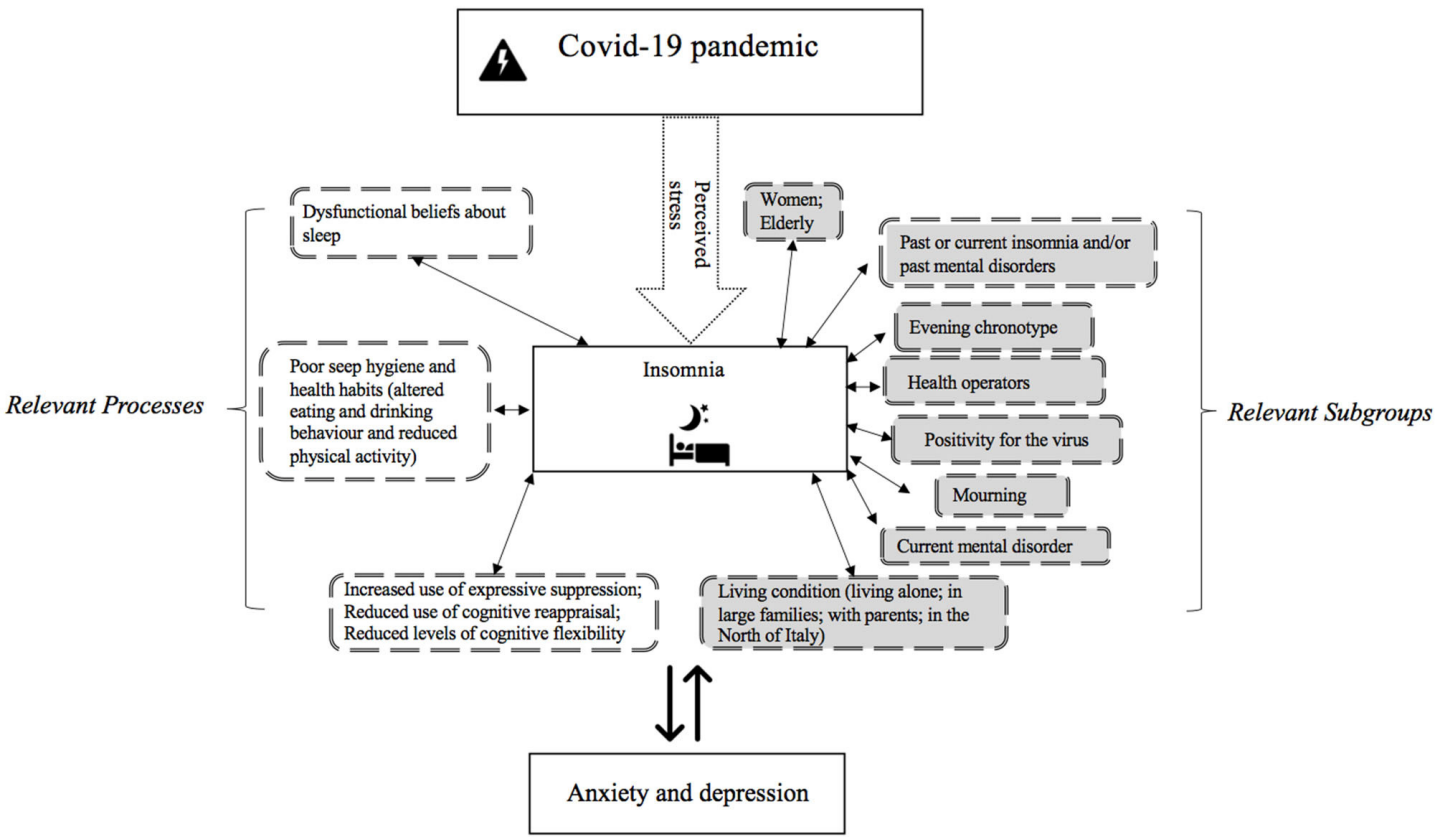
Factors associated with increased insomnia severity during Covid-19 outbreak in Italy. Specifically, the figure highlights relevant population subgroups (in gray) who may experience increased severity of insomnia symptoms during home confinement and relevant processes which may be associated with insomnia disorder severity (in white).

An hypothesis of how all these variables could interact is schematically represented in Figure 1.

The present study aims at providing a snapshot of the prevalence of insomnia disorder in the Italian population during Covid-19 pandemic and investigating its association with potential risk factors and its link with anxiety and depression symptoms.

Specifically, we aimed at evaluating:

1. Prevalence of insomnia disorder considering age and sex.
2. Severity of insomnia in relevant subgroups (in gray in Figure 1).
3. Average scores of relevant processes considering insomnia status (in white in Figure 1).
4. Factors associated with insomnia severity (considering all variables listed in Figure 1).

## Methods

### Procedure and Participants

Recruitment started on the 1st of April, about 3 weeks after the beginning of the lockdown, and was completed on the 4th of May 2020, the day in which the lockdown was gradually released.

The study was conducted in collaboration between the Department of Human Sciences of the University of Study Guglielmo Marconi of Rome (Italy), the Italian School of Specialization in Cognitive Psychotherapy (SPC, Rome, Italy), and the Sleep Laboratory of the Department of Clinical Psychophysiology/Sleep Medicine of the Center for Mental Disorders of the University Medical Center of Freiburg (Germany).

An online survey was created on Survey Monkey platform, an anonymous database and data repository commonly used in research [e.g., (32)]. The completion of the study was voluntary and anonymous and lasted about 15–20 min. First, participants were asked to read accurately the information about the study and to fill in a written informed consent form before starting the survey. Contacts of researchers were given in the informative page for any doubt or need.

The survey was distributed in all Italian territory with different strategies of dissemination: personal contacts of researchers, sponsorship of the University of Study Guglielmo Marconi, sponsorship of the Italian Association of Psychotherapy, the mailing list of the Italian Society of Behavioral and Cognitive Therapy, an article in a major Italian daily newspaper, social networks of the Medical Center Santagostino, sponsorship of the European Cognitive Behavioral Therapy for Insomnia Academy and link on social networks (Facebook, LinkedIn, Instagram).

Inclusion criteria for participation were:

1. To have Italian residence and to spend the lockdown in Italy;
2. To have good knowledge and understanding of the Italian language;
3. To be 18 years old or more.

No compensation for participating in the study was provided. All procedures were performed in accordance with the 1964 Helsinki Declaration and its later amendments, and the study was approved by the Ethical Committee of the Psychological Area of the University of Study Guglielmo Marconi of Rome (Italy).

### Instruments

- An *ad hoc* questionnaire was created to collect the following sociodemographic information:
  - General information: age; region; nationality; weight and height; number of family components; family status [living alone; parent with at least one child <13 yrs; parent(other); individuals living with parent(s); living in couple; or other; occupation (retired; student; homemaker; employee; physician/health operator (i.e., health related work); unemployed].
  - Information on individual difference of the pandemic impact on personal life aspects: change in work satisfaction; change in use of electronic devices; change in physical activity; change in eating habits; change in alcohol drinking habits; worry over the situation; worry to be infected; worry that a loved one gets infected; tested positive to the virus; mourning during home confinement.
  - Information on mental health: past and current insomnia; past and current mental disorder (depression, bipolar disorder, anxiety, panic attack, post-traumatic stress disorder, anorexia, bulimic, obsessive compulsive disorder, post-partum depression, other, and specify).
  - Information on medical health: current medical disorder; current use of medical drugs.

- Insomnia Severity Index [ISI, (33)]
Participants provided answers on a five-point Likert scale, and summing up the results of the respective seven items, ranging from 0 to 28, a total score of insomnia severity during the preceding 2 weeks could be obtained. The total score is interpreted as follows: no insomnia (0–7); subthreshold insomnia (8–14); moderate insomnia (15–21); and severe insomnia (22–28).
- Sleep Hygiene Index [SHI, (34)
The Sleep Hygiene Index is a 13-item self-administered questionnaire which evaluates sleep hygiene behavior, such as regular sleep times, sleep environment, pre-bed routines etc. The items included on the SHI were derived from the diagnostic criteria for inadequate sleep hygiene included in the International Classification of Sleep Disorders (35). Participants were asked to indicate how frequently they engage in specific behaviors (always = 5, frequently = 4, sometimes = 3, rarely = 2, never = 1). Higher scores are indicative of poorer sleep hygiene status. Higher scores indicate less sleep hygiene behaviors. Previous studies used a score below 26 as good sleep hygiene habits, 27–34 as on average, and 35 and above as poor sleep hygiene (34).
- Morningness Eveningness Questionnaire Reduced [MEQr, (36)]
MEQr included five questions: three items requested preferred time for going to bed, getting up and the hour of the day with maximum personal efficiency. Moreover, participants also had to assess the degree of tiredness within the first half an hour after their awakening and to indicate which circadian type they thought they belonged to. The MEQr score was obtained by summing scores of each question and ranged from 4 to 25. Scores above 18 identified subjects as morning types and scores below 11 as evening types.
- Emotion Regulation Questionnaire [ERQ, (37)]
The ERQ is a 10-item self-report scale assessing two individual strategies that people adopt in order to regulate their emotions: cognitive reappraisal and expressive suppression. Participants rate how much they agree with self-descriptive statements reflecting cognitive reappraisal or expressive suppression on a 7-point Likert-type scale. Higher ERQ scores indicate more frequent use of that strategy.
- Perceived Stress Scale [PSS, (38)]
The PSS measures the perception of stress by asking respondents to rate the frequency of their thoughts and feelings related to situations occurred in the last month. It consists of 10 items rated on a five-point Likert-type scale, ranging from “Never,” coded 0, to “Very Often,” coded 4. Scores of from 1 to 10 indicates low levels of stress; 11 to 14 are average scores; scoring 15–18 are medium to high levels and 19 and above indicates high levels of perceived stress.
- Hospital Anxiety and Depression Scale [HADS, (39)]
The HADS consists of seven items rating anxiety and seven items rating depression. Each item is scored from 0 to 3. Anxiety and depression values are the sums of the corresponding item scores. Patients can be subsequently allocated to one of the three following categories for anxiety and depression, based on the individual sum scores: non-case (0–7), borderline case (8–10), and clinical case (11 and above).
- Dysfunctional Beliefs and Attitudes about Sleep [DBAS-16, (40)]
The DBAS-16 was administered to evaluate beliefs and attitudes about sleep in this study. The scoring range for each item was from 1 (at the “strongly disagree” pole) to 5 (at the “strongly agree” pole). Lower scores represent more dysfunctional beliefs.
- Cognitive Flexibility Inventory [CFI, (41)]
The CFI 20-item self-report measure is designed to assess the levels of CF evidenced by individuals engaged in cognitive behavioral thought challenging interventions. The CFI items consist of statements dealing with beliefs and feelings about behavior for which individuals could indicate their agreement or disagreement. The CFI comprises two subscales, the Alternatives and Control subscale that measure three aspects of cognitive flexibility: (a) the tendency to perceive difficult situations as controllable; (b) the ability to perceive multiple alternative explanations for life occurrences and human behavior; and (c) the ability to generate multiple alternative solutions to difficult situations. Each item is scored on a seven-point Likert scale, ranging from strongly disagree (1) to strongly agree (7). The scoring procedures specified for the CFI require reverse scoring of select items and then summing the numerical response values to obtain a total score. Higher scores on both scales are indicative of greater cognitive adaptability associated with greater CF when encountering stressful situations; lower scores are indicative of greater cognitive rigidity associated with less cognitive adaptability when encountering stressful situations.

### Statistical Analysis

Data analyses were conducted from May 4th 2020, date in which the recruitment was concluded. Data analyses were discussed with and supervised by a professional biostatistician (MC) and were performed with R (Version 3.5.1.).

Two analysis populations were defined, the enrolled set which comprised all participants who fulfilled the inclusion criteria, and the main analysis set which included all participants of the enrolled set for whom all questionnaires were evaluable. Descriptive analysis of patient characteristics did not reveal any substantial differences between the two analysis populations (see Supplementary Document 1). All further analysis were conducted on the main analysis set. Results for descriptive statistics were expressed in means ± standard deviations for continuous variables and in absolute and relative frequencies for categorical variables. Stratified analyses allow to assess effects in specific strata thus reducing bias. A linear multivariable regression model was used to determine the association between several independent variables considered as predictors and severity of insomnia as outcome. Predictors included sex, age, past self-reported insomnia, past mental disorder, current self-reported insomnia, current self-reported mental disorder, expressive suppression (ES), cognitive reappraisal (CR), cognitive flexibility, perceived stress, sleep hygiene behaviors, dysfunctional beliefs about sleep, exercise behavior, eating behavior, drinking behavior, use of electronic devices, region of residence, health related work, tested positive to the virus, mourning, number of household members, circadian preference, anxiety, and depression. A detailed list of all variables and their description is reported in Supplementary Document 2. The Bonferroni-Holm method (42) was used to adjust for multiple testing. Multicollinearity among factors was examined by analyzing the (generalized) variance inflation factors with a cut-off of 5 (43).

## Results

### Sample Characteristics

A total of 2,652 individuals gave consent and answered the survey. 1989 completed all validated questionnaires, and were used for the main analyses.

In Table 1 the main sample characteristics are presented. All descriptive data are available in Supplementary Document 1. One thousand, five hundred fifteen were females (37.5 ± 12.42 yrs) and 474 males (41.4 ± 13.8 yrs). The mean age of participants was 38.4 ± 12.8 yrs. (age range: 18–90). 39.7% of the participants came from the north of Italy, 35.6% from the center, 17.7% from the south, and 6.1% from the islands. 27.4% lived home confinement situation in the same home with parents, 27% with partner, and 13.2% alone.

**Table 1:** Sample characteristics.

**Variables**		**Overall**	**Females**	**Males**
*N*		1,989	1,515	474
**General information**				
Age (Mean ± SD)		38.4 ± 12.88	37.5 ± 12.42	41.4 ± 13.85
Region *n* (%)	North	790 (39.7%)	614 (40.5%)	176 (37.1%)
	Center	709 (35.6%)	517 (34.1%)	192 (40.5%)
	South	353 (17.7%)	278 (18.3%)	75 (15.8%)
	Islands	122 (6.1%)	96 (6.3%)	26 (5.5%)
	Unknown	15 (0.8%)	10 (0.7%)	5 (1.1%)
Family status *n* (%)	Alone	263 (13.2%)	190 (12.5%)	73 (15.4%)
	With partner	537 (27.0%)	415 (27.4%)	122 (25.7%)
	With at least one child below 13 years	358 (18.0%)	276 (18.2%)	82 (17.3%)
	With other children	203 (10.2%)	149 (9.8%)	54 (11.4%)
	With parents	545 (27.4%)	430 (28.4%)	115 (24.3%)
	Other	83 (4.2%)	55 (3.6%)	28 (5.9%)
Occupation *n* (%)	Student	269 (13.5%)	228 (15.0%)	41 (8.6%)
	Employed	1,174 (59.0%)	863 (57.0%)	311 (65.6%)
	Health related work	103 (5.2%)	87 (5.7%)	16 (3.4%)
	Homemaker	32 (1.6%)	31 (2.0%)	1 (0.2%)
	Unemployed	96 (4.8%)	81 (5.3%)	15 (3.2%)
	Retired	61 (3.1%)	35 (2.3%)	26 (5.5%)
	Other	250 (12.6%)	187 (12.3%)	63 (13.3%)
	Missing	4 (0.2%)	3 (0.2%)	1 (0.2%)
Number of household members	0	263 (13.2%)	190 (12.5%)	73 (15.4%)
	1	687 (34.5%)	533 (35.2%)	154 (32.5%)
	2	496 (24.9%)	384 (25.3%)	112 (23.6%)
	3	421 (21.2%)	312 (20.6%)	109 (23.0%)
	4	98 (4.9%)	76 (5.0%)	22 (4.6%)
	5	21 (1.1%)	17 (1.1%)	4 (0.8%)
	6	2 (0.1%)	2 (0.1%)	0 (0.0%)
	7	1 (0.1%)	1 (0.1%)	0 (0.0%)
**Pandemic impact**				
Tested positive *n* (%)	Yes	26 (1.3%)	23 (1.5%)	3 (0.6%)
	No	1,955 (98.3%)	1,485 (98.0%)	470 (99.2%)
	Missing	8 (0.4%)	7 (0.5%)	1 (0.2%)
Increased use of devices *n* (%)	Yes	1,640 (82.5%)	1,290 (85.1%)	350 (73.8%)
	No	334 (16.8%)	213 (14.1%)	121(25.5%)
	Missing	15 (0.8%)	12 (0.8%)	3 (0.6%)
Eating habits *n* (%)	Yes, I eat more	778 (39.1%)	597 (39.4%)	181 (38.2%)
	Yes, I eat less healthy	227 (11.4%)	189 (12.5%)	38 (8.0%)
	Yes, I eat less	158 (7.9%)	118 (7.8%)	40 (8.4%)
	Yes, I eat more healthy	261 (13.1%)	194 (12.8%)	67 (14.1%)
	No, not changed	559 (28.1%)	413 (27.3%)	146 (30.8%)
Change in work satisfaction (Mean ± SD)		−14.7 ± 24.52	−15.2 ± 24.83	−13.2 ± 23.56
**Mental health**				
Current insomnia *n* (%)	No	1,792 (90.1%)	1,351 (89.2%)	441 (93.0%)
	Yes	197 (9.9%)	164 (10.8%)	33 (7.0%)
Past insomnia *n* (%)	Yes	276 (13.9%)	228 (15.0%)	48 (10.1%)
	No	1,713 (86.1%)	1,287 (85.0%)	426 (89.9%)
Current mental disorders (other than insomnia)	Yes	299 (15.0%)	253 (16.7%)	46 (9.7%)
	No	1,690 (85.0%)	1,262 (83.3%)	428 (90.3%)
Past mental disorders (other than insomnia)	*Yes*	577 (29.0%)	480 (31.7%)	97 (20.5%)
	*No*	1,412 (71.0%)	1,035 (68.3%)	377 (79.5%)
**Medical health**				
Current disorders *n* (%)	*Yes*	299 (15.0%)	253 (16.7%)	46 (9.7%)
	*No*	1,690 (85.0%)	1,262 (83.3%)	428 (90.3%)

*Region: North was composed of Lombardia, Piemonte, Veneto, Liguria, Emilia Romagna, Valle D'Aosta, Trentino Alto Adige, Friuli Venezia Giulia; Center was composed of Lazio, Toscana, Marche, Umbria; South was composed of Abbruzzo, Molise, Campania, Basilicata, Puglia, Calabria; Islands: Sicily, Sardinia; Current/past mental disorder: insomnia, depression, bipolar disorder, anxiety, panic attack, post-traumatic stress disorder, anorexia, bulimic, obsessive compulsive disorder, post-partum depression, other, and specify; change in work satisfaction was calculated as current satisfaction in a scale from 0 to 100 minus past satisfaction in a scale from 0 to 100*.

Fifty-nine percentage of participants reported to be employed and 5% to have a health related work. 13.7% reported to be students, 5.2% unemployed, 1.8% homemaker, and 3.2% retired. For working participants, a severe average decrease in work satisfaction compared to before home confinement (calculated as current satisfaction in a scale from 0 to 100 minus past satisfaction in a scale from 0 to 100) was observed (−14.7 ± 24.5).

13.9% reported suffering in the past from insomnia disorder and 29% of other mental disorder. 9.9% of participants reported to suffer of current insomnia, 15% reported current mental disorder, and 15% reported current medical disorder. 69.7% of participants did not regularly assume drugs.

Most participants reported increased use of electronic devices during home confinement (82.5%); 49.7% continued to do exercise also in quarantine and 39.1% reported to eat more during this period. Among all respondents, 1.3% was infected with Covid-19 and 6.7% lost a loved one during the isolation period (mourning). Overall worry over Covid-19 was high (in a range from 1 to 100: overall: 68 ± 23.22, F: 69.9 ± 22.23; M: 61.6 ± 25.16). Worry to be infected (in a range from 0 to 100: overall: 42.5 ± 27.07, F: 43.6 ± 27.09; M: 39 ± 26.7) was lower with respect to worry that a loved one could be infected (in a range from 1 to 100: overall: 68.0 ± 28.13; F: 70.0 ± 27.75; M: 61.7 ± 28.41).

In Table 2 average scores of participants to the questionnaire are provided. Specifically, overall, high average of insomnia severity (8.4 ± 6.20; F: 8.6 ± 6.1; M: 7.7 ± 6.2) was observed. Males of our sample reported slightly less dysfunctional beliefs about sleep (31.1 ± 18.56) compared to females (34.5 ± 18.05). Regarding circadian preferences, 19.6% of our sample reported morning, 66.8% intermediate and 13.6% evening chronotype. Perceived stress was scored as lower in males (16.9 ± 7.19) compared to females (20.6 ± 7.15). Participants reported an average anxiety score of 7.9 ± 3.89 and an average depression score of 6.0 ± 3.72 (in both cases scores were higher in females than males). Overall, participants used more cognitive reappraisal strategy (28.9 ± 7.25) than emotion suppression strategy (13.2 ± 5.42). Finally, participants reported a mean cognitive flexibility score of 104.5 ± 17.17.

**Table 2:** Participants scores at questionnaires.

**Questionnaires**		**Total**	**Females**	**Males**
ISI total score	Mean ± StD	8.4 ± 6.20	8.6 ± 6.18	7.7 ± 6.23
SHI total score	Mean ± StD	12.8 ± 7.32	12.9 ± 7.37	12.4 ± 7.15
DBAS-16 total score	Mean ± StD	33.7 ± 18.23	34.5 ± 18.05	31.1 ± 18.56
MEQ categories	Morning type *n* (%)	390 (19.6%)	285 (18.8%)	105 (22.2%)
	Intermediate type *n* (%)	1,329 (66.8%)	1,025 (67.7%)	304 (64.1%)
	Evening type *n* (%)	270 (13.6%)	205 (13.5%)	65 (13.7%)
ERQ-CR total score	Mean ± StD	28.9 ± 7.25	29.2 ± 7.23	28.1 ± 7.27
ERQ-ES total score	Mean ± StD	13.2 ± 5.42	12.6 ± 5.39	14.9 ± 5.15
CFI total score	Mean ± StD	104.5 ± 17.17	103.6 ± 17.11	107.2 ± 17.09
PSS total score	Mean ± StD	19.7 ± 7.33	20.6 ± 7.15	16.9 ± 7.19
HADS anxiety total score	Mean ± StD	7.9 ± 3.89	8.3 ± 3.86	6.6 ± 3.72
HADS depression total score	Mean ± StD	6.0 ± 3.72	6.2 ± 3.72	5.5 ± 3.70

*ISI, Insomnia Severity Index; SHI, Sleep Hygiene Index; DBAS, Dysfunctional Beliefs and Attidudes about Sleep; MEQ, Morningness Eveningness Questionnaire; ERQ-CR, Emotion Regulation Questionnaire-Cognitive Reappraisal; ERQ-ES, Emotion Regulation Questionnaire-Emotion Suppression; CFI, Cognitive Flexibility Inventory; PSS, Perceived Stress Scale; HADS, Hospitalized Anxiety and Depression scale*.

### Prevalence of Insomnia

Considering insomnia severity (ISI), 49.1% presented absence of insomnia; 32.3% presented subthreshold insomnia, 18.6% reported clinical insomnia (15.8% symptoms for moderate insomnia, and 2.8% symptoms of severe insomnia). As showed in Table 3, females reported higher prevalence of clinical insomnia (moderate insomnia + severe insomnia = 19.0%) compared to males (17.3%). High prevalence of insomnia was observed in the 18–30 age group, with extreme rates for males (22.9% moderate insomnia +severe insomnia, F: 21.5%; M: 28.3%).

**Table 3:** Prevalence of insomnia.

	**Overall**				**Female**				**Male**			
	**Age 18–30 (*n* = 666)**	**Age 31–65 (*n* = 1,258)**	**Age > 65 (*n* = 65)**	**All ages (*n* = 1,989)**	**Age 18–30 (*n* = 539)**	**Age 31–65 (*n* = 937)**	**Age >65 (*n* = 39)**	**All ages (n = 1,515)**	**Age 18–30 (*n*=127)**	**Age 31–65 (*n* = 321)**	**Age >65 (*n* = 26)**	**All ages (*n* = 474)**
Absence of insomnia *n* (%)	280 (42.0)	654 (52.0)	43 (66.2)	977 (49.1)	231 (42.9)	457 (48.8)	24 (61.5)	712 (47.0)	49 (38.6)	197 (61.4)	19 (73.1)	265 (55.9)
Subthreshold insomnia *n* (%)	234 (35.1)	394 (31.3)	14 (21.5)	642 (32.3)	192 (35.6)	312 (33.3)	11 (28.2)	515 (34.0)	42 (33.1)	82 (25.5)	3 (11.5)	127 (26.8)
Moderate insomnia *n* (%)	129 (19.4)	177 (14.1)	8 (12.3)	314 (15.8)	98 (18.2)	140 (14.9)	4 (10.3)	242 (16.0)	31 (24.4)	37 (11.5)	4 (15.4)	72 (15.2)
Severe insomnia *n* (%)	23 (3.5)	33 (2.6)	0 (0.0)	56 (2.8)	18 (3.3)	28 (3.0)	0 (0.0)	46 (3.0)	5 (3.9)	5 (1.6)	0 (0.0)	10 (2.1)

### Severity of Insomnia in Relevant Subgroups

Table 4 shows insomnia severity means in relevant subgroups (indicated in the gray boxes in Figure 1). Subgroups showing more severe insomnia were people who reported current presence of insomnia (13.8 ± 4.96) or other mental disorder (13.5 ± 6.03); individuals living with parents (9.5 ± 6.42); participants aged 18–30 (9.3 ± 6.17), people with evening chronotype (10.8 ± 6.42), participants in mourning (9.2 ± 6.22), those living alone (9.0 ± 6.23), and those living in 5 or more in the same house (9.1 ± 6.36).

**Table 4:** Insomnia severity in relevant population subgroups.

		***n***	**Mean ± StD**
**Overall**		1,989	8.4 ± 6.20
Sex	Female	1,515	8.6 ± 6.18
	Male	474	7.7 ± 6.23
Age	18–30	666	9.3 ± 6.23
	31–65	1,258	8.0 ± 6.17
	>65	65	6.3 ± 5.43
Health related work	Yes	103	8.4 ± 5.81
	No	1,882	8.4 ± 6.23
Tested positive	Yes	26	8.1 ± 6.31
	No	1,955	8.4 ± 6.21
Mourning	Yes	133	9.2 ± 6.22
	No	1,850	8.4 ± 6.20
Current mental disorder	Any mental disorder (± insomnia)	299	13.5 ± 6.03
	Insomnia only	31	13.8 ± 4.96
	No mental disorder	1,659	7.4 ± 5.73
Family status	Alone	263	8.9 ± 6.72
	With partner	537	7.6 ± 5.79
	With at least one child below 13	358	7.7 ± 6.08
	With other children	203	8.1 ± 5.58
	With parent(s)	545	9.5 ± 6.42
	Other	83	9.3 ± 6.56
Number of household members	1	263	8.9 ± 6.72
	2	687	7.9 ± 6.02
	3–4	917	8.6 ± 6.15
	≥5	122	9.1 ± 6.36
Circadian preference	Morning type	390	7.5 ± 6.29
	Intermediate type	1,329	8.2 ± 6.05
	Evening type	270	10.8 ± 6.27
Region	North	790	8.4 ± 6.29
	Other	1,199	8.4 ± 6.15

### Differences in Relevant Processes by Insomnia Status

Table 5 shows mean levels of relevant processes (indicated in the white boxes in Figure 1) by insomnia status. Emotional suppression, cognitive reappraisal, cognitive flexibility, perceived stress, sleep hygiene behaviors, dysfunctional beliefs about sleep, anxiety, and depression were evaluated in individuals with different insomnia status, based on the ISI questionnaire. Results showed clear association of insomnia severity with reduced levels of sleep hygiene and cognitive flexibility, and, increased levels of stress, anxiety, depression, and dysfunctional beliefs about sleep.

**Table 5:** Differences in relevant processes by insomnia severity subgroups.

**Variables**	**Absence of insomnia**	**Subthreshold insomnia**	**Moderate insomnia**	**Severe insomnia**
SHI (Mean ± StD)	10.3 ± 6.07	14.0 ± 7.30	17.0 ± 7.11	20.9 ± 8.72
ERQ-CR (Mean ± StD)	29.7 ± 7.12	28.8 ± 7.05	27.4 ± 7.29	25.8 ± 9.28
ERQ-ES (Mean ± StD)	12.5 ± 5.29	13.4 ± 5.40	14.4 ± 5.57	14.0 ± 5.75
PSS (Mean ± StD)	16.6 ± 6.56	21.2 ± 6.59	24.6 ± 6.45	27.8 ± 6.04
HADS anxiety (Mean ± StD)	6.1 ± 3.13	8.5 ± 3.46	10.9 ± 3.57	14.1 ± 3.56
HADS depression (Mean ± StD)	4.4 ± 3.13	6.6 ± 3.26	8.8 ± 3.55	10.5 ± 4.01
DBAS-16 (Mean ± StD)	25.6 ± 14.61	36.6 ± 15.97	47.5 ± 16.81	64.1 ± 18.05
CFI (Mean ± StD)	107.6 ± 15.70	102.9 ± 17.34	99.5 ± 18.08	95.4 ± 22.38

*ISI, Insomnia Severity Index; SHI, Sleep Hygiene Index; ERQ, Emotion Regulation Questionnaire; CR, Cognitive reappraisal; ES, expressive suppression; PSS, Perceived Stress; HADS, Hospital Anxiety and Depression Scale; DBAS, Dysfunctional Beliefs and Attitudes about Sleep; CFI, Cognitive Flexibility Inventory*.

### Identification of Factors Associated With Insomnia

**Figure 2:**
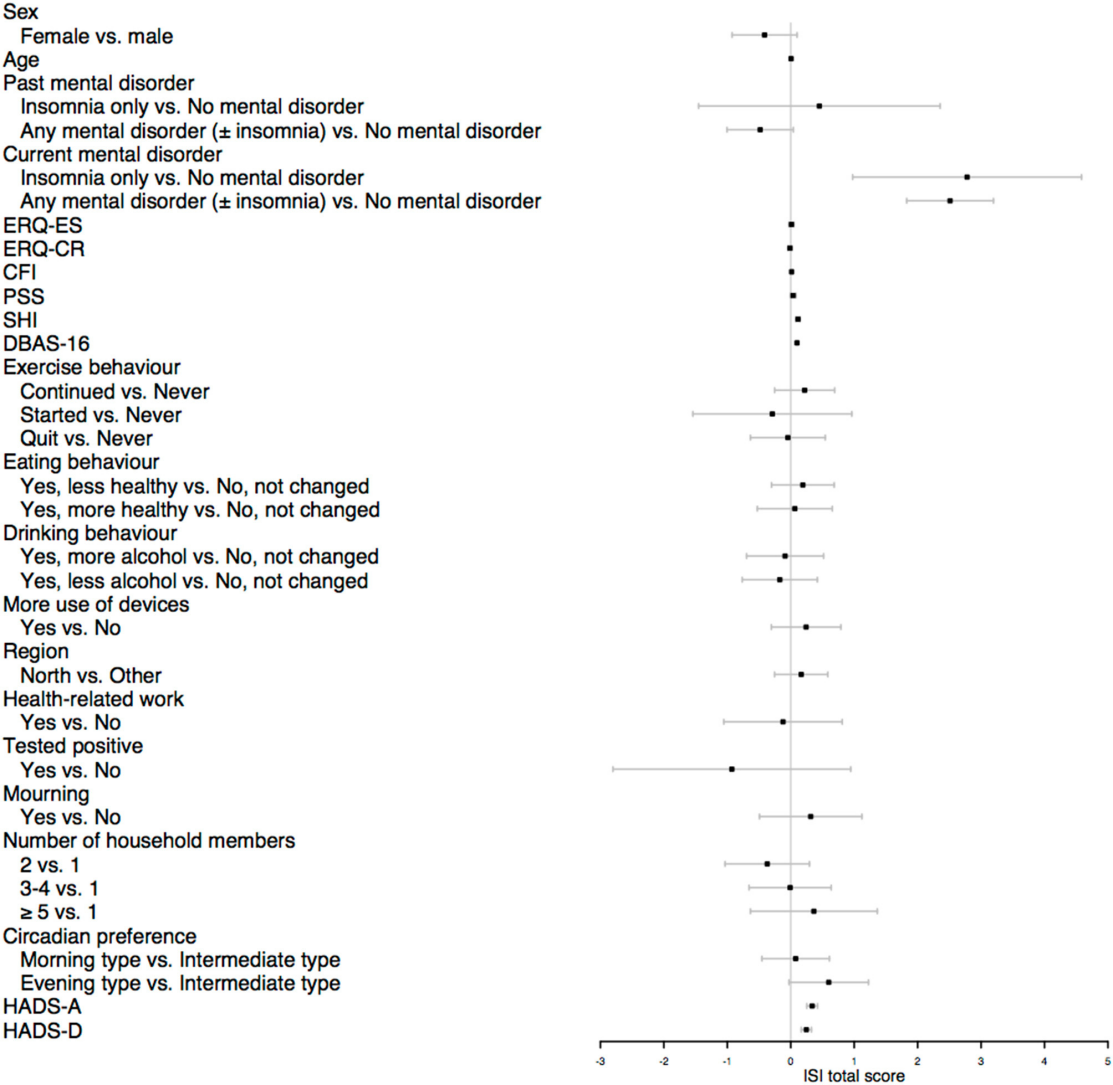
Forest plot of multivariable linear regression. Results were plotted as unstandardized beta coefficients with 95% Confidence Intervals. ERQ, Emotion Regulation Questionnaire; ES, expressive suppression; CR, cognitive reappraisal; CFI, Cognitive Flexibility Inventory; PSS, Perceived Stress Scale; SHI, Sleep Hygiene Index; DBAS-16, Dysfunctional Beliefs and Attitudes about Sleep; HADS, Hospital Anxiety and Depression Scale; HADS-A, anxiety symptoms; HADS-D, depressive symptoms.

Results of linear multivariable regression model are presented in Figure 2 while specific results for all investigated covariates are available in Supplementary Document 2. No substantial changes in the results' interpretation were found after adjusting for multiple testing. No indication for multicollinearity among these variables in the analysis (GVIF < 5) was observed. Large effects were detected for self- report of current insomnia [β = 2.77, 95% (0.97; 4.58), *p* < 0.005] and self-report of current mental disorder [β = 2.51, 95% CI (1.82; 3.19), *p* < 0.001]. Furthermore, results showed that poor sleep hygiene behaviors [β = 0.11, 95% CI (0.07; 0.14), *p* < 0.001] and dysfunctional beliefs about sleep [β = 0.09, 95% CI (0.08; 0.11), *p* < 0.001] are significantly associated to insomnia severity. Finally, results highlighted a significant role of anxiety [β = 0.33, 95% CI (0.25; 0.42), *p* < 0.001] and depression [β = 0.24, 95% CI (0.16; 0.32), *p* < 0.001] symptoms as predictors of insomnia.

## Discussion and Clinical Implications

The present study aimed at determining the prevalence of insomnia disorder and its association with several relevant factors in the Italian adult population during Covid-19 outbreak. Results showed an alarming high prevalence of clinical insomnia of 18.6%, compared to normative data from Europe of 10.1% and from Italy of 7.0% (44, 45). Future studies, in Italy and in other countries, should investigate further the effects of the pandemic's outbreak on insomnia comparing data during and after the pandemic in order to promote cross-cultural comparisons. Specifically, wide-ranging online investigations, using validated questionnaires in different languages, could evaluate the trend of psychological variables with the progress of the pandemic up to the post-pandemic time.

Higher prevalence rates were observed in young males (18–30 yrs). Consistently, high means of insomnia severity were found in those living with parents, mainly overlapping with the young adults group. Some aspects of home confinement, e.g., deprivation from physical social life with peers, increase of virtual social life and time with parents at home, may have challenged mental health particularly in younger individuals (and our results suggest in males more than in females). Instead, participants >65 yrs. did not show high levels of insomnia severity and resulted to be averagely in good health. This may be explained by the fact that only those with high functioning and technological skills answered to our survey. Higher prevalence of insomnia severity was observed in people in mourning and in individuals reporting mental disorders, consistently with previous hypotheses [e.g., (1)].

A clear association was evidenced between insomnia severity and poor sleep hygiene behaviors, dysfunctional beliefs about sleep, anxiety, depression, perceived stress, and cognitive flexibility. Sleep, insomnia, stress, emotion and behavior regulation, and cognitive flexibility represent transdiagnostic processes in mental health. Recent findings showed that Cognitive and Behavior Therapy (CBT) may be strongly supported by empirical findings and respond adequately to individual complex conditions [e.g., (46)]. In the pandemic context, targeting transdiagnostic processes to increase resilient, and adjustment responses seem particularly promising.

Results from linear regression model showed that factors most associated with insomnia severity are: psychopathology (i.e., current present of mental disorder, higher means in measures of depression and anxiety symptoms), and behaviors and cognitions associated to sleep (i.e., poor sleep hygiene behaviors; and dysfunctional beliefs about sleep). Sex and age were not identified as associated *per se* with insomnia severity, suggesting that they could be confounders.

Insomnia is commonly associated to depression, anxiety, and mental disorders in general (47), and cumulative evidence highlighted its transdiagnostic nature in psychopathology [e.g., (3)]. Insomnia disorder (with or without comorbidities) responds to its specific treatment [Cognitive Behavior Treatment for Insomnia, CBT-I, (15, 44)]. Instead, it often persists after successful treatment of other conditions, such as depression (48). Based on this literature, CBT-I could be successfully offered to all patients with mental disorders presenting insomnia, as indicated in preliminary clinical research. Specifically, recent data showed that treating insomnia by offering CBT-I to patients with mental disorders could have important benefits [e.g., (49)]. Nevertheless, the standard CBT-I protocol may need to be adapted depending on patients specific symptomatology. For example, patients with bipolar disorder may not benefit by sleep restriction, which is a key behavioral strategy in the CBT-I protocol, consisting in limiting the amount of time you allow yourself to sleep in the bed, because of its potential role in triggering manic episodes (50). Harvey et al. (50) conducted a randomized controlled trial comparing 30 bipolar patients who received bipolar disorder–specific modification of CBT-I and 28 patients who received psychoeducation therapy. The authors found that the experimental group experienced significantly lower hypomania/mania relapse rate, reduced insomnia severity, and higher rates of insomnia remission at posttreatment compared to the psychoeducation control group.

Furthermore, post-traumatic stress disorder is often associated with insomnia, characterized by hypervigilance at night and fear of going to bed or to sleep (51). Thus, much of the studies investigating insomnia in this population had included in the CBT-I a specific intervention targeting nightmares. Specifically, imagery rehearsal therapy, a form of cognitive therapy targeting nightmares consisting in recalling the nightmare, changing any part of the dream to a more positive one, and rehearsing the rewritten dream scenario, is recommended for treatment of post-traumatic-related nightmares (52). Further clinical research is needed to deepen the efficacy of CBT-I and adapted protocols for insomnia in comorbidities with different psychopathologies, and also to better understand the promising role of CBT-I in preventing mental disorders.

Since the alarming presence of high prevalence of clinical insomnia which was observed in correspondence of Covid-19 outbreak, implementing CBT-I in the mental health services and in primary care could be very useful to prevent the development of psychopathologies.

Despite the cross-sectional nature of this study, results suggest an important preventive role of sleep hygiene behaviors and adequate knowledge about sleep in the development of a clinically relevant disorder. Since, the role of sleep hygiene knowledge was already showed in previous literature, high quality clinical programs in the post-pandemic scenario, targeting sleep and insomnia could be implemented in primary care [for a review see (44)]. This could be especially important in younger adults, in order to investigate their health preventive and promotion role. This seems to be specifically important in this second phase of the pandemic, in which mental health operators should make all efforts to contain and repair the damages which long home confinement has provoked. Studies could further investigate the longitudinal course of sleep patterns and behaviors in the different phases of the pandemic and in the post-pandemic context. Furthermore, future longitudinal studies could explore further the association between sleep hygiene behaviors and insomnia in the general population and in mental disorders patients. Finally, a meta-analysis that systematically summarize cross-cultural data on insomnia prevalence and its association with mental health consequences could be very helpful to have a complete picture of Covid-19 impact in the different countries. This study presents some limitations. Sampling bias cannot be excluded. Our sample included more females than males, and more younger people than elderly and, as mentioned above, the older group showed high health quality. In order to try to control this potential sampling bias gender and age stratified analyses for main parameters of interest were conducted. Furthermore, the questionnaire was relative long, which had caused a number of individuals to not complete all the survey. Nevertheless, no obvious differences between enrolled set and main analysis set was observed. Furthermore, the cross-sectional nature of this study limits an accurate interpretation of the results. Finally, the comparison between pre and during-pandemic data on insomnia prevalence was made comparing data of our study and epidemiological data from 2002 and this could represent a limitation in the interpretation of the results. Indeed, the prevalence of insomnia may have been increased already before the pandemic. Nevertheless, it seems unlikely that such a relevant change in prevalence is independent on life changes caused by the Covid-19 pandemic's outbreak. Several strengths may be also underlined. The sample size was large, thus, allowing us to observe interactions between several variables, and exploring the complexity of real life individual vulnerabilities to insomnia difficulties. Future research should evaluate the effect of Covid-19 spread in Italy after the lockdown in order to assess the trend of insomnia symptoms, anxiety and depression after the worst moments of the health emergency. This would be informative about longitudinal impact of the pandemic on mental health in the Italian population and provide important data for the post-pandemic scenario.

## Data Availability Statement

The raw data supporting the conclusions of this article will be made available by the authors, without undue reservation.

## Ethics Statement

The studies involving human participants were reviewed and approved by Ethic Committee for research in psychological area, University of Study Guglielmo Marconi, Rome (Italy). The patients/participants provided their written informed consent to participate in this study.

## Author Contributions

CBa and VB contributed to the ideas of the research, collection of data, and empirical analysis. MC contributed to the data analysis, design of research methods, and tables. FM, CBu, PD, and DR participated in developing a research design, writing, and interpreting the analysis. All the authors contributed to the literature review and conclusions.

## Conflict of Interest

The authors declare that the research was conducted in the absence of any commercial or financial relationships that could be construed as a potential conflict of interest.
