# Supplementary material for: Insomnia in the Italian Population During Covid-19 Outbreak: A Snapshot on One Major Risk Factor for Depression and Anxiety
Source: Front Psychiatry. 2020 Dec 15;11:579107. doi: 10.3389/fpsyt.2020.579107 (PMC7769843; doi:10.3389/fpsyt.2020.579107)
Supplement: Supplementary file 1 [file Data_Sheet_1.docx]

**Document S1 – Descriptive data for Enrolled Set (ES) and Main Analysis Set (MAS)**

| Table 1.1 - Sex (overall) | |
| --- | --- |
| ES | |
|  | Total (N = 2652) |
| Female | 2023 ( 76.3%) |
| Male | 629 ( 23.7%) |

| Table 1.1 - Sex (overall) | |
| --- | --- |
| MAS | |
|  | Total (N = 1989) |
| Female | 1515 ( 76.2%) |
| Male | 474 ( 23.8%) |

| Table 1.2 - Sex (by age group) | | | |
| --- | --- | --- | --- |
| ES | | | |
|  | Age 18-30 (N = 892) | Age 31-65 (N = 1662) | Age >65 (N = 98) |
| Female | 712 ( 79.8%) | 1252 ( 75.3%) | 59 ( 60.2%) |
| Male | 180 ( 20.2%) | 410 ( 24.7%) | 39 ( 39.8%) |

| Table 1.2 - Sex (by age group) | | | |
| --- | --- | --- | --- |
| MAS | | | |
|  | Age 18-30 (N = 666) | Age 31-65 (N = 1258) | Age >65 (N = 65) |
| Female | 539 ( 80.9%) | 937 ( 74.5%) | 39 ( 60.0%) |
| Male | 127 ( 19.1%) | 321 ( 25.5%) | 26 ( 40.0%) |

| Table 1.3 - Sex (by ISI group) | | | | | |
| --- | --- | --- | --- | --- | --- |
| ES | | | | | |
|  | Absence of insomnia (N = 1247) | Subthreshold insomnia (N = 806) | Moderate insomnia (N = 382) | Severe insomnia (N = 70) | Unknown (N = 147) |
| Female | 911 ( 73.1%) | 648 ( 80.4%) | 293 ( 76.7%) | 55 ( 78.6%) | 116 ( 78.9%) |
| Male | 336 ( 26.9%) | 158 ( 19.6%) | 89 ( 23.3%) | 15 ( 21.4%) | 31 ( 21.1%) |

| Table 1.3 - Sex (by ISI group) | | | | |
| --- | --- | --- | --- | --- |
| MAS | | | | |
|  | Absence of insomnia (N = 977) | Subthreshold insomnia (N = 642) | Moderate insomnia (N = 314) | Severe insomnia (N = 56) |
| Female | 712 ( 72.9%) | 515 ( 80.2%) | 242 ( 77.1%) | 46 ( 82.1%) |
| Male | 265 ( 27.1%) | 127 ( 19.8%) | 72 ( 22.9%) | 10 ( 17.9%) |

| Table 2.1 - Age (overall and by sex) | | | |
| --- | --- | --- | --- |
| ES | | | |
|  | Total (N = 2652) | Female (N = 2023) | Male (N = 629) |
| n | 2652 | 2023 | 629 |
| Mean | 38.5 | 37.7 | 41.0 |
| StD | 13.06 | 12.65 | 14.00 |
| Median | 36.0 | 35.0 | 39.0 |
| 25%/75% quantile | 28.0/47.0 | 28.0/46.0 | 29.0/51.0 |
| Min/Max | 18/90 | 18/86 | 18/90 |

| Table 2.1 - Age (overall and by sex) | | | |
| --- | --- | --- | --- |
| MAS | | | |
|  | Total (N = 1989) | Female (N = 1515) | Male (N = 474) |
| n | 1989 | 1515 | 474 |
| Mean | 38.4 | 37.5 | 41.4 |
| StD | 12.88 | 12.42 | 13.85 |
| Median | 36.0 | 35.0 | 39.0 |
| 25%/75% quantile | 28.0/47.0 | 28.0/45.0 | 30.0/52.0 |
| Min/Max | 18/90 | 18/86 | 18/90 |

| Table 2.2 - Age (by ISI group) | | | | | |
| --- | --- | --- | --- | --- | --- |
| ES | | | | | |
|  | Absence of insomnia (N = 1247) | Subthreshold insomnia (N = 806) | Moderate insomnia (N = 382) | Severe insomnia (N = 70) | Unknown (N = 147) |
| n | 1247 | 806 | 382 | 70 | 147 |
| Mean | 39.9 | 37.5 | 35.9 | 33.9 | 40.3 |
| StD | 13.01 | 12.84 | 12.61 | 10.16 | 14.96 |
| Median | 37.0 | 35.0 | 32.5 | 32.0 | 38.0 |
| 25%/75% quantile | 29.0/48.0 | 27.0/46.8 | 26.0/43.0 | 25.0/39.0 | 28.0/51.0 |
| Min/Max | 18/82 | 18/90 | 18/75 | 18/61 | 18/90 |

| Table 2.2 - Age (by ISI group) | | | | |
| --- | --- | --- | --- | --- |
| MAS | | | | |
|  | Absence of insomnia (N = 977) | Subthreshold insomnia (N = 642) | Moderate insomnia (N = 314) | Severe insomnia (N = 56) |
| n | 977 | 642 | 314 | 56 |
| Mean | 39.9 | 37.6 | 36.1 | 33.4 |
| StD | 12.97 | 12.75 | 12.62 | 10.31 |
| Median | 37.0 | 35.0 | 33.0 | 31.0 |
| 25%/75% quantile | 29.0/49.0 | 28.0/47.0 | 26.0/43.0 | 25.0/39.0 |
| Min/Max | 18/82 | 18/90 | 18/75 | 18/61 |

| Table 3.1 - Age groups (overall and by sex) | | | |
| --- | --- | --- | --- |
| ES | | | |
|  | Total (N = 2652) | Female (N = 2023) | Male (N = 629) |
| 18-30 | 892 ( 33.6%) | 712 ( 35.2%) | 180 ( 28.6%) |
| 31-65 | 1662 ( 62.7%) | 1252 ( 61.9%) | 410 ( 65.2%) |
| >65 | 98 ( 3.7%) | 59 ( 2.9%) | 39 ( 6.2%) |

| Table 3.1 - Age groups (overall and by sex) | | | |
| --- | --- | --- | --- |
| MAS | | | |
|  | Total (N = 1989) | Female (N = 1515) | Male (N = 474) |
| 18-30 | 666 ( 33.5%) | 539 ( 35.6%) | 127 ( 26.8%) |
| 31-65 | 1258 ( 63.2%) | 937 ( 61.8%) | 321 ( 67.7%) |
| >65 | 65 ( 3.3%) | 39 ( 2.6%) | 26 ( 5.5%) |

| Table 3.2 - Age groups (by ISI group) | | | | | |
| --- | --- | --- | --- | --- | --- |
| ES | | | | | |
|  | Absence of insomnia (N = 1247) | Subthreshold insomnia (N = 806) | Moderate insomnia (N = 382) | Severe insomnia (N = 70) | Unknown (N = 147) |
| 18-30 | 353 ( 28.3%) | 301 ( 37.3%) | 160 ( 41.9%) | 27 ( 38.6%) | 51 ( 34.7%) |
| 31-65 | 836 ( 67.0%) | 486 ( 60.3%) | 213 ( 55.8%) | 43 ( 61.4%) | 84 ( 57.1%) |
| >65 | 58 ( 4.7%) | 19 ( 2.4%) | 9 ( 2.4%) | 0 ( 0.0%) | 12 ( 8.2%) |

| Table 3.2 - Age groups (by ISI group) | | | | |
| --- | --- | --- | --- | --- |
| MAS | | | | |
|  | Absence of insomnia (N = 977) | Subthreshold insomnia (N = 642) | Moderate insomnia (N = 314) | Severe insomnia (N = 56) |
| 18-30 | 280 ( 28.7%) | 234 ( 36.4%) | 129 ( 41.1%) | 23 ( 41.1%) |
| 31-65 | 654 ( 66.9%) | 394 ( 61.4%) | 177 ( 56.4%) | 33 ( 58.9%) |
| >65 | 43 ( 4.4%) | 14 ( 2.2%) | 8 ( 2.5%) | 0 ( 0.0%) |

| Table 4.1 - Nationality (overall and by sex) | | | |
| --- | --- | --- | --- |
| ES | | | |
|  | Total (N = 2652) | Female (N = 2023) | Male (N = 629) |
| Italian | 2622 ( 98.9%) | 2002 ( 99.0%) | 620 ( 98.6%) |
| Other | 26 ( 1.0%) | 21 ( 1.0%) | 5 ( 0.8%) |
| Missing | 4 ( 0.2%) | 0 ( 0.0%) | 4 ( 0.6%) |

| Table 4.1 - Nationality (overall and by sex) | | | |
| --- | --- | --- | --- |
| MAS | | | |
|  | Total (N = 1989) | Female (N = 1515) | Male (N = 474) |
| Italian | 1963 ( 98.7%) | 1496 ( 98.7%) | 467 ( 98.5%) |
| Other | 23 ( 1.2%) | 19 ( 1.3%) | 4 ( 0.8%) |
| Missing | 3 ( 0.2%) | 0 ( 0.0%) | 3 ( 0.6%) |

| Table 4.2 - Nationality (by age group) | | | |
| --- | --- | --- | --- |
| ES | | | |
|  | Age 18-30 (N = 892) | Age 31-65 (N = 1662) | Age >65 (N = 98) |
| Italian | 879 ( 98.5%) | 1645 ( 99.0%) | 98 (100.0%) |
| Other | 11 ( 1.2%) | 15 ( 0.9%) | 0 ( 0.0%) |
| Missing | 2 ( 0.2%) | 2 ( 0.1%) | 0 ( 0.0%) |

| Table 4.2 - Nationality (by age group) | | | |
| --- | --- | --- | --- |
| MAS | | | |
|  | Age 18-30 (N = 666) | Age 31-65 (N = 1258) | Age >65 (N = 65) |
| Italian | 657 ( 98.6%) | 1241 ( 98.6%) | 65 (100.0%) |
| Other | 8 ( 1.2%) | 15 ( 1.2%) | 0 ( 0.0%) |
| Missing | 1 ( 0.2%) | 2 ( 0.2%) | 0 ( 0.0%) |

| Table 4.3 - Nationality (by ISI group) | | | | | |
| --- | --- | --- | --- | --- | --- |
| ES | | | | | |
|  | Absence of insomnia (N = 1247) | Subthreshold insomnia (N = 806) | Moderate insomnia (N = 382) | Severe insomnia (N = 70) | Unknown (N = 147) |
| Italian | 1235 ( 99.0%) | 797 ( 98.9%) | 376 ( 98.4%) | 68 ( 97.1%) | 146 ( 99.3%) |
| Other | 12 ( 1.0%) | 6 ( 0.7%) | 6 ( 1.6%) | 2 ( 2.9%) | 0 ( 0.0%) |
| Missing | 0 ( 0.0%) | 3 ( 0.4%) | 0 ( 0.0%) | 0 ( 0.0%) | 1 ( 0.7%) |

| Table 4.3 - Nationality (by ISI group) | | | | |
| --- | --- | --- | --- | --- |
| MAS | | | | |
|  | Absence of insomnia (N = 977) | Subthreshold insomnia (N = 642) | Moderate insomnia (N = 314) | Severe insomnia (N = 56) |
| Italian | 967 ( 99.0%) | 633 ( 98.6%) | 309 ( 98.4%) | 54 ( 96.4%) |
| Other | 10 ( 1.0%) | 6 ( 0.9%) | 5 ( 1.6%) | 2 ( 3.6%) |
| Missing | 0 ( 0.0%) | 3 ( 0.5%) | 0 ( 0.0%) | 0 ( 0.0%) |

| Table 5.1 - Region (overall and by sex) | | | |
| --- | --- | --- | --- |
| ES | | | |
|  | Total (N = 2652) | Female (N = 2023) | Male (N = 629) |
| North | 1025 ( 38.7%) | 797 ( 39.4%) | 228 ( 36.2%) |
| Centre | 965 ( 36.4%) | 707 ( 34.9%) | 258 ( 41.0%) |
| South | 474 ( 17.9%) | 372 ( 18.4%) | 102 ( 16.2%) |
| Islands | 162 ( 6.1%) | 129 ( 6.4%) | 33 ( 5.2%) |
| Unknown | 26 ( 1.0%) | 18 ( 0.9%) | 8 ( 1.3%) |

| Table 5.1 - Region (overall and by sex) | | | |
| --- | --- | --- | --- |
| MAS | | | |
|  | Total (N = 1989) | Female (N = 1515) | Male (N = 474) |
| North | 790 ( 39.7%) | 614 ( 40.5%) | 176 ( 37.1%) |
| Centre | 709 ( 35.6%) | 517 ( 34.1%) | 192 ( 40.5%) |
| South | 353 ( 17.7%) | 278 ( 18.3%) | 75 ( 15.8%) |
| Islands | 122 ( 6.1%) | 96 ( 6.3%) | 26 ( 5.5%) |
| Unknown | 15 ( 0.8%) | 10 ( 0.7%) | 5 ( 1.1%) |

| Table 5.2 - Region (by age group) | | | |
| --- | --- | --- | --- |
| ES | | | |
|  | Age 18-30 (N = 892) | Age 31-65 (N = 1662) | Age >65 (N = 98) |
| North | 343 ( 38.5%) | 660 ( 39.7%) | 22 ( 22.4%) |
| Centre | 289 ( 32.4%) | 609 ( 36.6%) | 67 ( 68.4%) |
| South | 181 ( 20.3%) | 284 ( 17.1%) | 9 ( 9.2%) |
| Islands | 68 ( 7.6%) | 94 ( 5.7%) | 0 ( 0.0%) |
| Unknown | 11 ( 1.2%) | 15 ( 0.9%) | 0 ( 0.0%) |

| Table 5.2 - Region (by age group) | | | |
| --- | --- | --- | --- |
| MAS | | | |
|  | Age 18-30 (N = 666) | Age 31-65 (N = 1258) | Age >65 (N = 65) |
| North | 263 ( 39.5%) | 513 ( 40.8%) | 14 ( 21.5%) |
| Centre | 211 ( 31.7%) | 452 ( 35.9%) | 46 ( 70.8%) |
| South | 137 ( 20.6%) | 211 ( 16.8%) | 5 ( 7.7%) |
| Islands | 50 ( 7.5%) | 72 ( 5.7%) | 0 ( 0.0%) |
| Unknown | 5 ( 0.8%) | 10 ( 0.8%) | 0 ( 0.0%) |

| Table 5.3 - Region (by ISI group) | | | | | |
| --- | --- | --- | --- | --- | --- |
| ES | | | | | |
|  | Absence of insomnia (N = 1247) | Subthreshold insomnia (N = 806) | Moderate insomnia (N = 382) | Severe insomnia (N = 70) | Unknown (N = 147) |
| North | 486 ( 39.0%) | 293 ( 36.4%) | 160 ( 41.9%) | 32 ( 45.7%) | 54 ( 36.7%) |
| Centre | 479 ( 38.4%) | 296 ( 36.7%) | 112 ( 29.3%) | 14 ( 20.0%) | 64 ( 43.5%) |
| South | 215 ( 17.2%) | 148 ( 18.4%) | 73 ( 19.1%) | 18 ( 25.7%) | 20 ( 13.6%) |
| Islands | 56 ( 4.5%) | 61 ( 7.6%) | 33 ( 8.6%) | 6 ( 8.6%) | 6 ( 4.1%) |
| Unknown | 11 ( 0.9%) | 8 ( 1.0%) | 4 ( 1.0%) | 0 ( 0.0%) | 3 ( 2.0%) |

| Table 5.3 - Region (by ISI group) | | | | |
| --- | --- | --- | --- | --- |
| MAS | | | | |
|  | Absence of insomnia (N = 977) | Subthreshold insomnia (N = 642) | Moderate insomnia (N = 314) | Severe insomnia (N = 56) |
| North | 392 ( 40.1%) | 242 ( 37.7%) | 131 ( 41.7%) | 25 ( 44.6%) |
| Centre | 368 ( 37.7%) | 235 ( 36.6%) | 92 ( 29.3%) | 14 ( 25.0%) |
| South | 164 ( 16.8%) | 114 ( 17.8%) | 62 ( 19.7%) | 13 ( 23.2%) |
| Islands | 44 ( 4.5%) | 46 ( 7.2%) | 28 ( 8.9%) | 4 ( 7.1%) |
| Unknown | 9 ( 0.9%) | 5 ( 0.8%) | 1 ( 0.3%) | 0 ( 0.0%) |

| Table 6.1 - BMI (overall and by sex) | | | |
| --- | --- | --- | --- |
| ES | | | |
|  | Total (N = 2652) | Female (N = 2023) | Male (N = 629) |
| n | 2620 | 2000 | 620 |
| Mean | 23.3 | 22.7 | 25.2 |
| StD | 4.10 | 4.07 | 3.60 |
| Median | 22.7 | 21.9 | 24.8 |
| 25%/75% quantile | 20.4/25.3 | 20.0/24.5 | 22.9/27.2 |
| Min/Max | 13/58 | 13/58 | 14/46 |

| Table 6.1 - BMI (overall and by sex) | | | |
| --- | --- | --- | --- |
| MAS | | | |
|  | Total (N = 1989) | Female (N = 1515) | Male (N = 474) |
| n | 1971 | 1501 | 470 |
| Mean | 23.3 | 22.7 | 25.2 |
| StD | 4.14 | 4.13 | 3.56 |
| Median | 22.7 | 21.9 | 24.8 |
| 25%/75% quantile | 20.4/25.4 | 19.9/24.4 | 22.9/27.1 |
| Min/Max | 13/58 | 13/58 | 14/46 |

| Table 6.2 - BMI (by age) | | | |
| --- | --- | --- | --- |
| ES | | | |
|  | Age 18-30 (N = 892) | Age 31-65 (N = 1662) | Age >65 (N = 98) |
| n | 883 | 1642 | 95 |
| Mean | 22.4 | 23.7 | 25.1 |
| StD | 4.08 | 4.03 | 3.88 |
| Median | 21.6 | 23.1 | 24.8 |
| 25%/75% quantile | 19.7/24.0 | 20.8/25.8 | 23.2/27.5 |
| Min/Max | 15/58 | 14/50 | 13/37 |

| Table 6.2 - BMI (by age) | | | |
| --- | --- | --- | --- |
| MAS | | | |
|  | Age 18-30 (N = 666) | Age 31-65 (N = 1258) | Age >65 (N = 65) |
| n | 661 | 1247 | 63 |
| Mean | 22.3 | 23.7 | 24.5 |
| StD | 4.22 | 4.02 | 4.02 |
| Median | 21.6 | 23.2 | 24.6 |
| 25%/75% quantile | 19.7/23.9 | 20.9/25.9 | 21.9/26.8 |
| Min/Max | 15/58 | 14/50 | 13/37 |

| Table 6.3 - BMI (by ISI group) | | | | | | |
| --- | --- | --- | --- | --- | --- | --- |
| ES | | | | | | |
|  | Absence of insomnia (N = 1247) | Subthreshold insomnia (N = 806) | Moderate insomnia (N = 382) | Severe insomnia (N = 70) | Unknown (N = 147) |  |
| n | 1239 | 792 | 377 | 69 | 143 |  |
| Mean | 23.2 | 23.1 | 23.8 | 23.3 | 23.4 |  |
| StD | 3.87 | 4.23 | 4.54 | 4.16 | 4.03 |  |
| Median | 22.7 | 22.4 | 23.1 | 22.3 | 22.9 |  |
| 25%/75% quantile | 20.4/25.3 | 20.3/25.1 | 20.6/26.0 | 20.2/25.7 | 20.7/25.0 |  |
| Min/Max | 13/46 | 14/58 | 15/49 | 18/37 | 18/42 |  |

| Table 6.3 - BMI (by ISI group) | | | | |
| --- | --- | --- | --- | --- |
| MAS | | | | |
|  | Absence of insomnia (N = 977) | Subthreshold insomnia (N = 642) | Moderate insomnia (N = 314) | Severe insomnia (N = 56) |
| n | 971 | 633 | 312 | 55 |
| Mean | 23.2 | 23.2 | 23.7 | 23.0 |
| StD | 3.90 | 4.36 | 4.49 | 3.70 |
| Median | 22.7 | 22.4 | 23.0 | 22.8 |
| 25%/75% quantile | 20.4/25.3 | 20.3/25.3 | 20.5/26.0 | 20.2/25.1 |
| Min/Max | 13/46 | 14/58 | 15/49 | 18/36 |

| Table 8.1 - Family status (overall and by sex) | | | |
| --- | --- | --- | --- |
| ES | | | |
|  | Total (N = 2652) | Female (N = 2023) | Male (N = 629) |
| Alone | 361 ( 13.6%) | 256 ( 12.7%) | 105 ( 16.7%) |
| Other | 107 ( 4.0%) | 73 ( 3.6%) | 34 ( 5.4%) |
| With at least one child below 13 | 499 ( 18.8%) | 390 ( 19.3%) | 109 ( 17.3%) |
| With other children | 269 ( 10.1%) | 199 ( 9.8%) | 70 ( 11.1%) |
| With parent(s) | 720 ( 27.1%) | 570 ( 28.2%) | 150 ( 23.8%) |
| With partner | 696 ( 26.2%) | 535 ( 26.4%) | 161 ( 25.6%) |

| Table 8.1 - Family status (overall and by sex) | | | |
| --- | --- | --- | --- |
| MAS | | | |
|  | Total (N = 1989) | Female (N = 1515) | Male (N = 474) |
| Alone | 263 ( 13.2%) | 190 ( 12.5%) | 73 ( 15.4%) |
| Other | 83 ( 4.2%) | 55 ( 3.6%) | 28 ( 5.9%) |
| With at least one child below 13 | 358 ( 18.0%) | 276 ( 18.2%) | 82 ( 17.3%) |
| With other children | 203 ( 10.2%) | 149 ( 9.8%) | 54 ( 11.4%) |
| With parent(s) | 545 ( 27.4%) | 430 ( 28.4%) | 115 ( 24.3%) |
| With partner | 537 ( 27.0%) | 415 ( 27.4%) | 122 ( 25.7%) |

| Table 8.2 - Family status (by age) | | | |
| --- | --- | --- | --- |
| ES | | | |
|  | Age 18-30 (N = 892) | Age 31-65 (N = 1662) | Age >65 (N = 98) |
| Alone | 82 ( 9.2%) | 259 ( 15.6%) | 20 ( 20.4%) |
| Other | 50 ( 5.6%) | 56 ( 3.4%) | 1 ( 1.0%) |
| With at least one child below 13 | 22 ( 2.5%) | 477 ( 28.7%) | 0 ( 0.0%) |
| With other children | 2 ( 0.2%) | 253 ( 15.2%) | 14 ( 14.3%) |
| With parent(s) | 562 ( 63.0%) | 156 ( 9.4%) | 2 ( 2.0%) |
| With partner | 174 ( 19.5%) | 461 ( 27.7%) | 61 ( 62.2%) |

| Table 8.2 - Family status (by age) | | | |
| --- | --- | --- | --- |
| MAS | | | |
|  | Age 18-30 (N = 666) | Age 31-65 (N = 1258) | Age >65 (N = 65) |
| Alone | 59 ( 8.9%) | 189 ( 15.0%) | 15 ( 23.1%) |
| Other | 37 ( 5.6%) | 45 ( 3.6%) | 1 ( 1.5%) |
| With at least one child below 13 | 15 ( 2.3%) | 343 ( 27.3%) | 0 ( 0.0%) |
| With other children | 0 ( 0.0%) | 192 ( 15.3%) | 11 ( 16.9%) |
| With parent(s) | 419 ( 62.9%) | 124 ( 9.9%) | 2 ( 3.1%) |
| With partner | 136 ( 20.4%) | 365 ( 29.0%) | 36 ( 55.4%) |

| Table 8.3 - Family status (by ISI group) | | | | | |
| --- | --- | --- | --- | --- | --- |
| ES | | | | | |
|  | Absence of insomnia (N = 1247) | Subthreshold insomnia (N = 806) | Moderate insomnia (N = 382) | Severe insomnia (N = 70) | Unknown (N = 147) |
| Alone | 163 ( 13.1%) | 102 ( 12.7%) | 54 ( 14.1%) | 18 ( 25.7%) | 24 ( 16.3%) |
| Other | 41 ( 3.3%) | 38 ( 4.7%) | 15 ( 3.9%) | 7 ( 10.0%) | 6 ( 4.1%) |
| With at least one child below 13 | 257 ( 20.6%) | 137 ( 17.0%) | 66 ( 17.3%) | 9 ( 12.9%) | 30 ( 20.4%) |
| With other children | 126 ( 10.1%) | 81 ( 10.0%) | 38 ( 9.9%) | 4 ( 5.7%) | 20 ( 13.6%) |
| With parent(s) | 292 ( 23.4%) | 240 ( 29.8%) | 130 ( 34.0%) | 26 ( 37.1%) | 32 ( 21.8%) |
| With partner | 368 ( 29.5%) | 208 ( 25.8%) | 79 ( 20.7%) | 6 ( 8.6%) | 35 ( 23.8%) |

| Table 8.3 - Family status (by ISI group) | | | | |
| --- | --- | --- | --- | --- |
| MAS | | | | |
|  | Absence of insomnia (N = 977) | Subthreshold insomnia (N = 642) | Moderate insomnia (N = 314) | Severe insomnia (N = 56) |
| Alone | 129 ( 13.2%) | 81 ( 12.6%) | 41 ( 13.1%) | 12 ( 21.4%) |
| Other | 35 ( 3.6%) | 31 ( 4.8%) | 10 ( 3.2%) | 7 ( 12.5%) |
| With at least one child below 13 | 191 ( 19.5%) | 106 ( 16.5%) | 55 ( 17.5%) | 6 ( 10.7%) |
| With other children | 103 ( 10.5%) | 66 ( 10.3%) | 32 ( 10.2%) | 2 ( 3.6%) |
| With parent(s) | 231 ( 23.6%) | 185 ( 28.8%) | 105 ( 33.4%) | 24 ( 42.9%) |
| With partner | 288 ( 29.5%) | 173 ( 26.9%) | 71 ( 22.6%) | 5 ( 8.9%) |

| Table 9.1 - Number family members (overall and by sex) | | | |
| --- | --- | --- | --- |
| ES | | | |
|  | Total (N = 2652) | Female (N = 2023) | Male (N = 629) |
| 0 | 361 ( 13.6%) | 256 ( 12.7%) | 105 ( 16.7%) |
| 1 | 908 ( 34.2%) | 702 ( 34.7%) | 206 ( 32.8%) |
| 2 | 645 ( 24.3%) | 501 ( 24.8%) | 144 ( 22.9%) |
| 3 | 574 ( 21.6%) | 434 ( 21.5%) | 140 ( 22.3%) |
| 4 | 133 ( 5.0%) | 104 ( 5.1%) | 29 ( 4.6%) |
| 5 | 27 ( 1.0%) | 22 ( 1.1%) | 5 ( 0.8%) |
| 6 | 3 ( 0.1%) | 3 ( 0.1%) | 0 ( 0.0%) |
| 7 | 1 ( 0.0%) | 1 ( 0.0%) | 0 ( 0.0%) |

| Table 9.1 - Number family members (overall and by sex) | | | |
| --- | --- | --- | --- |
| MAS | | | |
|  | Total (N = 1989) | Female (N = 1515) | Male (N = 474) |
| 0 | 263 ( 13.2%) | 190 ( 12.5%) | 73 ( 15.4%) |
| 1 | 687 ( 34.5%) | 533 ( 35.2%) | 154 ( 32.5%) |
| 2 | 496 ( 24.9%) | 384 ( 25.3%) | 112 ( 23.6%) |
| 3 | 421 ( 21.2%) | 312 ( 20.6%) | 109 ( 23.0%) |
| 4 | 98 ( 4.9%) | 76 ( 5.0%) | 22 ( 4.6%) |
| 5 | 21 ( 1.1%) | 17 ( 1.1%) | 4 ( 0.8%) |
| 6 | 2 ( 0.1%) | 2 ( 0.1%) | 0 ( 0.0%) |
| 7 | 1 ( 0.1%) | 1 ( 0.1%) | 0 ( 0.0%) |

| Table 9.2 - Number family members (by age) | | | |
| --- | --- | --- | --- |
| ES | | | |
|  | Age 18-30 (N = 892) | Age 31-65 (N = 1662) | Age >65 (N = 98) |
| 0 | 82 ( 9.2%) | 259 ( 15.6%) | 20 ( 20.4%) |
| 1 | 264 ( 29.6%) | 578 ( 34.8%) | 66 ( 67.3%) |
| 2 | 241 ( 27.0%) | 398 ( 23.9%) | 6 ( 6.1%) |
| 3 | 221 ( 24.8%) | 348 ( 20.9%) | 5 ( 5.1%) |
| 4 | 71 ( 8.0%) | 62 ( 3.7%) | 0 ( 0.0%) |
| 5 | 11 ( 1.2%) | 16 ( 1.0%) | 0 ( 0.0%) |
| 6 | 1 ( 0.1%) | 1 ( 0.1%) | 1 ( 1.0%) |
| 7 | 1 ( 0.1%) | 0 ( 0.0%) | 0 ( 0.0%) |

| Table 9.2 - Number family members (by age) | | | |
| --- | --- | --- | --- |
| MAS | | | |
|  | Age 18-30 (N = 666) | Age 31-65 (N = 1258) | Age >65 (N = 65) |
| 0 | 59 ( 8.9%) | 189 ( 15.0%) | 15 ( 23.1%) |
| 1 | 198 ( 29.7%) | 450 ( 35.8%) | 39 ( 60.0%) |
| 2 | 191 ( 28.7%) | 299 ( 23.8%) | 6 ( 9.2%) |
| 3 | 156 ( 23.4%) | 261 ( 20.7%) | 4 ( 6.2%) |
| 4 | 52 ( 7.8%) | 46 ( 3.7%) | 0 ( 0.0%) |
| 5 | 8 ( 1.2%) | 13 ( 1.0%) | 0 ( 0.0%) |
| 6 | 1 ( 0.2%) | 0 ( 0.0%) | 1 ( 1.5%) |
| 7 | 1 ( 0.2%) | 0 ( 0.0%) | 0 ( 0.0%) |

| Table 9.3 - Number family members (by ISI group) | | | | | |
| --- | --- | --- | --- | --- | --- |
| ES | | | | | |
|  | Absence of insomnia (N = 1247) | Subthreshold insomnia (N = 806) | Moderate insomnia (N = 382) | Severe insomnia (N = 70) | Unknown (N = 147) |
| 0 | 163 ( 13.1%) | 102 ( 12.7%) | 54 ( 14.1%) | 18 ( 25.7%) | 24 ( 16.3%) |
| 1 | 454 ( 36.4%) | 283 ( 35.1%) | 109 ( 28.5%) | 17 ( 24.3%) | 45 ( 30.6%) |
| 2 | 294 ( 23.6%) | 203 ( 25.2%) | 98 ( 25.7%) | 19 ( 27.1%) | 31 ( 21.1%) |
| 3 | 261 ( 20.9%) | 172 ( 21.3%) | 88 ( 23.0%) | 11 ( 15.7%) | 42 ( 28.6%) |
| 4 | 61 ( 4.9%) | 40 ( 5.0%) | 24 ( 6.3%) | 4 ( 5.7%) | 4 ( 2.7%) |
| 5 | 12 ( 1.0%) | 5 ( 0.6%) | 8 ( 2.1%) | 1 ( 1.4%) | 1 ( 0.7%) |
| 6 | 1 ( 0.1%) | 1 ( 0.1%) | 1 ( 0.3%) | 0 ( 0.0%) | 0 ( 0.0%) |
| 7 | 1 ( 0.1%) | 0 ( 0.0%) | 0 ( 0.0%) | 0 ( 0.0%) | 0 ( 0.0%) |

| Table 9.3 - Number family members (by ISI group) | | | | |
| --- | --- | --- | --- | --- |
| MAS | | | | |
|  | Absence of insomnia (N = 977) | Subthreshold insomnia (N = 642) | Moderate insomnia (N = 314) | Severe insomnia (N = 56) |
| 0 | 129 ( 13.2%) | 81 ( 12.6%) | 41 ( 13.1%) | 12 ( 21.4%) |
| 1 | 351 ( 35.9%) | 230 ( 35.8%) | 91 ( 29.0%) | 15 ( 26.8%) |
| 2 | 231 ( 23.6%) | 166 ( 25.9%) | 84 ( 26.8%) | 15 ( 26.8%) |
| 3 | 209 ( 21.4%) | 130 ( 20.2%) | 72 ( 22.9%) | 10 ( 17.9%) |
| 4 | 45 ( 4.6%) | 30 ( 4.7%) | 20 ( 6.4%) | 3 ( 5.4%) |
| 5 | 11 ( 1.1%) | 4 ( 0.6%) | 5 ( 1.6%) | 1 ( 1.8%) |
| 6 | 0 ( 0.0%) | 1 ( 0.2%) | 1 ( 0.3%) | 0 ( 0.0%) |
| 7 | 1 ( 0.1%) | 0 ( 0.0%) | 0 ( 0.0%) | 0 ( 0.0%) |

| Table 11.1 - Occupation (overall and by sex) | | | |
| --- | --- | --- | --- |
| ES | | | |
|  | Total (N = 2652) | Female (N = 2023) | Male (N = 629) |
| Student | 363 ( 13.7%) | 309 ( 15.3%) | 54 ( 8.6%) |
| Employed | 1565 ( 59.0%) | 1156 ( 57.1%) | 409 ( 65.0%) |
| Physician/health operator | 133 ( 5.0%) | 111 ( 5.5%) | 22 ( 3.5%) |
| Homemaker | 48 ( 1.8%) | 47 ( 2.3%) | 1 ( 0.2%) |
| Unemployed | 137 ( 5.2%) | 114 ( 5.6%) | 23 ( 3.7%) |
| Retired | 86 ( 3.2%) | 53 ( 2.6%) | 33 ( 5.2%) |
| Other | 310 ( 11.7%) | 226 ( 11.2%) | 84 ( 13.4%) |
| Missing | 10 ( 0.4%) | 7 ( 0.3%) | 3 ( 0.5%) |

| Table 11.1 - Occupation (overall and by sex) | | | |
| --- | --- | --- | --- |
| MAS | | | |
|  | Total (N = 1989) | Female (N = 1515) | Male (N = 474) |
| Student | 269 ( 13.5%) | 228 ( 15.0%) | 41 ( 8.6%) |
| Employed | 1174 ( 59.0%) | 863 ( 57.0%) | 311 ( 65.6%) |
| Physician/health operator | 103 ( 5.2%) | 87 ( 5.7%) | 16 ( 3.4%) |
| Homemaker | 32 ( 1.6%) | 31 ( 2.0%) | 1 ( 0.2%) |
| Unemployed | 96 ( 4.8%) | 81 ( 5.3%) | 15 ( 3.2%) |
| Retired | 61 ( 3.1%) | 35 ( 2.3%) | 26 ( 5.5%) |
| Other | 250 ( 12.6%) | 187 ( 12.3%) | 63 ( 13.3%) |
| Missing | 4 ( 0.2%) | 3 ( 0.2%) | 1 ( 0.2%) |

| Table 11.2 - Occupation (by age) | | | |
| --- | --- | --- | --- |
| ES | | | |
|  | Age 18-30 (N = 892) | Age 31-65 (N = 1662) | Age >65 (N = 98) |
| Student | 314 ( 35.2%) | 49 ( 2.9%) | 0 ( 0.0%) |
| Employed | 377 ( 42.3%) | 1170 ( 70.4%) | 18 ( 18.4%) |
| Physician/health operator | 12 ( 1.3%) | 116 ( 7.0%) | 5 ( 5.1%) |
| Homemaker | 7 ( 0.8%) | 37 ( 2.2%) | 4 ( 4.1%) |
| Unemployed | 71 ( 8.0%) | 66 ( 4.0%) | 0 ( 0.0%) |
| Retired | 0 ( 0.0%) | 22 ( 1.3%) | 64 ( 65.3%) |
| Other | 106 ( 11.9%) | 197 ( 11.9%) | 7 ( 7.1%) |
| Missing | 5 ( 0.6%) | 5 ( 0.3%) | 0 ( 0.0%) |

| Table 11.2 - Occupation (by age) | | | |
| --- | --- | --- | --- |
| MAS | | | |
|  | Age 18-30 (N = 666) | Age 31-65 (N = 1258) | Age >65 (N = 65) |
| Student | 229 ( 34.4%) | 40 ( 3.2%) | 0 ( 0.0%) |
| Employed | 271 ( 40.7%) | 890 ( 70.7%) | 13 ( 20.0%) |
| Physician/health operator | 10 ( 1.5%) | 90 ( 7.2%) | 3 ( 4.6%) |
| Homemaker | 6 ( 0.9%) | 25 ( 2.0%) | 1 ( 1.5%) |
| Unemployed | 57 ( 8.6%) | 39 ( 3.1%) | 0 ( 0.0%) |
| Retired | 0 ( 0.0%) | 18 ( 1.4%) | 43 ( 66.2%) |
| Other | 92 ( 13.8%) | 153 ( 12.2%) | 5 ( 7.7%) |
| Missing | 1 ( 0.2%) | 3 ( 0.2%) | 0 ( 0.0%) |

| Table 11.3 - Occupation (by ISI group) | | | | | |
| --- | --- | --- | --- | --- | --- |
| ES | | | | | |
|  | Absence of insomnia (N = 1247) | Subthreshold insomnia (N = 806) | Moderate insomnia (N = 382) | Severe insomnia (N = 70) | Unknown (N = 147) |
| Student | 131 ( 10.5%) | 115 ( 14.3%) | 82 ( 21.5%) | 15 ( 21.4%) | 20 ( 13.6%) |
| Employed | 782 ( 62.7%) | 454 ( 56.3%) | 203 ( 53.1%) | 37 ( 52.9%) | 89 ( 60.5%) |
| Physician/health operator | 63 ( 5.1%) | 44 ( 5.5%) | 20 ( 5.2%) | 1 ( 1.4%) | 5 ( 3.4%) |
| Homemaker | 17 ( 1.4%) | 20 ( 2.5%) | 6 ( 1.6%) | 0 ( 0.0%) | 5 ( 3.4%) |
| Unemployed | 49 ( 3.9%) | 49 ( 6.1%) | 27 ( 7.1%) | 8 ( 11.4%) | 4 ( 2.7%) |
| Retired | 53 ( 4.3%) | 17 ( 2.1%) | 8 ( 2.1%) | 0 ( 0.0%) | 8 ( 5.4%) |
| Other | 150 ( 12.0%) | 104 ( 12.9%) | 35 ( 9.2%) | 9 ( 12.9%) | 12 ( 8.2%) |
| Missing | 2 ( 0.2%) | 3 ( 0.4%) | 1 ( 0.3%) | 0 ( 0.0%) | 4 ( 2.7%) |

| Table 11.3 - Occupation (by ISI group) | | | | |
| --- | --- | --- | --- | --- |
| MAS | | | | |
|  | Absence of insomnia (N = 977) | Subthreshold insomnia (N = 642) | Moderate insomnia (N = 314) | Severe insomnia (N = 56) |
| Student | 103 ( 10.5%) | 86 ( 13.4%) | 68 ( 21.7%) | 12 ( 21.4%) |
| Employed | 615 ( 62.9%) | 361 ( 56.2%) | 168 ( 53.5%) | 30 ( 53.6%) |
| Physician/health operator | 49 ( 5.0%) | 38 ( 5.9%) | 15 ( 4.8%) | 1 ( 1.8%) |
| Homemaker | 10 ( 1.0%) | 16 ( 2.5%) | 6 ( 1.9%) | 0 ( 0.0%) |
| Unemployed | 33 ( 3.4%) | 37 ( 5.8%) | 21 ( 6.7%) | 5 ( 8.9%) |
| Retired | 42 ( 4.3%) | 11 ( 1.7%) | 8 ( 2.5%) | 0 ( 0.0%) |
| Other | 124 ( 12.7%) | 91 ( 14.2%) | 27 ( 8.6%) | 8 ( 14.3%) |
| Missing | 1 ( 0.1%) | 2 ( 0.3%) | 1 ( 0.3%) | 0 ( 0.0%) |

| Table 14.1 - Work satisfaction general (overall and by sex) | | | |
| --- | --- | --- | --- |
| ES | | | |
|  | Total (N = 2652) | Female (N = 2023) | Male (N = 629) |
| n | 2094 | 1557 | 537 |
| Mean | 69.0 | 68.0 | 72.2 |
| StD | 24.31 | 24.64 | 23.07 |
| Median | 75.0 | 75.0 | 78.0 |
| 25%/75% quantile | 54.0/86.0 | 52.0/85.0 | 60.0/90.0 |
| Min/Max | 0/100 | 0/100 | 0/100 |

| Table 14.1 - Work satisfaction general (overall and by sex) | | | |
| --- | --- | --- | --- |
| MAS | | | |
|  | Total (N = 1989) | Female (N = 1515) | Male (N = 474) |
| n | 1597 | 1190 | 407 |
| Mean | 68.6 | 67.3 | 72.2 |
| StD | 24.31 | 24.58 | 23.13 |
| Median | 75.0 | 72.5 | 78.0 |
| 25%/75% quantile | 53.0/85.0 | 51.0/85.0 | 62.5/90.0 |
| Min/Max | 0/100 | 0/100 | 0/100 |

| Table 14.2 - Work satisfaction general (by age) | | | |
| --- | --- | --- | --- |
| ES | | | |
|  | Age 18-30 (N = 892) | Age 31-65 (N = 1662) | Age >65 (N = 98) |
| n | 557 | 1506 | 31 |
| Mean | 66.9 | 69.7 | 77.8 |
| StD | 25.90 | 23.68 | 22.18 |
| Median | 73.0 | 75.0 | 81.0 |
| 25%/75% quantile | 51.0/85.0 | 55.0/87.0 | 75.0/89.5 |
| Min/Max | 0/100 | 0/100 | 0/100 |

| Table 14.2 - Work satisfaction general (by age) | | | |
| --- | --- | --- | --- |
| MAS | | | |
|  | Age 18-30 (N = 666) | Age 31-65 (N = 1258) | Age >65 (N = 65) |
| n | 421 | 1156 | 20 |
| Mean | 66.8 | 69.1 | 73.3 |
| StD | 26.04 | 23.61 | 25.29 |
| Median | 73.0 | 75.0 | 80.0 |
| 25%/75% quantile | 50.0/85.0 | 54.0/85.0 | 74.0/86.0 |
| Min/Max | 0/100 | 0/100 | 0/ 96 |

| Table 14.3 - Work satisfaction general (by ISI group) | | | | | |
| --- | --- | --- | --- | --- | --- |
| ES | | | | | |
|  | Absence of insomnia (N = 1247) | Subthreshold insomnia (N = 806) | Moderate insomnia (N = 382) | Severe insomnia (N = 70) | Unknown (N = 147) |
| n | 1015 | 629 | 286 | 54 | 110 |
| Mean | 72.4 | 65.5 | 65.7 | 59.1 | 71.7 |
| StD | 22.66 | 24.35 | 26.21 | 31.72 | 24.78 |
| Median | 79.0 | 70.0 | 70.0 | 67.0 | 79.0 |
| 25%/75% quantile | 60.0/90.0 | 50.0/81.0 | 50.0/85.0 | 37.8/83.8 | 60.0/89.5 |
| Min/Max | 0/100 | 0/100 | 0/100 | 0/100 | 0/100 |

| Table 14.3 - Work satisfaction general (by ISI group) | | | | |
| --- | --- | --- | --- | --- |
| MAS | | | | |
|  | Absence of insomnia (N = 977) | Subthreshold insomnia (N = 642) | Moderate insomnia (N = 314) | Severe insomnia (N = 56) |
| n | 807 | 509 | 236 | 45 |
| Mean | 72.6 | 65.2 | 63.9 | 59.4 |
| StD | 22.60 | 24.26 | 26.12 | 31.65 |
| Median | 80.0 | 70.0 | 70.0 | 70.0 |
| 25%/75% quantile | 62.0/90.0 | 50.0/80.0 | 50.0/81.2 | 37.0/85.0 |
| Min/Max | 0/100 | 0/100 | 0/100 | 0/100 |

| Table 15.1 - Current work satisfaction (overall and by sex) | | | |
| --- | --- | --- | --- |
| ES | | | |
|  | Total (N = 2652) | Female (N = 2023) | Male (N = 629) |
| n | 2080 | 1545 | 535 |
| Mean | 54.7 | 53.1 | 59.3 |
| StD | 29.27 | 29.25 | 28.85 |
| Median | 59.0 | 54.0 | 65.0 |
| 25%/75% quantile | 31.0/80.0 | 30.0/78.0 | 40.0/80.0 |
| Min/Max | 0/100 | 0/100 | 0/100 |

| Table 15.1 - Current work satisfaction (overall and by sex) | | | |
| --- | --- | --- | --- |
| MAS | | | |
|  | Total (N = 1989) | Female (N = 1515) | Male (N = 474) |
| n | 1584 | 1179 | 405 |
| Mean | 54.1 | 52.3 | 59.1 |
| StD | 29.21 | 29.21 | 28.63 |
| Median | 57.5 | 53.0 | 65.0 |
| 25%/75% quantile | 30.0/80.0 | 30.0/77.0 | 40.0/80.0 |
| Min/Max | 0/100 | 0/100 | 0/100 |

| Table 15.2 - Current work satisfaction (by age) | | | |
| --- | --- | --- | --- |
| ES | | | |
|  | Age 18-30 (N = 892) | Age 31-65 (N = 1662) | Age >65 (N = 98) |
| n | 557 | 1492 | 31 |
| Mean | 50.8 | 56.1 | 55.3 |
| StD | 31.20 | 28.34 | 31.72 |
| Median | 52.0 | 60.0 | 60.0 |
| 25%/75% quantile | 24.0/76.0 | 36.0/80.0 | 36.0/81.0 |
| Min/Max | 0/100 | 0/100 | 0/100 |

| Table 15.2 - Current work satisfaction (by age) | | | |
| --- | --- | --- | --- |
| MAS | | | |
|  | Age 18-30 (N = 666) | Age 31-65 (N = 1258) | Age >65 (N = 65) |
| n | 421 | 1143 | 20 |
| Mean | 49.9 | 55.6 | 53.1 |
| StD | 31.17 | 28.25 | 32.91 |
| Median | 51.0 | 60.0 | 55.0 |
| 25%/75% quantile | 23.0/75.0 | 35.0/80.0 | 28.0/80.8 |
| Min/Max | 0/100 | 0/100 | 0/100 |

| Table 15.3 - Current work satisfaction (by ISI group) | | | | | |
| --- | --- | --- | --- | --- | --- |
| ES | | | | | |
|  | Absence of insomnia (N = 1247) | Subthreshold insomnia (N = 806) | Moderate insomnia (N = 382) | Severe insomnia (N = 70) | Unknown (N = 147) |
| n | 1007 | 625 | 284 | 54 | 110 |
| Mean | 57.9 | 51.8 | 51.0 | 41.9 | 56.8 |
| StD | 28.62 | 29.32 | 29.36 | 30.68 | 29.84 |
| Median | 60.0 | 53.0 | 53.0 | 42.0 | 55.5 |
| 25%/75% quantile | 40.0/80.0 | 30.0/77.0 | 29.5/75.0 | 13.0/66.5 | 39.2/80.0 |
| Min/Max | 0/100 | 0/100 | 0/100 | 0/100 | 0/100 |

| Table 15.3 - Current work satisfaction (by ISI group) | | | | |
| --- | --- | --- | --- | --- |
| MAS | | | | |
|  | Absence of insomnia (N = 977) | Subthreshold insomnia (N = 642) | Moderate insomnia (N = 314) | Severe insomnia (N = 56) |
| n | 799 | 506 | 234 | 45 |
| Mean | 57.9 | 51.1 | 50.0 | 40.9 |
| StD | 28.80 | 28.86 | 29.32 | 30.22 |
| Median | 61.0 | 51.0 | 53.0 | 41.0 |
| 25%/75% quantile | 40.0/80.0 | 29.0/75.0 | 26.2/74.8 | 13.0/63.0 |
| Min/Max | 0/100 | 0/100 | 0/100 | 0/100 |

| Table 16.1 - Change in work satisfaction (overall and by sex) | | | |
| --- | --- | --- | --- |
| ES | | | |
|  | Total (N = 2652) | Female (N = 2023) | Male (N = 629) |
| n | 2070 | 1537 | 533 |
| Mean | -14.4 | -15.0 | -12.9 |
| StD | 24.55 | 24.68 | 24.14 |
| Median | -6.0 | -8.0 | -2.0 |
| 25%/75% quantile | -26.0/0.0 | -27.0/0.0 | -22.0/0.0 |
| Min/Max | -100/90 | -100/90 | -100/50 |

| Table 16.1 - Change in work satisfaction (overall and by sex) | | | |
| --- | --- | --- | --- |
| MAS | | | |
|  | Total (N = 1989) | Female (N = 1515) | Male (N = 474) |
| n | 1578 | 1175 | 403 |
| Mean | -14.7 | -15.2 | -13.2 |
| StD | 24.52 | 24.83 | 23.56 |
| Median | -7.0 | -8.0 | -5.0 |
| 25%/75% quantile | -26.0/0.0 | -27.0/0.0 | -22.5/0.0 |
| Min/Max | -100/83 | -100/83 | -100/50 |

| Table 16.2 - Change in work satisfaction (by age) | | | |
| --- | --- | --- | --- |
| ES | | | |
|  | Age 18-30 (N = 892) | Age 31-65 (N = 1662) | Age >65 (N = 98) |
| n | 551 | 1488 | 31 |
| Mean | -15.9 | -13.7 | -22.5 |
| StD | 26.52 | 23.42 | 36.99 |
| Median | -7.0 | -6.0 | -10.0 |
| 25%/75% quantile | -30.0/0.0 | -25.0/0.0 | -54.0/0.0 |
| Min/Max | -100/43 | -100/90 | -92/83 |

| Table 16.2 - Change in work satisfaction (by age) | | | |
| --- | --- | --- | --- |
| MAS | | | |
|  | Age 18-30 (N = 666) | Age 31-65 (N = 1258) | Age >65 (N = 65) |
| n | 417 | 1141 | 20 |
| Mean | -16.8 | -13.8 | -20.2 |
| StD | 27.01 | 23.18 | 38.99 |
| Median | -9.0 | -6.0 | -15.0 |
| 25%/75% quantile | -30.0/0.0 | -25.0/0.0 | -43.2/1.2 |
| Min/Max | -100/43 | -100/73 | -92/83 |

| Table 16.3 - Change in work satisfaction (by ISI group) | | | | | |
| --- | --- | --- | --- | --- | --- |
| ES | | | | | |
|  | Absence of insomnia (N = 1247) | Subthreshold insomnia (N = 806) | Moderate insomnia (N = 382) | Severe insomnia (N = 70) | Unknown (N = 147) |
| n | 1002 | 624 | 281 | 54 | 109 |
| Mean | -14.4 | -13.9 | -15.1 | -17.3 | -14.5 |
| StD | 24.23 | 24.51 | 25.40 | 27.53 | 24.33 |
| Median | -6.0 | -6.5 | -7.0 | -3.0 | -6.0 |
| 25%/75% quantile | -26.0/0.0 | -25.0/0.0 | -27.0/0.0 | -32.8/0.0 | -27.0/0.0 |
| Min/Max | -100/90 | -100/83 | -100/49 | -80/39 | -90/40 |

| Table 16.3 - Change in work satisfaction (by ISI group) | | | | |
| --- | --- | --- | --- | --- |
| MAS | | | | |
|  | Absence of insomnia (N = 977) | Subthreshold insomnia (N = 642) | Moderate insomnia (N = 314) | Severe insomnia (N = 56) |
| n | 796 | 505 | 232 | 45 |
| Mean | -14.8 | -14.3 | -14.6 | -18.5 |
| StD | 24.31 | 24.70 | 24.11 | 28.50 |
| Median | -5.0 | -8.0 | -7.0 | -4.0 |
| 25%/75% quantile | -26.0/0.0 | -25.0/0.0 | -27.0/0.0 | -34.0/0.0 |
| Min/Max | -100/50 | -100/83 | -93/49 | -80/39 |

| Table 19.1 - More use of devices (overall and by sex) | | | |
| --- | --- | --- | --- |
| ES | | | |
|  | Total (N = 2652) | Female (N = 2023) | Male (N = 629) |
| Yes | 2191 ( 82.6%) | 1722 ( 85.1%) | 469 ( 74.6%) |
| No | 437 ( 16.5%) | 284 ( 14.0%) | 153 ( 24.3%) |
| Missing | 24 ( 0.9%) | 17 ( 0.8%) | 7 ( 1.1%) |

| Table 19.1 - More use of devices (overall and by sex) | | | |
| --- | --- | --- | --- |
| MAS | | | |
|  | Total (N = 1989) | Female (N = 1515) | Male (N = 474) |
| Yes | 1640 ( 82.5%) | 1290 ( 85.1%) | 350 ( 73.8%) |
| No | 334 ( 16.8%) | 213 ( 14.1%) | 121 ( 25.5%) |
| Missing | 15 ( 0.8%) | 12 ( 0.8%) | 3 ( 0.6%) |

| Table 19.2 - More use of devices (by age) | | | |
| --- | --- | --- | --- |
| ES | | | |
|  | Age 18-30 (N = 892) | Age 31-65 (N = 1662) | Age >65 (N = 98) |
| Yes | 753 ( 84.4%) | 1366 ( 82.2%) | 72 ( 73.5%) |
| No | 131 ( 14.7%) | 284 ( 17.1%) | 22 ( 22.4%) |
| Missing | 8 ( 0.9%) | 12 ( 0.7%) | 4 ( 4.1%) |

| Table 19.2 - More use of devices (by age) | | | |
| --- | --- | --- | --- |
| MAS | | | |
|  | Age 18-30 (N = 666) | Age 31-65 (N = 1258) | Age >65 (N = 65) |
| Yes | 563 ( 84.5%) | 1033 ( 82.1%) | 44 ( 67.7%) |
| No | 98 ( 14.7%) | 218 ( 17.3%) | 18 ( 27.7%) |
| Missing | 5 ( 0.8%) | 7 ( 0.6%) | 3 ( 4.6%) |

| Table 19.3 - More use of devices (non-working) (by ISI group) | | | | | |
| --- | --- | --- | --- | --- | --- |
| ES | | | | | |
|  | Absence of insomnia (N = 1247) | Subthreshold insomnia (N = 806) | Moderate insomnia (N = 382) | Severe insomnia (N = 70) | Unknown (N = 147) |
| Yes | 1000 ( 80.2%) | 687 ( 85.2%) | 326 ( 85.3%) | 59 ( 84.3%) | 119 ( 81.0%) |
| No | 238 ( 19.1%) | 116 ( 14.4%) | 51 ( 13.4%) | 9 ( 12.9%) | 23 ( 15.6%) |
| Missing | 9 ( 0.7%) | 3 ( 0.4%) | 5 ( 1.3%) | 2 ( 2.9%) | 5 ( 3.4%) |

| Table 19.3 - More use of devices (non-working) (by ISI group) | | | | |
| --- | --- | --- | --- | --- |
| MAS | | | | |
|  | Absence of insomnia (N = 977) | Subthreshold insomnia (N = 642) | Moderate insomnia (N = 314) | Severe insomnia (N = 56) |
| Yes | 787 ( 80.6%) | 544 ( 84.7%) | 263 ( 83.8%) | 46 ( 82.1%) |
| No | 184 ( 18.8%) | 95 ( 14.8%) | 47 ( 15.0%) | 8 ( 14.3%) |
| Missing | 6 ( 0.6%) | 3 ( 0.5%) | 4 ( 1.3%) | 2 ( 3.6%) |

| Table 20.1 - Exercise before (overall and by sex) | | | |
| --- | --- | --- | --- |
| ES | | | |
|  | Total (N = 2652) | Female (N = 2023) | Male (N = 629) |
| Yes | 1746 ( 65.8%) | 1314 ( 65.0%) | 432 ( 68.7%) |
| No | 895 ( 33.7%) | 703 ( 34.8%) | 192 ( 30.5%) |
| Missing | 11 ( 0.4%) | 6 ( 0.3%) | 5 ( 0.8%) |

| Table 20.1 - Exercise before (overall and by sex) | | | |
| --- | --- | --- | --- |
| MAS | | | |
|  | Total (N = 1989) | Female (N = 1515) | Male (N = 474) |
| Yes | 1315 ( 66.1%) | 992 ( 65.5%) | 323 ( 68.1%) |
| No | 669 ( 33.6%) | 519 ( 34.3%) | 150 ( 31.6%) |
| Missing | 5 ( 0.3%) | 4 ( 0.3%) | 1 ( 0.2%) |

| Table 20.2 - Exercise before (by age) | | | |
| --- | --- | --- | --- |
| ES | | | |
|  | Age 18-30 (N = 892) | Age 31-65 (N = 1662) | Age >65 (N = 98) |
| Yes | 591 ( 66.3%) | 1080 ( 65.0%) | 75 ( 76.5%) |
| No | 296 ( 33.2%) | 577 ( 34.7%) | 22 ( 22.4%) |
| Missing | 5 ( 0.6%) | 5 ( 0.3%) | 1 ( 1.0%) |

| Table 20.2 - Exercise before (by age) | | | |
| --- | --- | --- | --- |
| MAS | | | |
|  | Age 18-30 (N = 666) | Age 31-65 (N = 1258) | Age >65 (N = 65) |
| Yes | 438 ( 65.8%) | 826 ( 65.7%) | 51 ( 78.5%) |
| No | 226 ( 33.9%) | 429 ( 34.1%) | 14 ( 21.5%) |
| Missing | 2 ( 0.3%) | 3 ( 0.2%) | 0 ( 0.0%) |

| Table 20.3 - Exercise before (by ISI group) | | | | | |
| --- | --- | --- | --- | --- | --- |
| ES | | | | | |
|  | Absence of insomnia (N = 1247) | Subthreshold insomnia (N = 806) | Moderate insomnia (N = 382) | Severe insomnia (N = 70) | Unknown (N = 147) |
| Yes | 831 ( 66.6%) | 520 ( 64.5%) | 251 ( 65.7%) | 49 ( 70.0%) | 95 ( 64.6%) |
| No | 414 ( 33.2%) | 284 ( 35.2%) | 129 ( 33.8%) | 21 ( 30.0%) | 47 ( 32.0%) |
| Missing | 2 ( 0.2%) | 2 ( 0.2%) | 2 ( 0.5%) | 0 ( 0.0%) | 5 ( 3.4%) |

| Table 20.3 - Exercise before (by ISI group) | | | | |
| --- | --- | --- | --- | --- |
| MAS | | | | |
|  | Absence of insomnia (N = 977) | Subthreshold insomnia (N = 642) | Moderate insomnia (N = 314) | Severe insomnia (N = 56) |
| Yes | 664 ( 68.0%) | 407 ( 63.4%) | 204 ( 65.0%) | 40 ( 71.4%) |
| No | 312 ( 31.9%) | 233 ( 36.3%) | 108 ( 34.4%) | 16 ( 28.6%) |
| Missing | 1 ( 0.1%) | 2 ( 0.3%) | 2 ( 0.6%) | 0 ( 0.0%) |

| Table 22.1 - Exercise now (overall and by sex) | | | |
| --- | --- | --- | --- |
| ES | | | |
|  | Total (N = 2652) | Female (N = 2023) | Male (N = 629) |
| Yes | 1325 ( 50.0%) | 1029 ( 50.9%) | 296 ( 47.1%) |
| No | 526 ( 19.8%) | 376 ( 18.6%) | 150 ( 23.8%) |
| Missing | 801 ( 30.2%) | 618 ( 30.5%) | 183 ( 29.1%) |

| Table 22.1 - Exercise now (overall and by sex) | | | |
| --- | --- | --- | --- |
| MAS | | | |
|  | Total (N = 1989) | Female (N = 1515) | Male (N = 474) |
| Yes | 989 ( 49.7%) | 773 ( 51.0%) | 216 ( 45.6%) |
| No | 402 ( 20.2%) | 288 ( 19.0%) | 114 ( 24.1%) |
| Missing | 598 ( 30.1%) | 454 ( 30.0%) | 144 ( 30.4%) |

| Table 22.2 - Exercise now (by age) | | | |
| --- | --- | --- | --- |
| ES | | | |
|  | Age 18-30 (N = 892) | Age 31-65 (N = 1662) | Age >65 (N = 98) |
| Yes | 475 ( 53.3%) | 802 ( 48.3%) | 48 ( 49.0%) |
| No | 143 ( 16.0%) | 355 ( 21.4%) | 28 ( 28.6%) |
| Missing | 274 ( 30.7%) | 505 ( 30.4%) | 22 ( 22.4%) |

| Table 22.2 - Exercise now (by age) | | | |
| --- | --- | --- | --- |
| MAS | | | |
|  | Age 18-30 (N = 666) | Age 31-65 (N = 1258) | Age >65 (N = 65) |
| Yes | 344 ( 51.7%) | 614 ( 48.8%) | 31 ( 47.7%) |
| No | 113 ( 17.0%) | 268 ( 21.3%) | 21 ( 32.3%) |
| Missing | 209 ( 31.4%) | 376 ( 29.9%) | 13 ( 20.0%) |

| Table 22.3 - Exercise now (by ISI group) | | | | | |
| --- | --- | --- | --- | --- | --- |
| ES | | | | | |
|  | Absence of insomnia (N = 1247) | Subthreshold insomnia (N = 806) | Moderate insomnia (N = 382) | Severe insomnia (N = 70) | Unknown (N = 147) |
| Yes | 631 ( 50.6%) | 403 ( 50.0%) | 181 ( 47.4%) | 33 ( 47.1%) | 77 ( 52.4%) |
| No | 244 ( 19.6%) | 149 ( 18.5%) | 87 ( 22.8%) | 21 ( 30.0%) | 25 ( 17.0%) |
| Missing | 372 ( 29.8%) | 254 ( 31.5%) | 114 ( 29.8%) | 16 ( 22.9%) | 45 ( 30.6%) |

| Table 22.3 - Exercise now (by ISI group) | | | | |
| --- | --- | --- | --- | --- |
| MAS | | | | |
|  | Absence of insomnia (N = 977) | Subthreshold insomnia (N = 642) | Moderate insomnia (N = 314) | Severe insomnia (N = 56) |
| Yes | 511 ( 52.3%) | 307 ( 47.8%) | 141 ( 44.9%) | 30 ( 53.6%) |
| No | 187 ( 19.1%) | 123 ( 19.2%) | 78 ( 24.8%) | 14 ( 25.0%) |
| Missing | 279 ( 28.6%) | 212 ( 33.0%) | 95 ( 30.3%) | 12 ( 21.4%) |

| Table 24.1 - Exercise change (overall and by sex) | | | |
| --- | --- | --- | --- |
| ES | | | |
|  | Total (N = 2652) | Female (N = 2023) | Male (N = 629) |
| Yes, more | 220 ( 8.3%) | 195 ( 9.6%) | 25 ( 4.0%) |
| Yes, unchanged | 576 ( 21.7%) | 429 ( 21.2%) | 147 ( 23.4%) |
| Yes, less | 446 ( 16.8%) | 327 ( 16.2%) | 119 ( 18.9%) |
| Yes, change unknown | 6 ( 0.2%) | 6 ( 0.3%) | 0 ( 0.0%) |
| Started | 76 ( 2.9%) | 71 ( 3.5%) | 5 ( 0.8%) |
| Quit | 494 ( 18.6%) | 356 ( 17.6%) | 138 ( 21.9%) |
| Never | 819 ( 30.9%) | 632 ( 31.2%) | 187 ( 29.7%) |
| Missing | 15 ( 0.6%) | 7 ( 0.3%) | 8 ( 1.3%) |

| Table 24.1 - Exercise change (overall and by sex) | | | |
| --- | --- | --- | --- |
| MAS | | | |
|  | Total (N = 1989) | Female (N = 1515) | Male (N = 474) |
| Yes, more | 169 ( 8.5%) | 150 ( 9.9%) | 19 ( 4.0%) |
| Yes, unchanged | 430 ( 21.6%) | 326 ( 21.5%) | 104 ( 21.9%) |
| Yes, less | 328 ( 16.5%) | 238 ( 15.7%) | 90 ( 19.0%) |
| Yes, change unknown | 4 ( 0.2%) | 4 ( 0.3%) | 0 ( 0.0%) |
| Started | 57 ( 2.9%) | 54 ( 3.6%) | 3 ( 0.6%) |
| Quit | 381 ( 19.2%) | 274 ( 18.1%) | 107 ( 22.6%) |
| Never | 612 ( 30.8%) | 465 ( 30.7%) | 147 ( 31.0%) |
| Missing | 8 ( 0.4%) | 4 ( 0.3%) | 4 ( 0.8%) |

| Table 24.2 - Exercise change (by age) | | | |
| --- | --- | --- | --- |
| ES | | | |
|  | Age 18-30 (N = 892) | Age 31-65 (N = 1662) | Age >65 (N = 98) |
| Yes, more | 90 ( 10.1%) | 126 ( 7.6%) | 4 ( 4.1%) |
| Yes, unchanged | 217 ( 24.3%) | 338 ( 20.3%) | 21 ( 21.4%) |
| Yes, less | 143 ( 16.0%) | 281 ( 16.9%) | 22 ( 22.4%) |
| Yes, change unknown | 2 ( 0.2%) | 4 ( 0.2%) | 0 ( 0.0%) |
| Started | 23 ( 2.6%) | 52 ( 3.1%) | 1 ( 1.0%) |
| Quit | 138 ( 15.5%) | 328 ( 19.7%) | 28 ( 28.6%) |
| Never | 273 ( 30.6%) | 525 ( 31.6%) | 21 ( 21.4%) |
| Missing | 6 ( 0.7%) | 8 ( 0.5%) | 1 ( 1.0%) |

| Table 24.2 - Exercise change (by age) | | | |
| --- | --- | --- | --- |
| MAS | | | |
|  | Age 18-30 (N = 666) | Age 31-65 (N = 1258) | Age >65 (N = 65) |
| Yes, more | 69 ( 10.4%) | 98 ( 7.8%) | 2 ( 3.1%) |
| Yes, unchanged | 158 ( 23.7%) | 259 ( 20.6%) | 13 ( 20.0%) |
| Yes, less | 99 ( 14.9%) | 214 ( 17.0%) | 15 ( 23.1%) |
| Yes, change unknown | 1 ( 0.2%) | 3 ( 0.2%) | 0 ( 0.0%) |
| Started | 17 ( 2.6%) | 39 ( 3.1%) | 1 ( 1.5%) |
| Quit | 110 ( 16.5%) | 250 ( 19.9%) | 21 ( 32.3%) |
| Never | 209 ( 31.4%) | 390 ( 31.0%) | 13 ( 20.0%) |
| Missing | 3 ( 0.5%) | 5 ( 0.4%) | 0 ( 0.0%) |

| Table 24.3 - Exercise change (by ISI group) | | | | | |
| --- | --- | --- | --- | --- | --- |
| ES | | | | | |
|  | Absence of insomnia (N = 1247) | Subthreshold insomnia (N = 806) | Moderate insomnia (N = 382) | Severe insomnia (N = 70) | Unknown (N = 147) |
| Yes, more | 117 ( 9.4%) | 69 ( 8.6%) | 23 ( 6.0%) | 3 ( 4.3%) | 8 ( 5.4%) |
| Yes, unchanged | 283 ( 22.7%) | 170 ( 21.1%) | 72 ( 18.8%) | 17 ( 24.3%) | 34 ( 23.1%) |
| Yes, less | 192 ( 15.4%) | 141 ( 17.5%) | 74 ( 19.4%) | 10 ( 14.3%) | 29 ( 19.7%) |
| Yes, change unknown | 4 ( 0.3%) | 0 ( 0.0%) | 1 ( 0.3%) | 0 ( 0.0%) | 1 ( 0.7%) |
| Started | 35 ( 2.8%) | 22 ( 2.7%) | 11 ( 2.9%) | 3 ( 4.3%) | 5 ( 3.4%) |
| Quit | 231 ( 18.5%) | 140 ( 17.4%) | 81 ( 21.2%) | 19 ( 27.1%) | 23 ( 15.6%) |
| Never | 379 ( 30.4%) | 262 ( 32.5%) | 118 ( 30.9%) | 18 ( 25.7%) | 42 ( 28.6%) |
| Missing | 6 ( 0.5%) | 2 ( 0.2%) | 2 ( 0.5%) | 0 ( 0.0%) | 5 ( 3.4%) |

| Table 24.3 - Exercise change (by ISI group) | | | | |
| --- | --- | --- | --- | --- |
| MAS | | | | |
|  | Absence of insomnia (N = 977) | Subthreshold insomnia (N = 642) | Moderate insomnia (N = 314) | Severe insomnia (N = 56) |
| Yes, more | 97 ( 9.9%) | 52 ( 8.1%) | 17 ( 5.4%) | 3 ( 5.4%) |
| Yes, unchanged | 223 ( 22.8%) | 135 ( 21.0%) | 57 ( 18.2%) | 15 ( 26.8%) |
| Yes, less | 159 ( 16.3%) | 103 ( 16.0%) | 57 ( 18.2%) | 9 ( 16.1%) |
| Yes, change unknown | 3 ( 0.3%) | 0 ( 0.0%) | 1 ( 0.3%) | 0 ( 0.0%) |
| Started | 29 ( 3.0%) | 16 ( 2.5%) | 9 ( 2.9%) | 3 ( 5.4%) |
| Quit | 179 ( 18.3%) | 117 ( 18.2%) | 72 ( 22.9%) | 13 ( 23.2%) |
| Never | 283 ( 29.0%) | 217 ( 33.8%) | 99 ( 31.5%) | 13 ( 23.2%) |
| Missing | 4 ( 0.4%) | 2 ( 0.3%) | 2 ( 0.6%) | 0 ( 0.0%) |

| Table 26.1 - Eating habits (overall and by sex) | | | |
| --- | --- | --- | --- |
| ES | | | |
|  | Total (N = 2652) | Female (N = 2023) | Male (N = 629) |
| Yes, I eat more | 1041 ( 39.3%) | 795 ( 39.3%) | 246 ( 39.1%) |
| Yes, I eat less healthy | 289 ( 10.9%) | 241 ( 11.9%) | 48 ( 7.6%) |
| Yes, I eat less | 211 ( 8.0%) | 160 ( 7.9%) | 51 ( 8.1%) |
| Yes, I eat more healthy | 335 ( 12.6%) | 253 ( 12.5%) | 82 ( 13.0%) |
| No, not changed | 761 ( 28.7%) | 565 ( 27.9%) | 196 ( 31.2%) |
| Missing | 15 ( 0.6%) | 9 ( 0.4%) | 6 ( 1.0%) |

| Table 26.1 - Eating habits (overall and by sex) | | | |
| --- | --- | --- | --- |
| MAS | | | |
|  | Total (N = 1989) | Female (N = 1515) | Male (N = 474) |
| Yes, I eat more | 778 ( 39.1%) | 597 ( 39.4%) | 181 ( 38.2%) |
| Yes, I eat less healthy | 227 ( 11.4%) | 189 ( 12.5%) | 38 ( 8.0%) |
| Yes, I eat less | 158 ( 7.9%) | 118 ( 7.8%) | 40 ( 8.4%) |
| Yes, I eat more healthy | 261 ( 13.1%) | 194 ( 12.8%) | 67 ( 14.1%) |
| No, not changed | 559 ( 28.1%) | 413 ( 27.3%) | 146 ( 30.8%) |
| Missing | 6 ( 0.3%) | 4 ( 0.3%) | 2 ( 0.4%) |

| Table 26.2 - Eating habits (by age) | | | |
| --- | --- | --- | --- |
| ES | | | |
|  | Age 18-30 (N = 892) | Age 31-65 (N = 1662) | Age >65 (N = 98) |
| Yes, I eat more | 344 ( 38.6%) | 674 ( 40.6%) | 23 ( 23.5%) |
| Yes, I eat less healthy | 108 ( 12.1%) | 179 ( 10.8%) | 2 ( 2.0%) |
| Yes, I eat less | 73 ( 8.2%) | 128 ( 7.7%) | 10 ( 10.2%) |
| Yes, I eat more healthy | 99 ( 11.1%) | 220 ( 13.2%) | 16 ( 16.3%) |
| No, not changed | 261 ( 29.3%) | 454 ( 27.3%) | 46 ( 46.9%) |
| Missing | 7 ( 0.8%) | 7 ( 0.4%) | 1 ( 1.0%) |

| Table 26.2 - Eating habits (by age) | | | |
| --- | --- | --- | --- |
| MAS | | | |
|  | Age 18-30 (N = 666) | Age 31-65 (N = 1258) | Age >65 (N = 65) |
| Yes, I eat more | 254 ( 38.1%) | 509 ( 40.5%) | 15 ( 23.1%) |
| Yes, I eat less healthy | 87 ( 13.1%) | 138 ( 11.0%) | 2 ( 3.1%) |
| Yes, I eat less | 51 ( 7.7%) | 101 ( 8.0%) | 6 ( 9.2%) |
| Yes, I eat more healthy | 80 ( 12.0%) | 169 ( 13.4%) | 12 ( 18.5%) |
| No, not changed | 191 ( 28.7%) | 339 ( 26.9%) | 29 ( 44.6%) |
| Missing | 3 ( 0.5%) | 2 ( 0.2%) | 1 ( 1.5%) |

| Table 26.3 - Eating habits (by ISI group) | | | | | |
| --- | --- | --- | --- | --- | --- |
| ES | | | | | |
|  | Absence of insomnia (N = 1247) | Subthreshold insomnia (N = 806) | Moderate insomnia (N = 382) | Severe insomnia (N = 70) | Unknown (N = 147) |
| Yes, I eat more | 435 ( 34.9%) | 350 ( 43.4%) | 165 ( 43.2%) | 33 ( 47.1%) | 58 ( 39.5%) |
| Yes, I eat less healthy | 108 ( 8.7%) | 97 ( 12.0%) | 56 ( 14.7%) | 14 ( 20.0%) | 14 ( 9.5%) |
| Yes, I eat less | 95 ( 7.6%) | 49 ( 6.1%) | 42 ( 11.0%) | 10 ( 14.3%) | 15 ( 10.2%) |
| Yes, I eat more healthy | 189 ( 15.2%) | 97 ( 12.0%) | 36 ( 9.4%) | 4 ( 5.7%) | 9 ( 6.1%) |
| No, not changed | 417 ( 33.4%) | 211 ( 26.2%) | 79 ( 20.7%) | 9 ( 12.9%) | 45 ( 30.6%) |
| Missing | 3 ( 0.2%) | 2 ( 0.2%) | 4 ( 1.0%) | 0 ( 0.0%) | 6 ( 4.1%) |

| Table 26.3 - Eating habits (by ISI group) | | | | |
| --- | --- | --- | --- | --- |
| MAS | | | | |
|  | Absence of insomnia (N = 977) | Subthreshold insomnia (N = 642) | Moderate insomnia (N = 314) | Severe insomnia (N = 56) |
| Yes, I eat more | 349 ( 35.7%) | 271 ( 42.2%) | 132 ( 42.0%) | 26 ( 46.4%) |
| Yes, I eat less healthy | 91 ( 9.3%) | 81 ( 12.6%) | 46 ( 14.6%) | 9 ( 16.1%) |
| Yes, I eat less | 78 ( 8.0%) | 38 ( 5.9%) | 34 ( 10.8%) | 8 ( 14.3%) |
| Yes, I eat more healthy | 145 ( 14.8%) | 82 ( 12.8%) | 30 ( 9.6%) | 4 ( 7.1%) |
| No, not changed | 313 ( 32.0%) | 168 ( 26.2%) | 69 ( 22.0%) | 9 ( 16.1%) |
| Missing | 1 ( 0.1%) | 2 ( 0.3%) | 3 ( 1.0%) | 0 ( 0.0%) |

| Table 27.1 - Alcohol (overall and by sex) | | | |
| --- | --- | --- | --- |
| ES | | | |
|  | Total (N = 2652) | Female (N = 2023) | Male (N = 629) |
| Yes, I drink more liquor | 11 ( 0.4%) | 6 ( 0.3%) | 5 ( 0.8%) |
| Yes, I drink more alcohol | 358 ( 13.5%) | 257 ( 12.7%) | 101 ( 16.1%) |
| Yes, I drink less alcohol | 428 ( 16.1%) | 323 ( 16.0%) | 105 ( 16.7%) |
| No, not changed | 1827 ( 68.9%) | 1418 ( 70.1%) | 409 ( 65.0%) |
| Missing | 28 ( 1.1%) | 19 ( 0.9%) | 9 ( 1.4%) |

| Table 27.1 - Alcohol (overall and by sex) | | | |
| --- | --- | --- | --- |
| MAS | | | |
|  | Total (N = 1989) | Female (N = 1515) | Male (N = 474) |
| Yes, I drink more liquor | 8 ( 0.4%) | 5 ( 0.3%) | 3 ( 0.6%) |
| Yes, I drink more alcohol | 268 ( 13.5%) | 199 ( 13.1%) | 69 ( 14.6%) |
| Yes, I drink less alcohol | 316 ( 15.9%) | 237 ( 15.6%) | 79 ( 16.7%) |
| No, not changed | 1381 ( 69.4%) | 1062 ( 70.1%) | 319 ( 67.3%) |
| Missing | 16 ( 0.8%) | 12 ( 0.8%) | 4 ( 0.8%) |

| Table 27.2 - Alcohol (by age) | | | |
| --- | --- | --- | --- |
| ES | | | |
|  | Age 18-30 (N = 892) | Age 31-65 (N = 1662) | Age >65 (N = 98) |
| Yes, I drink more liquor | 5 ( 0.6%) | 6 ( 0.4%) | 0 ( 0.0%) |
| Yes, I drink more alcohol | 68 ( 7.6%) | 285 ( 17.1%) | 5 ( 5.1%) |
| Yes, I drink less alcohol | 287 ( 32.2%) | 135 ( 8.1%) | 6 ( 6.1%) |
| No, not changed | 520 ( 58.3%) | 1222 ( 73.5%) | 85 ( 86.7%) |
| Missing | 12 ( 1.3%) | 14 ( 0.8%) | 2 ( 2.0%) |

| Table 27.2 - Alcohol (by age) | | | |
| --- | --- | --- | --- |
| MAS | | | |
|  | Age 18-30 (N = 666) | Age 31-65 (N = 1258) | Age >65 (N = 65) |
| Yes, I drink more liquor | 3 ( 0.5%) | 5 ( 0.4%) | 0 ( 0.0%) |
| Yes, I drink more alcohol | 55 ( 8.3%) | 210 ( 16.7%) | 3 ( 4.6%) |
| Yes, I drink less alcohol | 211 ( 31.7%) | 101 ( 8.0%) | 4 ( 6.2%) |
| No, not changed | 390 ( 58.6%) | 934 ( 74.2%) | 57 ( 87.7%) |
| Missing | 7 ( 1.1%) | 8 ( 0.6%) | 1 ( 1.5%) |

| Table 27.3 - Alcohol (by ISI group) | | | | | |
| --- | --- | --- | --- | --- | --- |
| ES | | | | | |
|  | Absence of insomnia (N = 1247) | Subthreshold insomnia (N = 806) | Moderate insomnia (N = 382) | Severe insomnia (N = 70) | Unknown (N = 147) |
| Yes, I drink more liquor | 1 ( 0.1%) | 5 ( 0.6%) | 2 ( 0.5%) | 2 ( 2.9%) | 1 ( 0.7%) |
| Yes, I drink more alcohol | 158 ( 12.7%) | 109 ( 13.5%) | 61 ( 16.0%) | 14 ( 20.0%) | 16 ( 10.9%) |
| Yes, I drink less alcohol | 189 ( 15.2%) | 135 ( 16.7%) | 69 ( 18.1%) | 7 ( 10.0%) | 28 ( 19.0%) |
| No, not changed | 892 ( 71.5%) | 552 ( 68.5%) | 243 ( 63.6%) | 44 ( 62.9%) | 96 ( 65.3%) |
| Missing | 7 ( 0.6%) | 5 ( 0.6%) | 7 ( 1.8%) | 3 ( 4.3%) | 6 ( 4.1%) |

| Table 27.3 - Alcohol (by ISI group) | | | | |
| --- | --- | --- | --- | --- |
| MAS | | | | |
|  | Absence of insomnia (N = 977) | Subthreshold insomnia (N = 642) | Moderate insomnia (N = 314) | Severe insomnia (N = 56) |
| Yes, I drink more liquor | 1 ( 0.1%) | 4 ( 0.6%) | 2 ( 0.6%) | 1 ( 1.8%) |
| Yes, I drink more alcohol | 119 ( 12.2%) | 86 ( 13.4%) | 51 ( 16.2%) | 12 ( 21.4%) |
| Yes, I drink less alcohol | 154 ( 15.8%) | 96 ( 15.0%) | 60 ( 19.1%) | 6 ( 10.7%) |
| No, not changed | 700 ( 71.6%) | 451 ( 70.2%) | 195 ( 62.1%) | 35 ( 62.5%) |
| Missing | 3 ( 0.3%) | 5 ( 0.8%) | 6 ( 1.9%) | 2 ( 3.6%) |

| Table 28.1 - Any past mental disorder (other than insomnia) (overall and by sex) | | | |
| --- | --- | --- | --- |
| ES | | | |
|  | Total (N = 2652) | Female (N = 2023) | Male (N = 629) |
| No | 1895 ( 71.5%) | 1398 ( 69.1%) | 497 ( 79.0%) |
| Yes | 757 ( 28.5%) | 625 ( 30.9%) | 132 ( 21.0%) |

| Table 28.1 - Any past mental disorder (other than insomnia) (overall and by sex) | | | |
| --- | --- | --- | --- |
| MAS | | | |
|  | Total (N = 1989) | Female (N = 1515) | Male (N = 474) |
| No | 1412 ( 71.0%) | 1035 ( 68.3%) | 377 ( 79.5%) |
| Yes | 577 ( 29.0%) | 480 ( 31.7%) | 97 ( 20.5%) |

| Table 28.2 - Any past mental disorder (other than insomnia) (by age) | | | |
| --- | --- | --- | --- |
| ES | | | |
|  | Age 18-30 (N = 892) | Age 31-65 (N = 1662) | Age >65 (N = 98) |
| No | 618 ( 69.3%) | 1200 ( 72.2%) | 77 ( 78.6%) |
| Yes | 274 ( 30.7%) | 462 ( 27.8%) | 21 ( 21.4%) |

| Table 28.2 - Any past mental disorder (other than insomnia) (by age) | | | |
| --- | --- | --- | --- |
| MAS | | | |
|  | Age 18-30 (N = 666) | Age 31-65 (N = 1258) | Age >65 (N = 65) |
| No | 452 ( 67.9%) | 913 ( 72.6%) | 47 ( 72.3%) |
| Yes | 214 ( 32.1%) | 345 ( 27.4%) | 18 ( 27.7%) |

| Table 28.3 - Any past mental disorder (other than insomnia) (by ISI group) | | | | | |
| --- | --- | --- | --- | --- | --- |
| ES | | | | | |
|  | Absence of insomnia (N = 1247) | Subthreshold insomnia (N = 806) | Moderate insomnia (N = 382) | Severe insomnia (N = 70) | Unknown (N = 147) |
| No | 988 ( 79.2%) | 536 ( 66.5%) | 222 ( 58.1%) | 31 ( 44.3%) | 118 ( 80.3%) |
| Yes | 259 ( 20.8%) | 270 ( 33.5%) | 160 ( 41.9%) | 39 ( 55.7%) | 29 ( 19.7%) |

| Table 28.3 - Any past mental disorder (other than insomnia) (by ISI group) | | | | |
| --- | --- | --- | --- | --- |
| MAS | | | | |
|  | Absence of insomnia (N = 977) | Subthreshold insomnia (N = 642) | Moderate insomnia (N = 314) | Severe insomnia (N = 56) |
| No | 780 ( 79.8%) | 432 ( 67.3%) | 175 ( 55.7%) | 25 ( 44.6%) |
| Yes | 197 ( 20.2%) | 210 ( 32.7%) | 139 ( 44.3%) | 31 ( 55.4%) |

| Table 29.1 - Past insomnia (overall and by sex) | | | |
| --- | --- | --- | --- |
| ES | | | |
|  | Total (N = 2652) | Female (N = 2023) | Male (N = 629) |
| No | 2300 ( 86.7%) | 1736 ( 85.8%) | 564 ( 89.7%) |
| Yes | 352 ( 13.3%) | 287 ( 14.2%) | 65 ( 10.3%) |

| Table 29.1 - Past insomnia (overall and by sex) | | | |
| --- | --- | --- | --- |
| MAS | | | |
|  | Total (N = 1989) | Female (N = 1515) | Male (N = 474) |
| No | 1713 ( 86.1%) | 1287 ( 85.0%) | 426 ( 89.9%) |
| Yes | 276 ( 13.9%) | 228 ( 15.0%) | 48 ( 10.1%) |

| Table 29.2 - Past insomnia (by age) | | | |
| --- | --- | --- | --- |
| ES | | | |
|  | Age 18-30 (N = 892) | Age 31-65 (N = 1662) | Age >65 (N = 98) |
| No | 782 ( 87.7%) | 1434 ( 86.3%) | 84 ( 85.7%) |
| Yes | 110 ( 12.3%) | 228 ( 13.7%) | 14 ( 14.3%) |

| Table 29.2 - Past insomnia (by age) | | | |
| --- | --- | --- | --- |
| MAS | | | |
|  | Age 18-30 (N = 666) | Age 31-65 (N = 1258) | Age >65 (N = 65) |
| No | 576 ( 86.5%) | 1080 ( 85.9%) | 57 ( 87.7%) |
| Yes | 90 ( 13.5%) | 178 ( 14.1%) | 8 ( 12.3%) |

| Table 29.3 - Past insomnia (by ISI group) | | | | | |
| --- | --- | --- | --- | --- | --- |
| ES | | | | | |
|  | Absence of insomnia (N = 1247) | Subthreshold insomnia (N = 806) | Moderate insomnia (N = 382) | Severe insomnia (N = 70) | Unknown (N = 147) |
| No | 1183 ( 94.9%) | 671 ( 83.3%) | 274 ( 71.7%) | 40 ( 57.1%) | 132 ( 89.8%) |
| Yes | 64 ( 5.1%) | 135 ( 16.7%) | 108 ( 28.3%) | 30 ( 42.9%) | 15 ( 10.2%) |

| Table 29.3 - Past insomnia (by ISI group) | | | | |
| --- | --- | --- | --- | --- |
| MAS | | | | |
|  | Absence of insomnia (N = 977) | Subthreshold insomnia (N = 642) | Moderate insomnia (N = 314) | Severe insomnia (N = 56) |
| No | 932 ( 95.4%) | 532 ( 82.9%) | 217 ( 69.1%) | 32 ( 57.1%) |
| Yes | 45 ( 4.6%) | 110 ( 17.1%) | 97 ( 30.9%) | 24 ( 42.9%) |

| Table 30.1 - Past mental disorder (overall and by sex) | | | |
| --- | --- | --- | --- |
| ES | | | |
|  | Total (N = 2652) | Female (N = 2023) | Male (N = 629) |
| Insomnia | 352 ( 13.3%) | 287 ( 14.2%) | 65 ( 10.3%) |
| GAD | 307 ( 11.6%) | 247 ( 12.2%) | 60 ( 9.5%) |
| Depression | 299 ( 11.3%) | 249 ( 12.3%) | 50 ( 7.9%) |
| Panic disorder | 269 ( 10.1%) | 228 ( 11.3%) | 41 ( 6.5%) |
| PTSD | 86 ( 3.2%) | 67 ( 3.3%) | 19 ( 3.0%) |
| Anorexia | 69 ( 2.6%) | 66 ( 3.3%) | 3 ( 0.5%) |
| Bulimia | 58 ( 2.2%) | 55 ( 2.7%) | 3 ( 0.5%) |
| OCD | 49 ( 1.8%) | 37 ( 1.8%) | 12 ( 1.9%) |
| Agoraphobia | 27 ( 1.0%) | 22 ( 1.1%) | 5 ( 0.8%) |
| Distimic disorder | 27 ( 1.0%) | 18 ( 0.9%) | 9 ( 1.4%) |
| Post-partum | 26 ( 1.0%) | 26 ( 1.3%) | 0 ( 0.0%) |
| Bipolar disorder | 19 ( 0.7%) | 14 ( 0.7%) | 5 ( 0.8%) |
| Other | 714 ( 26.9%) | 591 ( 29.2%) | 123 ( 19.6%) |

| Table 30.1 - Past mental disorder (overall and by sex) | | | |
| --- | --- | --- | --- |
| MAS | | | |
|  | Total (N = 1989) | Female (N = 1515) | Male (N = 474) |
| Insomnia | 276 ( 13.9%) | 228 ( 15.0%) | 48 ( 10.1%) |
| GAD | 238 ( 12.0%) | 193 ( 12.7%) | 45 ( 9.5%) |
| Depression | 232 ( 11.7%) | 194 ( 12.8%) | 38 ( 8.0%) |
| Panic disorder | 208 ( 10.5%) | 180 ( 11.9%) | 28 ( 5.9%) |
| PTSD | 56 ( 2.8%) | 48 ( 3.2%) | 8 ( 1.7%) |
| Anorexia | 52 ( 2.6%) | 49 ( 3.2%) | 3 ( 0.6%) |
| Bulimia | 44 ( 2.2%) | 41 ( 2.7%) | 3 ( 0.6%) |
| OCD | 40 ( 2.0%) | 30 ( 2.0%) | 10 ( 2.1%) |
| Distimic disorder | 23 ( 1.2%) | 15 ( 1.0%) | 8 ( 1.7%) |
| Agoraphobia | 22 ( 1.1%) | 20 ( 1.3%) | 2 ( 0.4%) |
| Post-partum | 19 ( 1.0%) | 19 ( 1.3%) | 0 ( 0.0%) |
| Bipolar disorder | 11 ( 0.6%) | 8 ( 0.5%) | 3 ( 0.6%) |
| Other | 550 ( 27.7%) | 458 ( 30.2%) | 92 ( 19.4%) |

| Table 30.2 - Past mental disorder (by age) | | | |
| --- | --- | --- | --- |
| ES | | | |
|  | Age 18-30 (N = 892) | Age 31-65 (N = 1662) | Age >65 (N = 98) |
| Insomnia | 110 ( 12.3%) | 228 ( 13.7%) | 14 ( 14.3%) |
| GAD | 132 ( 14.8%) | 168 ( 10.1%) | 7 ( 7.1%) |
| Depression | 112 ( 12.6%) | 179 ( 10.8%) | 8 ( 8.2%) |
| Panic disorder | 115 ( 12.9%) | 153 ( 9.2%) | 1 ( 1.0%) |
| PTSD | 33 ( 3.7%) | 50 ( 3.0%) | 3 ( 3.1%) |
| Anorexia | 32 ( 3.6%) | 37 ( 2.2%) | 0 ( 0.0%) |
| Bulimia | 21 ( 2.4%) | 37 ( 2.2%) | 0 ( 0.0%) |
| OCD | 25 ( 2.8%) | 23 ( 1.4%) | 1 ( 1.0%) |
| Agoraphobia | 14 ( 1.6%) | 13 ( 0.8%) | 0 ( 0.0%) |
| Distimic disorder | 8 ( 0.9%) | 18 ( 1.1%) | 1 ( 1.0%) |
| Post-partum | 3 ( 0.3%) | 23 ( 1.4%) | 0 ( 0.0%) |
| Bipolar disorder | 10 ( 1.1%) | 9 ( 0.5%) | 0 ( 0.0%) |
| Other | 266 ( 29.8%) | 428 ( 25.8%) | 20 ( 20.4%) |

| Table 30.2 - Past mental disorder (by age) | | | |
| --- | --- | --- | --- |
| MAS | | | |
|  | Age 18-30 (N = 666) | Age 31-65 (N = 1258) | Age >65 (N = 65) |
| Insomnia | 90 ( 13.5%) | 178 ( 14.1%) | 8 ( 12.3%) |
| GAD | 102 ( 15.3%) | 131 ( 10.4%) | 5 ( 7.7%) |
| Depression | 91 ( 13.7%) | 134 ( 10.7%) | 7 ( 10.8%) |
| Panic disorder | 97 ( 14.6%) | 111 ( 8.8%) | 0 ( 0.0%) |
| PTSD | 27 ( 4.1%) | 26 ( 2.1%) | 3 ( 4.6%) |
| Anorexia | 23 ( 3.5%) | 29 ( 2.3%) | 0 ( 0.0%) |
| Bulimia | 15 ( 2.3%) | 29 ( 2.3%) | 0 ( 0.0%) |
| OCD | 21 ( 3.2%) | 18 ( 1.4%) | 1 ( 1.5%) |
| Distimic disorder | 7 ( 1.1%) | 15 ( 1.2%) | 1 ( 1.5%) |
| Agoraphobia | 12 ( 1.8%) | 10 ( 0.8%) | 0 ( 0.0%) |
| Post-partum | 3 ( 0.5%) | 16 ( 1.3%) | 0 ( 0.0%) |
| Bipolar disorder | 7 ( 1.1%) | 4 ( 0.3%) | 0 ( 0.0%) |
| Other | 209 ( 31.4%) | 324 ( 25.8%) | 17 ( 26.2%) |

| Table 30.3 - Past mental disorder (by ISI group) | | | | | |
| --- | --- | --- | --- | --- | --- |
| ES | | | | | |
|  | Absence of insomnia (N = 1247) | Subthreshold insomnia (N = 806) | Moderate insomnia (N = 382) | Severe insomnia (N = 70) | Unknown (N = 147) |
| Insomnia | 64 ( 5.1%) | 135 ( 16.7%) | 108 ( 28.3%) | 30 ( 42.9%) | 15 ( 10.2%) |
| GAD | 90 ( 7.2%) | 114 ( 14.1%) | 72 ( 18.8%) | 18 ( 25.7%) | 13 ( 8.8%) |
| Depression | 93 ( 7.5%) | 108 ( 13.4%) | 70 ( 18.3%) | 20 ( 28.6%) | 8 ( 5.4%) |
| Panic disorder | 87 ( 7.0%) | 88 ( 10.9%) | 59 ( 15.4%) | 23 ( 32.9%) | 12 ( 8.2%) |
| PTSD | 19 ( 1.5%) | 29 ( 3.6%) | 20 ( 5.2%) | 10 ( 14.3%) | 8 ( 5.4%) |
| Anorexia | 25 ( 2.0%) | 22 ( 2.7%) | 15 ( 3.9%) | 6 ( 8.6%) | 1 ( 0.7%) |
| Bulimia | 20 ( 1.6%) | 21 ( 2.6%) | 14 ( 3.7%) | 2 ( 2.9%) | 1 ( 0.7%) |
| OCD | 10 ( 0.8%) | 20 ( 2.5%) | 12 ( 3.1%) | 4 ( 5.7%) | 3 ( 2.0%) |
| Agoraphobia | 5 ( 0.4%) | 15 ( 1.9%) | 4 ( 1.0%) | 3 ( 4.3%) | 0 ( 0.0%) |
| Distimic disorder | 13 ( 1.0%) | 7 ( 0.9%) | 6 ( 1.6%) | 0 ( 0.0%) | 1 ( 0.7%) |
| Post-partum | 7 ( 0.6%) | 9 ( 1.1%) | 5 ( 1.3%) | 2 ( 2.9%) | 3 ( 2.0%) |
| Bipolar disorder | 5 ( 0.4%) | 5 ( 0.6%) | 4 ( 1.0%) | 3 ( 4.3%) | 2 ( 1.4%) |
| Other | 246 ( 19.7%) | 257 ( 31.9%) | 152 ( 39.8%) | 37 ( 52.9%) | 22 ( 15.0%) |

| Table 30.3 - Past mental disorder (by ISI group) | | | | |
| --- | --- | --- | --- | --- |
| MAS | | | | |
|  | Absence of insomnia (N = 977) | Subthreshold insomnia (N = 642) | Moderate insomnia (N = 314) | Severe insomnia (N = 56) |
| Insomnia | 45 ( 4.6%) | 110 ( 17.1%) | 97 ( 30.9%) | 24 ( 42.9%) |
| GAD | 75 ( 7.7%) | 87 ( 13.6%) | 61 ( 19.4%) | 15 ( 26.8%) |
| Depression | 63 ( 6.4%) | 90 ( 14.0%) | 64 ( 20.4%) | 15 ( 26.8%) |
| Panic disorder | 63 ( 6.4%) | 68 ( 10.6%) | 57 ( 18.2%) | 20 ( 35.7%) |
| PTSD | 16 ( 1.6%) | 19 ( 3.0%) | 15 ( 4.8%) | 6 ( 10.7%) |
| Anorexia | 19 ( 1.9%) | 16 ( 2.5%) | 12 ( 3.8%) | 5 ( 8.9%) |
| Bulimia | 16 ( 1.6%) | 15 ( 2.3%) | 11 ( 3.5%) | 2 ( 3.6%) |
| OCD | 8 ( 0.8%) | 17 ( 2.6%) | 12 ( 3.8%) | 3 ( 5.4%) |
| Distimic disorder | 12 ( 1.2%) | 5 ( 0.8%) | 6 ( 1.9%) | 0 ( 0.0%) |
| Agoraphobia | 4 ( 0.4%) | 13 ( 2.0%) | 3 ( 1.0%) | 2 ( 3.6%) |
| Post-partum | 6 ( 0.6%) | 8 ( 1.2%) | 3 ( 1.0%) | 2 ( 3.6%) |
| Bipolar disorder | 5 ( 0.5%) | 3 ( 0.5%) | 1 ( 0.3%) | 2 ( 3.6%) |
| Other | 187 ( 19.1%) | 200 ( 31.2%) | 134 ( 42.7%) | 29 ( 51.8%) |

| Table 31.1 - Any current mental disorder (other than insomnia) (overall and by sex) | | | |
| --- | --- | --- | --- |
| ES | | | |
|  | Total (N = 2652) | Female (N = 2023) | Male (N = 629) |
| No | 2272 ( 85.7%) | 1711 ( 84.6%) | 561 ( 89.2%) |
| Yes | 380 ( 14.3%) | 312 ( 15.4%) | 68 ( 10.8%) |

| Table 31.1 - Any current mental disorder (other than insomnia) (overall and by sex) | | | |
| --- | --- | --- | --- |
| MAS | | | |
|  | Total (N = 1989) | Female (N = 1515) | Male (N = 474) |
| No | 1690 ( 85.0%) | 1262 ( 83.3%) | 428 ( 90.3%) |
| Yes | 299 ( 15.0%) | 253 ( 16.7%) | 46 ( 9.7%) |

| Table 31.2 - Any current mental disorder (other than insomnia) (by age) | | | |
| --- | --- | --- | --- |
| ES | | | |
|  | Age 18-30 (N = 892) | Age 31-65 (N = 1662) | Age >65 (N = 98) |
| No | 735 ( 82.4%) | 1451 ( 87.3%) | 86 ( 87.8%) |
| Yes | 157 ( 17.6%) | 211 ( 12.7%) | 12 ( 12.2%) |

| Table 31.2 - Any current mental disorder (other than insomnia) (by age) | | | |
| --- | --- | --- | --- |
| MAS | | | |
|  | Age 18-30 (N = 666) | Age 31-65 (N = 1258) | Age >65 (N = 65) |
| No | 541 ( 81.2%) | 1093 ( 86.9%) | 56 ( 86.2%) |
| Yes | 125 ( 18.8%) | 165 ( 13.1%) | 9 ( 13.8%) |

| Table 31.3 - Any current mental disorder (other than insomnia) (by ISI group) | | | | | |
| --- | --- | --- | --- | --- | --- |
| ES | | | | | |
|  | Absence of insomnia (N = 1247) | Subthreshold insomnia (N = 806) | Moderate insomnia (N = 382) | Severe insomnia (N = 70) | Unknown (N = 147) |
| No | 1186 ( 95.1%) | 667 ( 82.8%) | 260 ( 68.1%) | 31 ( 44.3%) | 128 ( 87.1%) |
| Yes | 61 ( 4.9%) | 139 ( 17.2%) | 122 ( 31.9%) | 39 ( 55.7%) | 19 ( 12.9%) |

| Table 31.3 - Any current mental disorder (other than insomnia) (by ISI group) | | | | |
| --- | --- | --- | --- | --- |
| MAS | | | | |
|  | Absence of insomnia (N = 977) | Subthreshold insomnia (N = 642) | Moderate insomnia (N = 314) | Severe insomnia (N = 56) |
| No | 931 ( 95.3%) | 529 ( 82.4%) | 207 ( 65.9%) | 23 ( 41.1%) |
| Yes | 46 ( 4.7%) | 113 ( 17.6%) | 107 ( 34.1%) | 33 ( 58.9%) |

| Table 32.1 - Current insomnia (overall and by sex) | | | |
| --- | --- | --- | --- |
| ES | | | |
|  | Total (N = 2652) | Female (N = 2023) | Male (N = 629) |
| No | 2403 ( 90.6%) | 1820 ( 90.0%) | 583 ( 92.7%) |
| Yes | 249 ( 9.4%) | 203 ( 10.0%) | 46 ( 7.3%) |

| Table 32.1 - Current insomnia (overall and by sex) | | | |
| --- | --- | --- | --- |
| MAS | | | |
|  | Total (N = 1989) | Female (N = 1515) | Male (N = 474) |
| No | 1792 ( 90.1%) | 1351 ( 89.2%) | 441 ( 93.0%) |
| Yes | 197 ( 9.9%) | 164 ( 10.8%) | 33 ( 7.0%) |

| Table 32.2 - Current insomnia (by age) | | | |
| --- | --- | --- | --- |
| ES | | | |
|  | Age 18-30 (N = 892) | Age 31-65 (N = 1662) | Age >65 (N = 98) |
| No | 812 ( 91.0%) | 1501 ( 90.3%) | 90 ( 91.8%) |
| Yes | 80 ( 9.0%) | 161 ( 9.7%) | 8 ( 8.2%) |

| Table 32.2 - Current insomnia (by age) | | | |
| --- | --- | --- | --- |
| MAS | | | |
|  | Age 18-30 (N = 666) | Age 31-65 (N = 1258) | Age >65 (N = 65) |
| No | 603 ( 90.5%) | 1130 ( 89.8%) | 59 ( 90.8%) |
| Yes | 63 ( 9.5%) | 128 ( 10.2%) | 6 ( 9.2%) |

| Table 32.3 - Current insomnia (by ISI group) | | | | | |
| --- | --- | --- | --- | --- | --- |
| ES | | | | | |
|  | Absence of insomnia (N = 1247) | Subthreshold insomnia (N = 806) | Moderate insomnia (N = 382) | Severe insomnia (N = 70) | Unknown (N = 147) |
| No | 1236 ( 99.1%) | 721 ( 89.5%) | 274 ( 71.7%) | 35 ( 50.0%) | 137 ( 93.2%) |
| Yes | 11 ( 0.9%) | 85 ( 10.5%) | 108 ( 28.3%) | 35 ( 50.0%) | 10 ( 6.8%) |

| Table 32.3 - Current insomnia (by ISI group) | | | | |
| --- | --- | --- | --- | --- |
| MAS | | | | |
|  | Absence of insomnia (N = 977) | Subthreshold insomnia (N = 642) | Moderate insomnia (N = 314) | Severe insomnia (N = 56) |
| No | 970 ( 99.3%) | 574 ( 89.4%) | 221 ( 70.4%) | 27 ( 48.2%) |
| Yes | 7 ( 0.7%) | 68 ( 10.6%) | 93 ( 29.6%) | 29 ( 51.8%) |

| Table 33.1 - Current mental disorder (overall and by sex) | | | |
| --- | --- | --- | --- |
| ES | | | |
|  | Total (N = 2652) | Female (N = 2023) | Male (N = 629) |
| Insomnia | 249 ( 9.4%) | 203 ( 10.0%) | 46 ( 7.3%) |
| GAD | 175 ( 6.6%) | 149 ( 7.4%) | 26 ( 4.1%) |
| Depression | 95 ( 3.6%) | 76 ( 3.8%) | 19 ( 3.0%) |
| Panic disorder | 63 ( 2.4%) | 53 ( 2.6%) | 10 ( 1.6%) |
| PTSD | 25 ( 0.9%) | 20 ( 1.0%) | 5 ( 0.8%) |
| OCD | 24 ( 0.9%) | 17 ( 0.8%) | 7 ( 1.1%) |
| Distimic disorder | 14 ( 0.5%) | 8 ( 0.4%) | 6 ( 1.0%) |
| Bulimia | 7 ( 0.3%) | 6 ( 0.3%) | 1 ( 0.2%) |
| Anorexia | 4 ( 0.2%) | 3 ( 0.1%) | 1 ( 0.2%) |
| Bipolar disorder | 3 ( 0.1%) | 3 ( 0.1%) | 0 ( 0.0%) |
| Other | 341 ( 12.9%) | 280 ( 13.8%) | 61 ( 9.7%) |

| Table 33.1 - Current mental disorder (overall and by sex) | | | |
| --- | --- | --- | --- |
| MAS | | | |
|  | Total (N = 1989) | Female (N = 1515) | Male (N = 474) |
| Insomnia | 197 ( 9.9%) | 164 ( 10.8%) | 33 ( 7.0%) |
| GAD | 131 ( 6.6%) | 116 ( 7.7%) | 15 ( 3.2%) |
| Depression | 73 ( 3.7%) | 62 ( 4.1%) | 11 ( 2.3%) |
| Panic disorder | 51 ( 2.6%) | 45 ( 3.0%) | 6 ( 1.3%) |
| OCD | 19 ( 1.0%) | 15 ( 1.0%) | 4 ( 0.8%) |
| PTSD | 17 ( 0.9%) | 14 ( 0.9%) | 3 ( 0.6%) |
| Distimic disorder | 12 ( 0.6%) | 8 ( 0.5%) | 4 ( 0.8%) |
| Bulimia | 5 ( 0.3%) | 4 ( 0.3%) | 1 ( 0.2%) |
| Anorexia | 4 ( 0.2%) | 3 ( 0.2%) | 1 ( 0.2%) |
| Bipolar disorder | 3 ( 0.2%) | 3 ( 0.2%) | 0 ( 0.0%) |
| Other | 276 ( 13.9%) | 232 ( 15.3%) | 44 ( 9.3%) |

| Table 33.2 - Current mental disorder (by age) | | | |
| --- | --- | --- | --- |
| ES | | | |
|  | Age 18-30 (N = 892) | Age 31-65 (N = 1662) | Age >65 (N = 98) |
| Insomnia | 80 ( 9.0%) | 161 ( 9.7%) | 8 ( 8.2%) |
| GAD | 82 ( 9.2%) | 89 ( 5.4%) | 4 ( 4.1%) |
| Depression | 42 ( 4.7%) | 50 ( 3.0%) | 3 ( 3.1%) |
| Panic disorder | 34 ( 3.8%) | 28 ( 1.7%) | 1 ( 1.0%) |
| PTSD | 15 ( 1.7%) | 9 ( 0.5%) | 1 ( 1.0%) |
| OCD | 14 ( 1.6%) | 10 ( 0.6%) | 0 ( 0.0%) |
| Distimic disorder | 7 ( 0.8%) | 7 ( 0.4%) | 0 ( 0.0%) |
| Bulimia | 5 ( 0.6%) | 2 ( 0.1%) | 0 ( 0.0%) |
| Anorexia | 3 ( 0.3%) | 1 ( 0.1%) | 0 ( 0.0%) |
| Bipolar disorder | 2 ( 0.2%) | 1 ( 0.1%) | 0 ( 0.0%) |
| Other | 142 ( 15.9%) | 190 ( 11.4%) | 9 ( 9.2%) |

| Table 33.2 - Current mental disorder (by age) | | | |
| --- | --- | --- | --- |
| MAS | | | |
|  | Age 18-30 (N = 666) | Age 31-65 (N = 1258) | Age >65 (N = 65) |
| Insomnia | 63 ( 9.5%) | 128 ( 10.2%) | 6 ( 9.2%) |
| GAD | 60 ( 9.0%) | 69 ( 5.5%) | 2 ( 3.1%) |
| Depression | 35 ( 5.3%) | 36 ( 2.9%) | 2 ( 3.1%) |
| Panic disorder | 29 ( 4.4%) | 21 ( 1.7%) | 1 ( 1.5%) |
| OCD | 10 ( 1.5%) | 9 ( 0.7%) | 0 ( 0.0%) |
| PTSD | 12 ( 1.8%) | 5 ( 0.4%) | 0 ( 0.0%) |
| Distimic disorder | 6 ( 0.9%) | 6 ( 0.5%) | 0 ( 0.0%) |
| Bulimia | 3 ( 0.5%) | 2 ( 0.2%) | 0 ( 0.0%) |
| Anorexia | 3 ( 0.5%) | 1 ( 0.1%) | 0 ( 0.0%) |
| Bipolar disorder | 2 ( 0.3%) | 1 ( 0.1%) | 0 ( 0.0%) |
| Other | 116 ( 17.4%) | 152 ( 12.1%) | 8 ( 12.3%) |

| Table 33.3 - Current mental disorder (by ISI group) | | | | | |
| --- | --- | --- | --- | --- | --- |
| ES | | | | | |
|  | Absence of insomnia (N = 1247) | Subthreshold insomnia (N = 806) | Moderate insomnia (N = 382) | Severe insomnia (N = 70) | Unknown (N = 147) |
| Insomnia | 11 ( 0.9%) | 85 ( 10.5%) | 108 ( 28.3%) | 35 ( 50.0%) | 10 ( 6.8%) |
| GAD | 32 ( 2.6%) | 62 ( 7.7%) | 53 ( 13.9%) | 16 ( 22.9%) | 12 ( 8.2%) |
| Depression | 14 ( 1.1%) | 32 ( 4.0%) | 33 ( 8.6%) | 15 ( 21.4%) | 1 ( 0.7%) |
| Panic disorder | 11 ( 0.9%) | 17 ( 2.1%) | 18 ( 4.7%) | 12 ( 17.1%) | 5 ( 3.4%) |
| PTSD | 6 ( 0.5%) | 9 ( 1.1%) | 3 ( 0.8%) | 6 ( 8.6%) | 1 ( 0.7%) |
| OCD | 5 ( 0.4%) | 11 ( 1.4%) | 5 ( 1.3%) | 3 ( 4.3%) | 0 ( 0.0%) |
| Distimic disorder | 6 ( 0.5%) | 3 ( 0.4%) | 5 ( 1.3%) | 0 ( 0.0%) | 0 ( 0.0%) |
| Bulimia | 3 ( 0.2%) | 1 ( 0.1%) | 2 ( 0.5%) | 1 ( 1.4%) | 0 ( 0.0%) |
| Anorexia | 1 ( 0.1%) | 1 ( 0.1%) | 1 ( 0.3%) | 1 ( 1.4%) | 0 ( 0.0%) |
| Bipolar disorder | 0 ( 0.0%) | 2 ( 0.2%) | 0 ( 0.0%) | 1 ( 1.4%) | 0 ( 0.0%) |
| Other | 51 ( 4.1%) | 122 ( 15.1%) | 117 ( 30.6%) | 37 ( 52.9%) | 14 ( 9.5%) |

| Table 33.3 - Current mental disorder (by ISI group) | | | | |
| --- | --- | --- | --- | --- |
| MAS | | | | |
|  | Absence of insomnia (N = 977) | Subthreshold insomnia (N = 642) | Moderate insomnia (N = 314) | Severe insomnia (N = 56) |
| Insomnia | 7 ( 0.7%) | 68 ( 10.6%) | 93 ( 29.6%) | 29 ( 51.8%) |
| GAD | 25 ( 2.6%) | 49 ( 7.6%) | 45 ( 14.3%) | 12 ( 21.4%) |
| Depression | 9 ( 0.9%) | 21 ( 3.3%) | 31 ( 9.9%) | 12 ( 21.4%) |
| Panic disorder | 9 ( 0.9%) | 16 ( 2.5%) | 17 ( 5.4%) | 9 ( 16.1%) |
| OCD | 3 ( 0.3%) | 9 ( 1.4%) | 5 ( 1.6%) | 2 ( 3.6%) |
| PTSD | 4 ( 0.4%) | 7 ( 1.1%) | 2 ( 0.6%) | 4 ( 7.1%) |
| Distimic disorder | 6 ( 0.6%) | 2 ( 0.3%) | 4 ( 1.3%) | 0 ( 0.0%) |
| Bulimia | 2 ( 0.2%) | 0 ( 0.0%) | 2 ( 0.6%) | 1 ( 1.8%) |
| Anorexia | 1 ( 0.1%) | 1 ( 0.2%) | 1 ( 0.3%) | 1 ( 1.8%) |
| Bipolar disorder | 0 ( 0.0%) | 2 ( 0.3%) | 0 ( 0.0%) | 1 ( 1.8%) |
| Other | 39 ( 4.0%) | 103 ( 16.0%) | 102 ( 32.5%) | 32 ( 57.1%) |

| Table 34.1 - Any comorbidity (overall and by sex) | | | |
| --- | --- | --- | --- |
| ES | | | |
|  | Total (N = 2652) | Female (N = 2023) | Male (N = 629) |
| No | 2028 ( 76.5%) | 1549 ( 76.6%) | 479 ( 76.2%) |
| Yes | 550 ( 20.7%) | 422 ( 20.9%) | 128 ( 20.3%) |

| Table 34.1 - Any comorbidity (overall and by sex) | | | |
| --- | --- | --- | --- |
| MAS | | | |
|  | Total (N = 1989) | Female (N = 1515) | Male (N = 474) |
| No | 1514 ( 76.1%) | 1156 ( 76.3%) | 358 ( 75.5%) |
| Yes | 432 ( 21.7%) | 329 ( 21.7%) | 103 ( 21.7%) |

| Table 34.2 - Any comorbidity (by age) | | | |
| --- | --- | --- | --- |
| ES | | | |
|  | Age 18-30 (N = 892) | Age 31-65 (N = 1662) | Age >65 (N = 98) |
| No | 728 ( 81.6%) | 1249 ( 75.2%) | 51 ( 52.0%) |
| Yes | 130 ( 14.6%) | 378 ( 22.7%) | 42 ( 42.9%) |

| Table 34.2 - Any comorbidity (by age) | | | |
| --- | --- | --- | --- |
| MAS | | | |
|  | Age 18-30 (N = 666) | Age 31-65 (N = 1258) | Age >65 (N = 65) |
| No | 534 ( 80.2%) | 945 ( 75.1%) | 35 ( 53.8%) |
| Yes | 111 ( 16.7%) | 294 ( 23.4%) | 27 ( 41.5%) |

| Table 34.3 - Any comorbidity (by ISI group) | | | | | |
| --- | --- | --- | --- | --- | --- |
| ES | | | | | |
|  | Absence of insomnia (N = 1247) | Subthreshold insomnia (N = 806) | Moderate insomnia (N = 382) | Severe insomnia (N = 70) | Unknown (N = 147) |
| No | 983 ( 78.8%) | 624 ( 77.4%) | 265 ( 69.4%) | 47 ( 67.1%) | 109 ( 74.1%) |
| Yes | 236 ( 18.9%) | 162 ( 20.1%) | 103 ( 27.0%) | 22 ( 31.4%) | 27 ( 18.4%) |

| Table 34.3 - Any comorbidity (by ISI group) | | | | |
| --- | --- | --- | --- | --- |
| MAS | | | | |
|  | Absence of insomnia (N = 977) | Subthreshold insomnia (N = 642) | Moderate insomnia (N = 314) | Severe insomnia (N = 56) |
| No | 768 ( 78.6%) | 497 ( 77.4%) | 210 ( 66.9%) | 39 ( 69.6%) |
| Yes | 193 ( 19.8%) | 132 ( 20.6%) | 90 ( 28.7%) | 17 ( 30.4%) |

| Table 35.1 - Any drugs (overall and by sex) | | | |
| --- | --- | --- | --- |
| ES | | | |
|  | Total (N = 2652) | Female (N = 2023) | Male (N = 629) |
| No | 1868 ( 70.4%) | 1411 ( 69.7%) | 457 ( 72.7%) |
| Yes | 709 ( 26.7%) | 550 ( 27.2%) | 159 ( 25.3%) |

| Table 35.1 - Any drugs (overall and by sex) | | | |
| --- | --- | --- | --- |
| MAS | | | |
|  | Total (N = 1989) | Female (N = 1515) | Male (N = 474) |
| No | 1386 ( 69.7%) | 1046 ( 69.0%) | 340 ( 71.7%) |
| Yes | 566 ( 28.5%) | 438 ( 28.9%) | 128 ( 27.0%) |

| Table 35.2 - Any drugs (by age) | | | |
| --- | --- | --- | --- |
| ES | | | |
|  | Age 18-30 (N = 892) | Age 31-65 (N = 1662) | Age >65 (N = 98) |
| No | 690 ( 77.4%) | 1149 ( 69.1%) | 29 ( 29.6%) |
| Yes | 172 ( 19.3%) | 472 ( 28.4%) | 65 ( 66.3%) |

| Table 35.2 - Any drugs (by age) | | | |
| --- | --- | --- | --- |
| MAS | | | |
|  | Age 18-30 (N = 666) | Age 31-65 (N = 1258) | Age >65 (N = 65) |
| No | 506 ( 76.0%) | 860 ( 68.4%) | 20 ( 30.8%) |
| Yes | 142 ( 21.3%) | 383 ( 30.4%) | 41 ( 63.1%) |

| Table 35.3 - Any drugs (by ISI group) | | | | | |
| --- | --- | --- | --- | --- | --- |
| ES | | | | | |
|  | Absence of insomnia (N = 1247) | Subthreshold insomnia (N = 806) | Moderate insomnia (N = 382) | Severe insomnia (N = 70) | Unknown (N = 147) |
| No | 897 ( 71.9%) | 573 ( 71.1%) | 252 ( 66.0%) | 49 ( 70.0%) | 97 ( 66.0%) |
| Yes | 325 ( 26.1%) | 210 ( 26.1%) | 119 ( 31.2%) | 19 ( 27.1%) | 36 ( 24.5%) |

| Table 35.3 - Any drugs (by ISI group) | | | | |
| --- | --- | --- | --- | --- |
| MAS | | | | |
|  | Absence of insomnia (N = 977) | Subthreshold insomnia (N = 642) | Moderate insomnia (N = 314) | Severe insomnia (N = 56) |
| No | 702 ( 71.9%) | 450 ( 70.1%) | 195 ( 62.1%) | 39 ( 69.6%) |
| Yes | 265 ( 27.1%) | 176 ( 27.4%) | 109 ( 34.7%) | 16 ( 28.6%) |

| Table 37.1 - Worrying about COVID (overall and by sex) | | | |
| --- | --- | --- | --- |
| ES | | | |
|  | Total (N = 2652) | Female (N = 2023) | Male (N = 629) |
| n | 2637 | 2013 | 624 |
| Mean | 68.1 | 69.8 | 62.5 |
| StD | 23.55 | 22.74 | 25.24 |
| Median | 71.0 | 73.0 | 70.0 |
| 25%/75% quantile | 51.0/85.0 | 55.0/86.0 | 50.0/80.0 |
| Min/Max | 0/100 | 0/100 | 0/100 |

| Table 37.1 - Worrying about COVID (overall and by sex) | | | |
| --- | --- | --- | --- |
| MAS | | | |
|  | Total (N = 1989) | Female (N = 1515) | Male (N = 474) |
| n | 1983 | 1511 | 472 |
| Mean | 68.0 | 69.9 | 61.6 |
| StD | 23.22 | 22.23 | 25.16 |
| Median | 71.0 | 72.0 | 66.0 |
| 25%/75% quantile | 50.0/84.0 | 55.0/85.0 | 50.0/80.0 |
| Min/Max | 0/100 | 0/100 | 0/100 |

| Table 37.2 - Worrying about COVID (by age) | | | |
| --- | --- | --- | --- |
| ES | | | |
|  | Age 18-30 (N = 892) | Age 31-65 (N = 1662) | Age >65 (N = 98) |
| n | 888 | 1652 | 97 |
| Mean | 66.6 | 68.7 | 70.5 |
| StD | 23.88 | 23.36 | 23.44 |
| Median | 70.0 | 71.5 | 79.0 |
| 25%/75% quantile | 50.0/82.0 | 52.0/85.0 | 55.0/89.0 |
| Min/Max | 0/100 | 0/100 | 0/100 |

| Table 37.2 - Worrying about COVID (by age) | | | |
| --- | --- | --- | --- |
| MAS | | | |
|  | Age 18-30 (N = 666) | Age 31-65 (N = 1258) | Age >65 (N = 65) |
| n | 665 | 1254 | 64 |
| Mean | 66.3 | 68.7 | 71.2 |
| StD | 23.12 | 23.27 | 22.66 |
| Median | 70.0 | 71.0 | 79.5 |
| 25%/75% quantile | 50.0/81.0 | 51.2/85.0 | 54.2/87.5 |
| Min/Max | 0/100 | 0/100 | 8/100 |

| Table 37.3 - Worrying about COVID (by ISI group) | | | | | |
| --- | --- | --- | --- | --- | --- |
| ES | | | | | |
|  | Absence of insomnia (N = 1247) | Subthreshold insomnia (N = 806) | Moderate insomnia (N = 382) | Severe insomnia (N = 70) | Unknown (N = 147) |
| n | 1243 | 803 | 381 | 70 | 140 |
| Mean | 64.0 | 69.7 | 75.0 | 79.6 | 70.5 |
| StD | 23.25 | 22.75 | 24.18 | 22.96 | 21.79 |
| Median | 70.0 | 73.0 | 80.0 | 85.0 | 75.0 |
| 25%/75% quantile | 50.0/ 80.0 | 55.0/ 85.0 | 63.0/ 95.0 | 69.2/100.0 | 59.0/ 85.2 |
| Min/Max | 0/100 | 0/100 | 0/100 | 9/100 | 3/100 |

| Table 37.3 - Worrying about COVID (by ISI group) | | | | |
| --- | --- | --- | --- | --- |
| MAS | | | | |
|  | Absence of insomnia (N = 977) | Subthreshold insomnia (N = 642) | Moderate insomnia (N = 314) | Severe insomnia (N = 56) |
| n | 975 | 639 | 313 | 56 |
| Mean | 63.9 | 69.7 | 74.7 | 82.1 |
| StD | 22.87 | 21.86 | 24.39 | 21.28 |
| Median | 70.0 | 73.0 | 80.0 | 89.0 |
| 25%/75% quantile | 50.0/ 80.0 | 55.0/ 85.0 | 63.0/ 95.0 | 70.8/100.0 |
| Min/Max | 0/100 | 0/100 | 0/100 | 9/100 |

| Table 38.1 - Worry to be infected (overall and by sex) | | | |
| --- | --- | --- | --- |
| ES | | | |
|  | Total (N = 2652) | Female (N = 2023) | Male (N = 629) |
| n | 2606 | 1990 | 616 |
| Mean | 42.5 | 43.3 | 39.8 |
| StD | 27.07 | 26.99 | 27.17 |
| Median | 41.0 | 44.0 | 40.0 |
| 25%/75% quantile | 20.0/60.0 | 20.0/60.0 | 20.0/57.2 |
| Min/Max | 0/100 | 0/100 | 0/100 |

| Table 38.1 - Worry to be infected (overall and by sex) | | | |
| --- | --- | --- | --- |
| MAS | | | |
|  | Total (N = 1989) | Female (N = 1515) | Male (N = 474) |
| n | 1972 | 1503 | 469 |
| Mean | 42.5 | 43.6 | 39.0 |
| StD | 27.07 | 27.09 | 26.73 |
| Median | 41.0 | 45.0 | 36.0 |
| 25%/75% quantile | 20.0/60.0 | 20.0/60.0 | 20.0/56.0 |
| Min/Max | 0/100 | 0/100 | 0/100 |

| Table 38.2 - Worry to be infected (by age) | | | |
| --- | --- | --- | --- |
| ES | | | |
|  | Age 18-30 (N = 892) | Age 31-65 (N = 1662) | Age >65 (N = 98) |
| n | 877 | 1633 | 96 |
| Mean | 39.5 | 44.1 | 41.3 |
| StD | 27.24 | 26.88 | 26.69 |
| Median | 36.0 | 47.0 | 43.0 |
| 25%/75% quantile | 19.0/58.0 | 22.0/60.0 | 20.0/60.0 |
| Min/Max | 0/100 | 0/100 | 0/100 |

| Table 38.2 - Worry to be infected (by age) | | | |
| --- | --- | --- | --- |
| MAS | | | |
|  | Age 18-30 (N = 666) | Age 31-65 (N = 1258) | Age >65 (N = 65) |
| n | 661 | 1246 | 65 |
| Mean | 39.7 | 44.1 | 42.1 |
| StD | 27.11 | 27.01 | 25.87 |
| Median | 36.0 | 47.0 | 44.0 |
| 25%/75% quantile | 19.0/60.0 | 21.0/60.0 | 20.0/55.0 |
| Min/Max | 0/100 | 0/100 | 0/100 |

| Table 38.3 - Worry to be infected (by ISI group) | | | | | |
| --- | --- | --- | --- | --- | --- |
| ES | | | | | |
|  | Absence of insomnia (N = 1247) | Subthreshold insomnia (N = 806) | Moderate insomnia (N = 382) | Severe insomnia (N = 70) | Unknown (N = 147) |
| n | 1231 | 793 | 377 | 69 | 136 |
| Mean | 39.1 | 44.9 | 47.7 | 48.9 | 41.5 |
| StD | 25.81 | 26.41 | 30.63 | 29.48 | 26.36 |
| Median | 38.0 | 49.0 | 50.0 | 50.0 | 40.0 |
| 25%/75% quantile | 20.0/52.0 | 25.0/60.0 | 20.0/74.0 | 20.0/70.0 | 21.0/58.5 |
| Min/Max | 0/100 | 0/100 | 0/100 | 5/100 | 0/100 |

| Table 38.3 - Worry to be infected (by ISI group) | | | | |
| --- | --- | --- | --- | --- |
| MAS | | | | |
|  | Absence of insomnia (N = 977) | Subthreshold insomnia (N = 642) | Moderate insomnia (N = 314) | Severe insomnia (N = 56) |
| n | 970 | 635 | 311 | 56 |
| Mean | 38.9 | 45.4 | 47.1 | 48.6 |
| StD | 25.35 | 26.63 | 30.70 | 31.18 |
| Median | 37.0 | 50.0 | 50.0 | 50.0 |
| 25%/75% quantile | 20.0/52.0 | 25.0/61.5 | 20.0/73.5 | 19.8/77.0 |
| Min/Max | 0/100 | 0/100 | 0/100 | 5/100 |

| Table 39.1 - Infected (overall and by sex) | | | |
| --- | --- | --- | --- |
| ES | | | |
|  | Total (N = 2652) | Female (N = 2023) | Male (N = 629) |
| Yes | 43 ( 1.6%) | 33 ( 1.6%) | 10 ( 1.6%) |
| No | 2587 ( 97.5%) | 1972 ( 97.5%) | 615 ( 97.8%) |
| Missing | 22 ( 0.8%) | 18 ( 0.9%) | 4 ( 0.6%) |

| Table 39.1 - Infected (overall and by sex) | | | |
| --- | --- | --- | --- |
| MAS | | | |
|  | Total (N = 1989) | Female (N = 1515) | Male (N = 474) |
| Yes | 26 ( 1.3%) | 23 ( 1.5%) | 3 ( 0.6%) |
| No | 1955 ( 98.3%) | 1485 ( 98.0%) | 470 ( 99.2%) |
| Missing | 8 ( 0.4%) | 7 ( 0.5%) | 1 ( 0.2%) |

| Table 39.2 - Infected (by age) | | | |
| --- | --- | --- | --- |
| ES | | | |
|  | Age 18-30 (N = 892) | Age 31-65 (N = 1662) | Age >65 (N = 98) |
| Yes | 12 ( 1.3%) | 28 ( 1.7%) | 3 ( 3.1%) |
| No | 873 ( 97.9%) | 1620 ( 97.5%) | 94 ( 95.9%) |
| Missing | 7 ( 0.8%) | 14 ( 0.8%) | 1 ( 1.0%) |

| Table 39.2 - Infected (by age) | | | |
| --- | --- | --- | --- |
| MAS | | | |
|  | Age 18-30 (N = 666) | Age 31-65 (N = 1258) | Age >65 (N = 65) |
| Yes | 8 ( 1.2%) | 15 ( 1.2%) | 3 ( 4.6%) |
| No | 655 ( 98.3%) | 1238 ( 98.4%) | 62 ( 95.4%) |
| Missing | 3 ( 0.5%) | 5 ( 0.4%) | 0 ( 0.0%) |

| Table 39.3 - Infected (by ISI group) | | | | | |
| --- | --- | --- | --- | --- | --- |
| ES | | | | | |
|  | Absence of insomnia (N = 1247) | Subthreshold insomnia (N = 806) | Moderate insomnia (N = 382) | Severe insomnia (N = 70) | Unknown (N = 147) |
| Yes | 19 ( 1.5%) | 11 ( 1.4%) | 8 ( 2.1%) | 2 ( 2.9%) | 3 ( 2.0%) |
| No | 1222 ( 98.0%) | 788 ( 97.8%) | 373 ( 97.6%) | 68 ( 97.1%) | 136 ( 92.5%) |
| Missing | 6 ( 0.5%) | 7 ( 0.9%) | 1 ( 0.3%) | 0 ( 0.0%) | 8 ( 5.4%) |

| Table 39.3 - Infected (by ISI group) | | | | |
| --- | --- | --- | --- | --- |
| MAS | | | | |
|  | Absence of insomnia (N = 977) | Subthreshold insomnia (N = 642) | Moderate insomnia (N = 314) | Severe insomnia (N = 56) |
| Yes | 11 ( 1.1%) | 10 ( 1.6%) | 5 ( 1.6%) | 0 ( 0.0%) |
| No | 964 ( 98.7%) | 627 ( 97.7%) | 308 ( 98.1%) | 56 (100.0%) |
| Missing | 2 ( 0.2%) | 5 ( 0.8%) | 1 ( 0.3%) | 0 ( 0.0%) |

| Table 40.1 - Lost someone (overall and by sex) | | | |
| --- | --- | --- | --- |
| ES | | | |
|  | Total (N = 2652) | Female (N = 2023) | Male (N = 629) |
| Yes | 172 ( 6.5%) | 134 ( 6.6%) | 38 ( 6.0%) |
| No | 2462 ( 92.8%) | 1877 ( 92.8%) | 585 ( 93.0%) |
| Missing | 18 ( 0.7%) | 12 ( 0.6%) | 6 ( 1.0%) |

| Table 40.1 - Lost someone (overall and by sex) | | | |
| --- | --- | --- | --- |
| MAS | | | |
|  | Total (N = 1989) | Female (N = 1515) | Male (N = 474) |
| Yes | 133 ( 6.7%) | 102 ( 6.7%) | 31 ( 6.5%) |
| No | 1850 ( 93.0%) | 1409 ( 93.0%) | 441 ( 93.0%) |
| Missing | 6 ( 0.3%) | 4 ( 0.3%) | 2 ( 0.4%) |

| Table 40.2 - Lost someone (by age) | | | |
| --- | --- | --- | --- |
| ES | | | |
|  | Age 18-30 (N = 892) | Age 31-65 (N = 1662) | Age >65 (N = 98) |
| Yes | 49 ( 5.5%) | 122 ( 7.3%) | 1 ( 1.0%) |
| No | 837 ( 93.8%) | 1528 ( 91.9%) | 97 ( 99.0%) |
| Missing | 6 ( 0.7%) | 12 ( 0.7%) | 0 ( 0.0%) |

| Table 40.2 - Lost someone (by age) | | | |
| --- | --- | --- | --- |
| MAS | | | |
|  | Age 18-30 (N = 666) | Age 31-65 (N = 1258) | Age >65 (N = 65) |
| Yes | 38 ( 5.7%) | 95 ( 7.6%) | 0 ( 0.0%) |
| No | 626 ( 94.0%) | 1159 ( 92.1%) | 65 (100.0%) |
| Missing | 2 ( 0.3%) | 4 ( 0.3%) | 0 ( 0.0%) |

| Table 40.3 - Lost someone (by ISI group) | | | | | |
| --- | --- | --- | --- | --- | --- |
| ES | | | | | |
|  | Absence of insomnia (N = 1247) | Subthreshold insomnia (N = 806) | Moderate insomnia (N = 382) | Severe insomnia (N = 70) | Unknown (N = 147) |
| Yes | 73 ( 5.9%) | 59 ( 7.3%) | 24 ( 6.3%) | 8 ( 11.4%) | 8 ( 5.4%) |
| No | 1171 ( 93.9%) | 740 ( 91.8%) | 357 ( 93.5%) | 62 ( 88.6%) | 132 ( 89.8%) |
| Missing | 3 ( 0.2%) | 7 ( 0.9%) | 1 ( 0.3%) | 0 ( 0.0%) | 7 ( 4.8%) |

| Table 40.3 - Lost someone (by ISI group) | | | | |
| --- | --- | --- | --- | --- |
| MAS | | | | |
|  | Absence of insomnia (N = 977) | Subthreshold insomnia (N = 642) | Moderate insomnia (N = 314) | Severe insomnia (N = 56) |
| Yes | 60 ( 6.1%) | 47 ( 7.3%) | 20 ( 6.4%) | 6 ( 10.7%) |
| No | 915 ( 93.7%) | 592 ( 92.2%) | 293 ( 93.3%) | 50 ( 89.3%) |
| Missing | 2 ( 0.2%) | 3 ( 0.5%) | 1 ( 0.3%) | 0 ( 0.0%) |

| Table 41.1 - Worry someone else gets infected (overall and by sex) | | | |
| --- | --- | --- | --- |
| ES | | | |
|  | Total (N = 2652) | Female (N = 2023) | Male (N = 629) |
| n | 2631 | 2009 | 622 |
| Mean | 68.0 | 69.8 | 62.2 |
| StD | 27.99 | 27.51 | 28.75 |
| Median | 73.0 | 76.0 | 65.0 |
| 25%/75% quantile | 50.0/93.0 | 50.0/97.0 | 47.0/86.0 |
| Min/Max | 0/100 | 0/100 | 0/100 |

| Table 41.1 - Worry someone else gets infected (overall and by sex) | | | |
| --- | --- | --- | --- |
| MAS | | | |
|  | Total (N = 1989) | Female (N = 1515) | Male (N = 474) |
| n | 1979 | 1507 | 472 |
| Mean | 68.0 | 70.0 | 61.7 |
| StD | 28.13 | 27.75 | 28.41 |
| Median | 73.0 | 78.0 | 64.0 |
| 25%/75% quantile | 50.0/94.0 | 50.0/98.0 | 44.5/85.0 |
| Min/Max | 0/100 | 0/100 | 0/100 |

| Table 41.2 - Worry someone else gets infected (by age) | | | |
| --- | --- | --- | --- |
| ES | | | |
|  | Age 18-30 (N = 892) | Age 31-65 (N = 1662) | Age >65 (N = 98) |
| n | 886 | 1647 | 98 |
| Mean | 70.5 | 67.0 | 61.2 |
| StD | 27.79 | 27.91 | 29.32 |
| Median | 78.0 | 71.0 | 61.0 |
| 25%/75% quantile | 50.0/100.0 | 50.0/ 91.0 | 42.2/ 86.8 |
| Min/Max | 0/100 | 0/100 | 0/100 |

| Table 41.2 - Worry someone else gets infected (by age) | | | |
| --- | --- | --- | --- |
| MAS | | | |
|  | Age 18-30 (N = 666) | Age 31-65 (N = 1258) | Age >65 (N = 65) |
| n | 664 | 1250 | 65 |
| Mean | 70.5 | 67.1 | 60.0 |
| StD | 27.77 | 28.19 | 28.53 |
| Median | 78.0 | 71.0 | 54.0 |
| 25%/75% quantile | 50.0/100.0 | 50.0/ 92.0 | 42.0/ 86.0 |
| Min/Max | 0/100 | 0/100 | 0/100 |

| Table 41.3 - Worry someone else gets infected (by ISI group) | | | | | |
| --- | --- | --- | --- | --- | --- |
| ES | | | | | |
|  | Absence of insomnia (N = 1247) | Subthreshold insomnia (N = 806) | Moderate insomnia (N = 382) | Severe insomnia (N = 70) | Unknown (N = 147) |
| n | 1243 | 798 | 380 | 70 | 140 |
| Mean | 62.9 | 71.1 | 75.3 | 84.1 | 67.4 |
| StD | 27.66 | 26.94 | 28.26 | 25.33 | 27.69 |
| Median | 67.0 | 78.5 | 85.0 | 100.0 | 71.5 |
| 25%/75% quantile | 47.0/ 85.0 | 51.0/ 98.0 | 60.0/100.0 | 79.2/100.0 | 50.0/ 91.2 |
| Min/Max | 0/100 | 0/100 | 0/100 | 7/100 | 2/100 |

| Table 41.3 - Worry someone else gets infected (by ISI group) | | | | |
| --- | --- | --- | --- | --- |
| MAS | | | | |
|  | Absence of insomnia (N = 977) | Subthreshold insomnia (N = 642) | Moderate insomnia (N = 314) | Severe insomnia (N = 56) |
| n | 974 | 637 | 312 | 56 |
| Mean | 62.8 | 71.3 | 74.5 | 86.2 |
| StD | 27.63 | 27.29 | 28.63 | 22.61 |
| Median | 67.0 | 79.0 | 85.0 | 100.0 |
| 25%/75% quantile | 45.0/ 85.0 | 51.0/100.0 | 54.0/100.0 | 80.0/100.0 |
| Min/Max | 0/100 | 0/100 | 0/100 | 7/100 |

| Table 42.1 - ISI categories (overall and by sex) | | | |
| --- | --- | --- | --- |
| ES | | | |
|  | Total (N = 2652) | Female (N = 2023) | Male (N = 629) |
| Absence of insomnia | 1247 ( 47.0%) | 911 ( 45.0%) | 336 ( 53.4%) |
| Subthreshold insomnia | 806 ( 30.4%) | 648 ( 32.0%) | 158 ( 25.1%) |
| Moderate insomnia | 382 ( 14.4%) | 293 ( 14.5%) | 89 ( 14.1%) |
| Severe insomnia | 70 ( 2.6%) | 55 ( 2.7%) | 15 ( 2.4%) |
| Missing | 147 ( 5.5%) | 116 ( 5.7%) | 31 ( 4.9%) |

| Table 42.1 - ISI categories (overall and by sex) | | | |
| --- | --- | --- | --- |
| MAS | | | |
|  | Total (N = 1989) | Female (N = 1515) | Male (N = 474) |
| Absence of insomnia | 977 ( 49.1%) | 712 ( 47.0%) | 265 ( 55.9%) |
| Subthreshold insomnia | 642 ( 32.3%) | 515 ( 34.0%) | 127 ( 26.8%) |
| Moderate insomnia | 314 ( 15.8%) | 242 ( 16.0%) | 72 ( 15.2%) |
| Severe insomnia | 56 ( 2.8%) | 46 ( 3.0%) | 10 ( 2.1%) |
| Missing | 0 ( 0.0%) | 0 ( 0.0%) | 0 ( 0.0%) |

| Table 42.2 - ISI categories (by age) | | | |
| --- | --- | --- | --- |
| ES | | | |
|  | Age 18-30 (N = 892) | Age 31-65 (N = 1662) | Age >65 (N = 98) |
| Absence of insomnia | 353 ( 39.6%) | 836 ( 50.3%) | 58 ( 59.2%) |
| Subthreshold insomnia | 301 ( 33.7%) | 486 ( 29.2%) | 19 ( 19.4%) |
| Moderate insomnia | 160 ( 17.9%) | 213 ( 12.8%) | 9 ( 9.2%) |
| Severe insomnia | 27 ( 3.0%) | 43 ( 2.6%) | 0 ( 0.0%) |
| Missing | 51 ( 5.7%) | 84 ( 5.1%) | 12 ( 12.2%) |

| Table 42.2 - ISI categories (by age) | | | |
| --- | --- | --- | --- |
| MAS | | | |
|  | Age 18-30 (N = 666) | Age 31-65 (N = 1258) | Age >65 (N = 65) |
| Absence of insomnia | 280 ( 42.0%) | 654 ( 52.0%) | 43 ( 66.2%) |
| Subthreshold insomnia | 234 ( 35.1%) | 394 ( 31.3%) | 14 ( 21.5%) |
| Moderate insomnia | 129 ( 19.4%) | 177 ( 14.1%) | 8 ( 12.3%) |
| Severe insomnia | 23 ( 3.5%) | 33 ( 2.6%) | 0 ( 0.0%) |
| Missing | 0 ( 0.0%) | 0 ( 0.0%) | 0 ( 0.0%) |

| Table 43.1 - ISI total score (overall and by sex) | | | |
| --- | --- | --- | --- |
| ES | | | |
|  | Total (N = 2652) | Female (N = 2023) | Male (N = 629) |
| n | 2505 | 1907 | 598 |
| Mean | 8.4 | 8.6 | 7.7 |
| StD | 6.19 | 6.13 | 6.34 |
| Median | 8.0 | 8.0 | 6.0 |
| 25%/75% quantile | 3.0/13.0 | 4.0/13.0 | 2.0/12.0 |
| Min/Max | 0/28 | 0/28 | 0/28 |

| Table 43.1 - ISI total score (overall and by sex) | | | |
| --- | --- | --- | --- |
| MAS | | | |
|  | Total (N = 1989) | Female (N = 1515) | Male (N = 474) |
| n | 1989 | 1515 | 474 |
| Mean | 8.4 | 8.6 | 7.7 |
| StD | 6.20 | 6.18 | 6.23 |
| Median | 8.0 | 8.0 | 6.0 |
| 25%/75% quantile | 3.0/13.0 | 4.0/13.0 | 3.0/12.0 |
| Min/Max | 0/28 | 0/28 | 0/28 |

| Table 43.2 - ISI total score (by age) | | | |
| --- | --- | --- | --- |
| ES | | | |
|  | Age 18-30 (N = 892) | Age 31-65 (N = 1662) | Age >65 (N = 98) |
| n | 841 | 1578 | 86 |
| Mean | 9.4 | 8.0 | 6.1 |
| StD | 6.17 | 6.17 | 5.28 |
| Median | 9.0 | 7.0 | 4.5 |
| 25%/75% quantile | 4.0/14.0 | 3.0/12.0 | 2.0/ 9.8 |
| Min/Max | 0/28 | 0/28 | 0/18 |

| Table 43.2 - ISI total score (by age) | | | |
| --- | --- | --- | --- |
| MAS | | | |
|  | Age 18-30 (N = 666) | Age 31-65 (N = 1258) | Age >65 (N = 65) |
| n | 666 | 1258 | 65 |
| Mean | 9.3 | 8.0 | 6.3 |
| StD | 6.23 | 6.17 | 5.43 |
| Median | 9.0 | 7.0 | 5.0 |
| 25%/75% quantile | 4.0/14.0 | 3.0/13.0 | 2.0/10.0 |
| Min/Max | 0/27 | 0/28 | 0/18 |

| Table 44.1 - SHI (overall and by sex) | | | |
| --- | --- | --- | --- |
| ES | | | |
|  | Total (N = 2652) | Female (N = 2023) | Male (N = 629) |
| n | 2416 | 1840 | 576 |
| Mean | 12.7 | 12.8 | 12.4 |
| StD | 7.26 | 7.27 | 7.20 |
| Median | 12.0 | 12.0 | 11.5 |
| 25%/75% quantile | 7.0/17.0 | 7.0/17.0 | 7.0/17.0 |
| Min/Max | 0/52 | 0/52 | 0/38 |

| Table 44.1 - SHI (overall and by sex) | | | |
| --- | --- | --- | --- |
| MAS | | | |
|  | Total (N = 1989) | Female (N = 1515) | Male (N = 474) |
| n | 1989 | 1515 | 474 |
| Mean | 12.8 | 12.9 | 12.4 |
| StD | 7.32 | 7.37 | 7.15 |
| Median | 12.0 | 12.0 | 11.0 |
| 25%/75% quantile | 7.0/18.0 | 7.0/18.0 | 7.0/17.0 |
| Min/Max | 0/52 | 0/52 | 0/38 |

| Table 44.2 - SHI (by age) | | | |
| --- | --- | --- | --- |
| ES | | | |
|  | Age 18-30 (N = 892) | Age 31-65 (N = 1662) | Age >65 (N = 98) |
| n | 802 | 1529 | 85 |
| Mean | 15.7 | 11.4 | 8.5 |
| StD | 7.20 | 6.84 | 5.83 |
| Median | 15.0 | 10.0 | 8.0 |
| 25%/75% quantile | 11.0/20.0 | 6.0/15.0 | 4.0/12.0 |
| Min/Max | 0/38 | 0/52 | 0/25 |

| Table 44.2 - SHI (by age) | | | |
| --- | --- | --- | --- |
| MAS | | | |
|  | Age 18-30 (N = 666) | Age 31-65 (N = 1258) | Age >65 (N = 65) |
| n | 666 | 1258 | 65 |
| Mean | 15.9 | 11.4 | 8.5 |
| StD | 7.24 | 6.89 | 5.69 |
| Median | 15.0 | 10.0 | 8.0 |
| 25%/75% quantile | 11.0/20.0 | 6.0/15.0 | 4.0/13.0 |
| Min/Max | 0/38 | 0/52 | 0/22 |

| Table 44.3 - SHI (by ISI group) | | | | | |
| --- | --- | --- | --- | --- | --- |
| ES | | | | | |
|  | Absence of insomnia (N = 1247) | Subthreshold insomnia (N = 806) | Moderate insomnia (N = 382) | Severe insomnia (N = 70) | Unknown (N = 147) |
| n | 1196 | 768 | 361 | 68 | 23 |
| Mean | 10.2 | 13.8 | 16.8 | 20.6 | 13.1 |
| StD | 6.03 | 7.25 | 7.17 | 8.70 | 7.47 |
| Median | 10.0 | 13.0 | 16.0 | 21.0 | 12.0 |
| 25%/75% quantile | 6.0/14.0 | 8.0/18.0 | 11.0/22.0 | 15.0/26.0 | 9.0/16.0 |
| Min/Max | 0/41 | 0/38 | 3/38 | 4/52 | 1/29 |

| Table 44.3 - SHI (by ISI group) | | | | |
| --- | --- | --- | --- | --- |
| MAS | | | | |
|  | Absence of insomnia (N = 977) | Subthreshold insomnia (N = 642) | Moderate insomnia (N = 314) | Severe insomnia (N = 56) |
| n | 977 | 642 | 314 | 56 |
| Mean | 10.3 | 14.0 | 17.0 | 20.9 |
| StD | 6.07 | 7.30 | 7.11 | 8.72 |
| Median | 10.0 | 13.0 | 17.0 | 21.0 |
| 25%/75% quantile | 6.0/14.0 | 9.0/18.0 | 11.2/22.0 | 15.8/26.2 |
| Min/Max | 0/41 | 0/38 | 3/38 | 6/52 |

| Table 45.1 - MEQ (categories, overall and by sex) | | | |
| --- | --- | --- | --- |
| ES | | | |
|  | Total (N = 2652) | Female (N = 2023) | Male (N = 629) |
| Evening type | 313 ( 11.8%) | 236 ( 11.7%) | 77 ( 12.2%) |
| Intermediate type | 1591 ( 60.0%) | 1227 ( 60.7%) | 364 ( 57.9%) |
| Morning type | 480 ( 18.1%) | 352 ( 17.4%) | 128 ( 20.3%) |
| Missing | 268 ( 10.1%) | 208 ( 10.3%) | 60 ( 9.5%) |

| Table 45.1 - MEQ (categories, overall and by sex) | | | |
| --- | --- | --- | --- |
| MAS | | | |
|  | Total (N = 1989) | Female (N = 1515) | Male (N = 474) |
| Evening type | 270 ( 13.6%) | 205 ( 13.5%) | 65 ( 13.7%) |
| Intermediate type | 1329 ( 66.8%) | 1025 ( 67.7%) | 304 ( 64.1%) |
| Morning type | 390 ( 19.6%) | 285 ( 18.8%) | 105 ( 22.2%) |
| Missing | 0 ( 0.0%) | 0 ( 0.0%) | 0 ( 0.0%) |

| Table 45.2 - MEQ (categories, by age) | | | |
| --- | --- | --- | --- |
| ES | | | |
|  | Age 18-30 (N = 892) | Age 31-65 (N = 1662) | Age >65 (N = 98) |
| Evening type | 151 ( 16.9%) | 157 ( 9.4%) | 5 ( 5.1%) |
| Intermediate type | 555 ( 62.2%) | 983 ( 59.1%) | 53 ( 54.1%) |
| Morning type | 81 ( 9.1%) | 371 ( 22.3%) | 28 ( 28.6%) |
| Missing | 105 ( 11.8%) | 151 ( 9.1%) | 12 ( 12.2%) |

| Table 45.2 - MEQ (categories, by age) | | | |
| --- | --- | --- | --- |
| MAS | | | |
|  | Age 18-30 (N = 666) | Age 31-65 (N = 1258) | Age >65 (N = 65) |
| Evening type | 133 ( 20.0%) | 134 ( 10.7%) | 3 ( 4.6%) |
| Intermediate type | 467 ( 70.1%) | 822 ( 65.3%) | 40 ( 61.5%) |
| Morning type | 66 ( 9.9%) | 302 ( 24.0%) | 22 ( 33.8%) |
| Missing | 0 ( 0.0%) | 0 ( 0.0%) | 0 ( 0.0%) |

| Table 45.3 - MEQ (categories, by ISI group) | | | | | |
| --- | --- | --- | --- | --- | --- |
| ES | | | | | |
|  | Absence of insomnia (N = 1247) | Subthreshold insomnia (N = 806) | Moderate insomnia (N = 382) | Severe insomnia (N = 70) | Unknown (N = 147) |
| Evening type | 110 ( 8.8%) | 110 ( 13.6%) | 71 ( 18.6%) | 22 ( 31.4%) | 0 ( 0.0%) |
| Intermediate type | 798 ( 64.0%) | 508 ( 63.0%) | 232 ( 60.7%) | 33 ( 47.1%) | 20 ( 13.6%) |
| Morning type | 274 ( 22.0%) | 138 ( 17.1%) | 51 ( 13.4%) | 13 ( 18.6%) | 4 ( 2.7%) |
| Missing | 65 ( 5.2%) | 50 ( 6.2%) | 28 ( 7.3%) | 2 ( 2.9%) | 123 ( 83.7%) |

| Table 45.3 - MEQ (categories, by ISI group) | | | | |
| --- | --- | --- | --- | --- |
| MAS | | | | |
|  | Absence of insomnia (N = 977) | Subthreshold insomnia (N = 642) | Moderate insomnia (N = 314) | Severe insomnia (N = 56) |
| Evening type | 92 ( 9.4%) | 96 ( 15.0%) | 64 ( 20.4%) | 18 ( 32.1%) |
| Intermediate type | 667 ( 68.3%) | 432 ( 67.3%) | 201 ( 64.0%) | 29 ( 51.8%) |
| Morning type | 218 ( 22.3%) | 114 ( 17.8%) | 49 ( 15.6%) | 9 ( 16.1%) |
| Missing | 0 ( 0.0%) | 0 ( 0.0%) | 0 ( 0.0%) | 0 ( 0.0%) |

| Table 46.1 - ERQ-CR (overall and by sex) | | | |
| --- | --- | --- | --- |
| ES | | | |
|  | Total (N = 2652) | Female (N = 2023) | Male (N = 629) |
| n | 2291 | 1744 | 547 |
| Mean | 28.9 | 29.1 | 28.1 |
| StD | 7.32 | 7.29 | 7.37 |
| Median | 30.0 | 30.0 | 29.0 |
| 25%/75% quantile | 24.0/34.0 | 24.0/34.0 | 24.0/33.0 |
| Min/Max | 6/42 | 6/42 | 6/42 |

| Table 46.1 - ERQ-CR (overall and by sex) | | | |
| --- | --- | --- | --- |
| MAS | | | |
|  | Total (N = 1989) | Female (N = 1515) | Male (N = 474) |
| n | 1989 | 1515 | 474 |
| Mean | 28.9 | 29.2 | 28.1 |
| StD | 7.25 | 7.23 | 7.27 |
| Median | 30.0 | 30.0 | 29.0 |
| 25%/75% quantile | 24.0/34.0 | 25.0/34.0 | 24.0/33.0 |
| Min/Max | 6/42 | 6/42 | 6/42 |

| Table 46.2 - ERQ-CR (by age) | | | |
| --- | --- | --- | --- |
| ES | | | |
|  | Age 18-30 (N = 892) | Age 31-65 (N = 1662) | Age >65 (N = 98) |
| n | 757 | 1448 | 86 |
| Mean | 27.7 | 29.5 | 28.7 |
| StD | 7.25 | 7.21 | 8.64 |
| Median | 28.0 | 30.0 | 30.0 |
| 25%/75% quantile | 23.0/33.0 | 25.0/35.0 | 24.0/33.0 |
| Min/Max | 6/42 | 6/42 | 6/42 |

| Table 46.2 - ERQ-CR (by age) | | | |
| --- | --- | --- | --- |
| MAS | | | |
|  | Age 18-30 (N = 666) | Age 31-65 (N = 1258) | Age >65 (N = 65) |
| n | 666 | 1258 | 65 |
| Mean | 27.8 | 29.6 | 28.2 |
| StD | 7.20 | 7.13 | 8.74 |
| Median | 28.0 | 30.0 | 30.0 |
| 25%/75% quantile | 24.0/33.0 | 25.0/35.0 | 24.0/33.0 |
| Min/Max | 6/42 | 6/42 | 6/42 |

| Table 46.3 - ERQ-CR (by ISI group) | | | | | |
| --- | --- | --- | --- | --- | --- |
| ES | | | | | |
|  | Absence of insomnia (N = 1247) | Subthreshold insomnia (N = 806) | Moderate insomnia (N = 382) | Severe insomnia (N = 70) | Unknown (N = 147) |
| n | 1134 | 729 | 342 | 65 | 21 |
| Mean | 29.5 | 28.8 | 27.5 | 26.5 | 27.6 |
| StD | 7.30 | 7.09 | 7.26 | 8.99 | 7.40 |
| Median | 30.0 | 29.0 | 28.0 | 27.0 | 27.0 |
| 25%/75% quantile | 25.0/35.0 | 24.0/33.0 | 23.0/32.0 | 20.0/35.0 | 20.0/32.0 |
| Min/Max | 6/42 | 6/42 | 6/42 | 6/42 | 16/42 |

| Table 46.3 - ERQ-CR (by ISI group) | | | | |
| --- | --- | --- | --- | --- |
| MAS | | | | |
|  | Absence of insomnia (N = 977) | Subthreshold insomnia (N = 642) | Moderate insomnia (N = 314) | Severe insomnia (N = 56) |
| n | 977 | 642 | 314 | 56 |
| Mean | 29.7 | 28.8 | 27.4 | 25.8 |
| StD | 7.12 | 7.05 | 7.29 | 9.28 |
| Median | 30.0 | 29.0 | 28.0 | 26.0 |
| 25%/75% quantile | 26.0/35.0 | 24.0/33.0 | 23.0/32.0 | 19.0/34.2 |
| Min/Max | 6/42 | 6/42 | 6/42 | 6/42 |

| Table 47.1 - ERQ-ES (overall and by sex) | | | |
| --- | --- | --- | --- |
| ES | | | |
|  | Total (N = 2652) | Female (N = 2023) | Male (N = 629) |
| n | 2294 | 1746 | 548 |
| Mean | 13.3 | 12.7 | 14.9 |
| StD | 5.43 | 5.41 | 5.16 |
| Median | 13.0 | 12.0 | 15.0 |
| 25%/75% quantile | 9.0/17.0 | 8.0/17.0 | 11.0/19.0 |
| Min/Max | 4/28 | 4/28 | 4/28 |

| Table 47.1 - ERQ-ES (overall and by sex) | | | |
| --- | --- | --- | --- |
| MAS | | | |
|  | Total (N = 1989) | Female (N = 1515) | Male (N = 474) |
| n | 1989 | 1515 | 474 |
| Mean | 13.2 | 12.6 | 14.9 |
| StD | 5.42 | 5.39 | 5.15 |
| Median | 13.0 | 12.0 | 15.0 |
| 25%/75% quantile | 9.0/17.0 | 8.0/16.0 | 11.0/19.0 |
| Min/Max | 4/28 | 4/28 | 4/28 |

| Table 47.2 - ERQ-ES (by age) | | | |
| --- | --- | --- | --- |
| ES | | | |
|  | Age 18-30 (N = 892) | Age 31-65 (N = 1662) | Age >65 (N = 98) |
| n | 757 | 1451 | 86 |
| Mean | 13.4 | 13.1 | 14.6 |
| StD | 5.33 | 5.52 | 4.48 |
| Median | 13.0 | 13.0 | 15.0 |
| 25%/75% quantile | 10.0/17.0 | 9.0/17.0 | 12.0/18.0 |
| Min/Max | 4/28 | 4/28 | 4/24 |

| Table 47.2 - ERQ-ES (by age) | | | |
| --- | --- | --- | --- |
| MAS | | | |
|  | Age 18-30 (N = 666) | Age 31-65 (N = 1258) | Age >65 (N = 65) |
| n | 666 | 1258 | 65 |
| Mean | 13.3 | 13.0 | 14.0 |
| StD | 5.41 | 5.47 | 4.41 |
| Median | 13.0 | 13.0 | 15.0 |
| 25%/75% quantile | 9.0/17.0 | 9.0/17.0 | 11.0/17.0 |
| Min/Max | 4/28 | 4/28 | 4/24 |

| Table 47.3 - ERQ-ES (by ISI group) | | | | | |
| --- | --- | --- | --- | --- | --- |
| ES | | | | | |
|  | Absence of insomnia (N = 1247) | Subthreshold insomnia (N = 806) | Moderate insomnia (N = 382) | Severe insomnia (N = 70) | Unknown (N = 147) |
| n | 1136 | 729 | 343 | 65 | 21 |
| Mean | 12.7 | 13.5 | 14.3 | 14.4 | 15.7 |
| StD | 5.33 | 5.44 | 5.52 | 5.70 | 4.90 |
| Median | 12.0 | 13.0 | 14.0 | 14.0 | 16.0 |
| 25%/75% quantile | 9.0/17.0 | 9.0/17.0 | 10.0/18.0 | 10.0/18.0 | 12.0/19.0 |
| Min/Max | 4/28 | 4/28 | 4/28 | 4/28 | 7/23 |

| Table 47.3 - ERQ-ES (by ISI group) | | | | |
| --- | --- | --- | --- | --- |
| MAS | | | | |
|  | Absence of insomnia (N = 977) | Subthreshold insomnia (N = 642) | Moderate insomnia (N = 314) | Severe insomnia (N = 56) |
| n | 977 | 642 | 314 | 56 |
| Mean | 12.5 | 13.4 | 14.4 | 14.0 |
| StD | 5.29 | 5.40 | 5.57 | 5.75 |
| Median | 12.0 | 13.0 | 14.0 | 13.5 |
| 25%/75% quantile | 8.0/16.0 | 9.0/17.0 | 10.0/18.0 | 10.0/18.0 |
| Min/Max | 4/28 | 4/28 | 4/28 | 4/28 |

| Table 48.1 - PSS (overall and by sex) | | | |
| --- | --- | --- | --- |
| ES | | | |
|  | Total (N = 2652) | Female (N = 2023) | Male (N = 629) |
| n | 2226 | 1695 | 531 |
| Mean | 19.5 | 20.4 | 16.7 |
| StD | 7.29 | 7.10 | 7.21 |
| Median | 19.0 | 20.0 | 16.0 |
| 25%/75% quantile | 14.0/25.0 | 16.0/25.0 | 12.0/22.0 |
| Min/Max | 0/40 | 1/40 | 0/39 |

| Table 48.1 - PSS (overall and by sex) | | | |
| --- | --- | --- | --- |
| MAS | | | |
|  | Total (N = 1989) | Female (N = 1515) | Male (N = 474) |
| n | 1989 | 1515 | 474 |
| Mean | 19.7 | 20.6 | 16.9 |
| StD | 7.33 | 7.15 | 7.19 |
| Median | 20.0 | 20.0 | 16.0 |
| 25%/75% quantile | 14.0/25.0 | 16.0/26.0 | 12.0/22.0 |
| Min/Max | 0/40 | 1/40 | 0/39 |

| Table 48.2 - PSS (by age) | | | |
| --- | --- | --- | --- |
| ES | | | |
|  | Age 18-30 (N = 892) | Age 31-65 (N = 1662) | Age >65 (N = 98) |
| n | 739 | 1405 | 82 |
| Mean | 21.2 | 18.7 | 17.7 |
| StD | 7.28 | 7.20 | 6.35 |
| Median | 21.0 | 19.0 | 18.0 |
| 25%/75% quantile | 17.0/26.0 | 13.0/24.0 | 13.0/21.8 |
| Min/Max | 0/40 | 0/39 | 4/33 |

| Table 48.2 - PSS (by age) | | | |
| --- | --- | --- | --- |
| MAS | | | |
|  | Age 18-30 (N = 666) | Age 31-65 (N = 1258) | Age >65 (N = 65) |
| n | 666 | 1258 | 65 |
| Mean | 21.5 | 18.8 | 17.5 |
| StD | 7.28 | 7.21 | 6.77 |
| Median | 21.0 | 19.0 | 18.0 |
| 25%/75% quantile | 17.0/27.0 | 13.0/24.0 | 13.0/21.0 |
| Min/Max | 0/40 | 1/39 | 4/33 |

| Table 48.3 - PSS (by ISI group) | | | | | |
| --- | --- | --- | --- | --- | --- |
| ES | | | | | |
|  | Absence of insomnia (N = 1247) | Subthreshold insomnia (N = 806) | Moderate insomnia (N = 382) | Severe insomnia (N = 70) | Unknown (N = 147) |
| n | 1101 | 712 | 331 | 61 | 21 |
| Mean | 16.5 | 21.1 | 24.5 | 27.9 | 16.8 |
| StD | 6.48 | 6.61 | 6.44 | 6.17 | 5.72 |
| Median | 16.0 | 21.0 | 25.0 | 28.0 | 18.0 |
| 25%/75% quantile | 12.0/21.0 | 17.0/26.0 | 20.0/29.0 | 24.0/31.0 | 15.0/20.0 |
| Min/Max | 0/37 | 0/40 | 5/38 | 12/39 | 4/25 |

| Table 48.3 - PSS (by ISI group) | | | | |
| --- | --- | --- | --- | --- |
| MAS | | | | |
|  | Absence of insomnia (N = 977) | Subthreshold insomnia (N = 642) | Moderate insomnia (N = 314) | Severe insomnia (N = 56) |
| n | 977 | 642 | 314 | 56 |
| Mean | 16.6 | 21.2 | 24.6 | 27.8 |
| StD | 6.56 | 6.59 | 6.45 | 6.04 |
| Median | 16.0 | 21.0 | 25.0 | 27.5 |
| 25%/75% quantile | 12.0/21.0 | 17.0/26.0 | 20.0/29.0 | 24.8/31.0 |
| Min/Max | 0/37 | 0/40 | 5/38 | 12/39 |

| Table 49.1 - PSS categories (overall and by sex) | | | |
| --- | --- | --- | --- |
| ES | | | |
|  | Total (N = 2652) | Female (N = 2023) | Male (N = 629) |
| Low stress | 257 ( 9.7%) | 153 ( 7.6%) | 104 ( 16.5%) |
| Average stress | 323 ( 12.2%) | 205 ( 10.1%) | 118 ( 18.8%) |
| High stress | 416 ( 15.7%) | 320 ( 15.8%) | 96 ( 15.3%) |
| Very high stress | 1227 ( 46.3%) | 1017 ( 50.3%) | 210 ( 33.4%) |
| Missing | 429 ( 16.2%) | 328 ( 16.2%) | 101 ( 16.1%) |

| Table 49.1 - PSS categories (overall and by sex) | | | |
| --- | --- | --- | --- |
| MAS | | | |
|  | Total (N = 1989) | Female (N = 1515) | Male (N = 474) |
| Low stress | 229 ( 11.5%) | 136 ( 9.0%) | 93 ( 19.6%) |
| Average stress | 280 ( 14.1%) | 176 ( 11.6%) | 104 ( 21.9%) |
| High stress | 366 ( 18.4%) | 281 ( 18.5%) | 85 ( 17.9%) |
| Very high stress | 1112 ( 55.9%) | 922 ( 60.9%) | 190 ( 40.1%) |
| Missing | 2 ( 0.1%) | 0 ( 0.0%) | 2 ( 0.4%) |

| Table 49.2 - PSS categories (by age) | | | |
| --- | --- | --- | --- |
| ES | | | |
|  | Age 18-30 (N = 892) | Age 31-65 (N = 1662) | Age >65 (N = 98) |
| Low stress | 52 ( 5.8%) | 191 ( 11.5%) | 14 ( 14.3%) |
| Average stress | 81 ( 9.1%) | 231 ( 13.9%) | 11 ( 11.2%) |
| High stress | 123 ( 13.8%) | 273 ( 16.4%) | 20 ( 20.4%) |
| Very high stress | 481 ( 53.9%) | 709 ( 42.7%) | 37 ( 37.8%) |
| Missing | 155 ( 17.4%) | 258 ( 15.5%) | 16 ( 16.3%) |

| Table 49.2 - PSS categories (by age) | | | |
| --- | --- | --- | --- |
| MAS | | | |
|  | Age 18-30 (N = 666) | Age 31-65 (N = 1258) | Age >65 (N = 65) |
| Low stress | 44 ( 6.6%) | 172 ( 13.7%) | 13 ( 20.0%) |
| Average stress | 67 ( 10.1%) | 203 ( 16.1%) | 10 ( 15.4%) |
| High stress | 113 ( 17.0%) | 241 ( 19.2%) | 12 ( 18.5%) |
| Very high stress | 440 ( 66.1%) | 642 ( 51.0%) | 30 ( 46.2%) |
| Missing | 2 ( 0.3%) | 0 ( 0.0%) | 0 ( 0.0%) |

| Table 49.3 - PSS categories (by ISI group) | | | | | |
| --- | --- | --- | --- | --- | --- |
| ES | | | | | |
|  | Absence of insomnia (N = 1247) | Subthreshold insomnia (N = 806) | Moderate insomnia (N = 382) | Severe insomnia (N = 70) | Unknown (N = 147) |
| Low stress | 209 ( 16.8%) | 37 ( 4.6%) | 7 ( 1.8%) | 0 ( 0.0%) | 4 ( 2.7%) |
| Average stress | 228 ( 18.3%) | 78 ( 9.7%) | 14 ( 3.7%) | 2 ( 2.9%) | 1 ( 0.7%) |
| High stress | 237 ( 19.0%) | 131 ( 16.3%) | 40 ( 10.5%) | 1 ( 1.4%) | 7 ( 4.8%) |
| Very high stress | 426 ( 34.2%) | 464 ( 57.6%) | 270 ( 70.7%) | 58 ( 82.9%) | 9 ( 6.1%) |
| Missing | 147 ( 11.8%) | 96 ( 11.9%) | 51 ( 13.4%) | 9 ( 12.9%) | 126 ( 85.7%) |

| Table 49.3 - PSS categories (by ISI group) | | | | |
| --- | --- | --- | --- | --- |
| MAS | | | | |
|  | Absence of insomnia (N = 977) | Subthreshold insomnia (N = 642) | Moderate insomnia (N = 314) | Severe insomnia (N = 56) |
| Low stress | 188 ( 19.2%) | 34 ( 5.3%) | 7 ( 2.2%) | 0 ( 0.0%) |
| Average stress | 199 ( 20.4%) | 67 ( 10.4%) | 12 ( 3.8%) | 2 ( 3.6%) |
| High stress | 207 ( 21.2%) | 121 ( 18.8%) | 37 ( 11.8%) | 1 ( 1.8%) |
| Very high stress | 382 ( 39.1%) | 419 ( 65.3%) | 258 ( 82.2%) | 53 ( 94.6%) |
| Missing | 1 ( 0.1%) | 1 ( 0.2%) | 0 ( 0.0%) | 0 ( 0.0%) |

| Table 50.1 - HADS Anxiety (overall and by sex) | | | |
| --- | --- | --- | --- |
| ES | | | |
|  | Total (N = 2652) | Female (N = 2023) | Male (N = 629) |
| n | 2184 | 1660 | 524 |
| Mean | 7.8 | 8.2 | 6.5 |
| StD | 3.87 | 3.83 | 3.72 |
| Median | 7.0 | 8.0 | 6.0 |
| 25%/75% quantile | 5.0/10.0 | 5.0/11.0 | 4.0/ 8.0 |
| Min/Max | 0/21 | 0/21 | 0/21 |

| Table 50.1 - HADS Anxiety (overall and by sex) | | | |
| --- | --- | --- | --- |
| MAS | | | |
|  | Total (N = 1989) | Female (N = 1515) | Male (N = 474) |
| n | 1989 | 1515 | 474 |
| Mean | 7.9 | 8.3 | 6.6 |
| StD | 3.89 | 3.86 | 3.72 |
| Median | 7.0 | 8.0 | 6.0 |
| 25%/75% quantile | 5.0/10.0 | 5.0/11.0 | 4.0/ 8.0 |
| Min/Max | 0/21 | 0/21 | 0/21 |

| Table 50.2 - HADS Anxiety (by age) | | | |
| --- | --- | --- | --- |
| ES | | | |
|  | Age 18-30 (N = 892) | Age 31-65 (N = 1662) | Age >65 (N = 98) |
| n | 728 | 1374 | 82 |
| Mean | 8.5 | 7.5 | 6.4 |
| StD | 4.05 | 3.75 | 3.36 |
| Median | 8.0 | 7.0 | 6.0 |
| 25%/75% quantile | 6.0/11.0 | 5.0/10.0 | 4.2/ 8.0 |
| Min/Max | 0/21 | 0/21 | 0/18 |

| Table 50.2 - HADS Anxiety (by age) | | | |
| --- | --- | --- | --- |
| MAS | | | |
|  | Age 18-30 (N = 666) | Age 31-65 (N = 1258) | Age >65 (N = 65) |
| n | 666 | 1258 | 65 |
| Mean | 8.6 | 7.6 | 6.7 |
| StD | 4.08 | 3.75 | 3.60 |
| Median | 8.0 | 7.0 | 6.0 |
| 25%/75% quantile | 6.0/11.0 | 5.0/10.0 | 5.0/ 8.0 |
| Min/Max | 0/21 | 0/21 | 0/18 |

| Table 50.3 - HADS Anxiety (by ISI group) | | | | | |
| --- | --- | --- | --- | --- | --- |
| ES | | | | | |
|  | Absence of insomnia (N = 1247) | Subthreshold insomnia (N = 806) | Moderate insomnia (N = 382) | Severe insomnia (N = 70) | Unknown (N = 147) |
| n | 1082 | 695 | 325 | 61 | 21 |
| Mean | 6.1 | 8.6 | 10.9 | 14.0 | 6.2 |
| StD | 3.11 | 3.46 | 3.57 | 3.60 | 3.03 |
| Median | 6.0 | 8.0 | 11.0 | 14.0 | 6.0 |
| 25%/75% quantile | 4.0/ 8.0 | 6.0/11.0 | 8.0/13.0 | 12.0/16.0 | 4.0/ 8.0 |
| Min/Max | 0/20 | 0/19 | 0/20 | 7/21 | 2/13 |

| Table 50.3 - HADS Anxiety (by ISI group) | | | | |
| --- | --- | --- | --- | --- |
| MAS | | | | |
|  | Absence of insomnia (N = 977) | Subthreshold insomnia (N = 642) | Moderate insomnia (N = 314) | Severe insomnia (N = 56) |
| n | 977 | 642 | 314 | 56 |
| Mean | 6.1 | 8.5 | 10.9 | 14.1 |
| StD | 3.13 | 3.46 | 3.57 | 3.56 |
| Median | 6.0 | 8.0 | 11.0 | 14.0 |
| 25%/75% quantile | 4.0/ 8.0 | 6.0/11.0 | 8.0/13.0 | 11.8/16.2 |
| Min/Max | 0/20 | 0/19 | 0/20 | 8/21 |

| Table 51.1 - HADS Anxiety (categories, overall and by sex) | | | |
| --- | --- | --- | --- |
| ES | | | |
|  | Total (N = 2652) | Female (N = 2023) | Male (N = 629) |
| Normal | 1122 ( 42.3%) | 783 ( 38.7%) | 339 ( 53.9%) |
| Borderline | 542 ( 20.4%) | 440 ( 21.7%) | 102 ( 16.2%) |
| Abnormal | 499 ( 18.8%) | 429 ( 21.2%) | 70 ( 11.1%) |
| Missing | 489 ( 18.4%) | 371 ( 18.3%) | 118 ( 18.8%) |

| Table 51.1 - HADS Anxiety (categories, overall and by sex) | | | |
| --- | --- | --- | --- |
| MAS | | | |
|  | Total (N = 1989) | Female (N = 1515) | Male (N = 474) |
| Normal | 1017 ( 51.1%) | 711 ( 46.9%) | 306 ( 64.6%) |
| Borderline | 492 ( 24.7%) | 397 ( 26.2%) | 95 ( 20.0%) |
| Abnormal | 462 ( 23.2%) | 399 ( 26.3%) | 63 ( 13.3%) |
| Missing | 18 ( 0.9%) | 8 ( 0.5%) | 10 ( 2.1%) |

| Table 51.2 - HADS Anxiety (categories, by age) | | | |
| --- | --- | --- | --- |
| ES | | | |
|  | Age 18-30 (N = 892) | Age 31-65 (N = 1662) | Age >65 (N = 98) |
| Normal | 315 ( 35.3%) | 749 ( 45.1%) | 58 ( 59.2%) |
| Borderline | 198 ( 22.2%) | 330 ( 19.9%) | 14 ( 14.3%) |
| Abnormal | 208 ( 23.3%) | 283 ( 17.0%) | 8 ( 8.2%) |
| Missing | 171 ( 19.2%) | 300 ( 18.1%) | 18 ( 18.4%) |

| Table 51.2 - HADS Anxiety (categories, by age) | | | |
| --- | --- | --- | --- |
| MAS | | | |
|  | Age 18-30 (N = 666) | Age 31-65 (N = 1258) | Age >65 (N = 65) |
| Normal | 286 ( 42.9%) | 688 ( 54.7%) | 43 ( 66.2%) |
| Borderline | 181 ( 27.2%) | 299 ( 23.8%) | 12 ( 18.5%) |
| Abnormal | 192 ( 28.8%) | 262 ( 20.8%) | 8 ( 12.3%) |
| Missing | 7 ( 1.1%) | 9 ( 0.7%) | 2 ( 3.1%) |

| Table 51.3 - HADS Anxiety (categories, by ISI group) | | | | | |
| --- | --- | --- | --- | --- | --- |
| ES | | | | | |
|  | Absence of insomnia (N = 1247) | Subthreshold insomnia (N = 806) | Moderate insomnia (N = 382) | Severe insomnia (N = 70) | Unknown (N = 147) |
| Normal | 760 ( 60.9%) | 283 ( 35.1%) | 63 ( 16.5%) | 1 ( 1.4%) | 15 ( 10.2%) |
| Borderline | 211 ( 16.9%) | 228 ( 28.3%) | 89 ( 23.3%) | 10 ( 14.3%) | 4 ( 2.7%) |
| Abnormal | 93 ( 7.5%) | 182 ( 22.6%) | 172 ( 45.0%) | 50 ( 71.4%) | 2 ( 1.4%) |
| Missing | 183 ( 14.7%) | 113 ( 14.0%) | 58 ( 15.2%) | 9 ( 12.9%) | 126 ( 85.7%) |

| Table 51.3 - HADS Anxiety (categories, by ISI group) | | | | |
| --- | --- | --- | --- | --- |
| MAS | | | | |
|  | Absence of insomnia (N = 977) | Subthreshold insomnia (N = 642) | Moderate insomnia (N = 314) | Severe insomnia (N = 56) |
| Normal | 686 ( 70.2%) | 270 ( 42.1%) | 61 ( 19.4%) | 0 ( 0.0%) |
| Borderline | 191 ( 19.5%) | 204 ( 31.8%) | 87 ( 27.7%) | 10 ( 17.9%) |
| Abnormal | 84 ( 8.6%) | 167 ( 26.0%) | 165 ( 52.5%) | 46 ( 82.1%) |
| Missing | 16 ( 1.6%) | 1 ( 0.2%) | 1 ( 0.3%) | 0 ( 0.0%) |

| Table 52.1 - HADS Depression (overall and by sex) | | | |
| --- | --- | --- | --- |
| ES | | | |
|  | Total (N = 2652) | Female (N = 2023) | Male (N = 629) |
| n | 2186 | 1661 | 525 |
| Mean | 6.0 | 6.1 | 5.5 |
| StD | 3.72 | 3.71 | 3.71 |
| Median | 6.0 | 6.0 | 5.0 |
| 25%/75% quantile | 3.0/8.0 | 3.0/9.0 | 3.0/8.0 |
| Min/Max | 0/21 | 0/21 | 0/19 |

| Table 52.1 - HADS Depression (overall and by sex) | | | |
| --- | --- | --- | --- |
| MAS | | | |
|  | Total (N = 1989) | Female (N = 1515) | Male (N = 474) |
| n | 1989 | 1515 | 474 |
| Mean | 6.0 | 6.2 | 5.5 |
| StD | 3.72 | 3.72 | 3.70 |
| Median | 6.0 | 6.0 | 5.0 |
| 25%/75% quantile | 3.0/8.0 | 3.0/9.0 | 3.0/8.0 |
| Min/Max | 0/21 | 0/21 | 0/19 |

| Table 52.2 - HADS Depression (by age) | | | |
| --- | --- | --- | --- |
| ES | | | |
|  | Age 18-30 (N = 892) | Age 31-65 (N = 1662) | Age >65 (N = 98) |
| n | 729 | 1375 | 82 |
| Mean | 6.2 | 5.9 | 5.1 |
| StD | 3.71 | 3.74 | 3.20 |
| Median | 6.0 | 6.0 | 5.0 |
| 25%/75% quantile | 3.0/8.0 | 3.0/8.0 | 3.0/7.0 |
| Min/Max | 0/19 | 0/21 | 0/15 |

| Table 52.2 - HADS Depression (by age) | | | |
| --- | --- | --- | --- |
| MAS | | | |
|  | Age 18-30 (N = 666) | Age 31-65 (N = 1258) | Age >65 (N = 65) |
| n | 666 | 1258 | 65 |
| Mean | 6.2 | 5.9 | 5.2 |
| StD | 3.74 | 3.72 | 3.33 |
| Median | 6.0 | 6.0 | 5.0 |
| 25%/75% quantile | 3.0/8.8 | 3.0/8.0 | 3.0/8.0 |
| Min/Max | 0/19 | 0/21 | 0/15 |

| Table 52.3 - HADS Depression (by ISI group) | | | | | |
| --- | --- | --- | --- | --- | --- |
| ES | | | | | |
|  | Absence of insomnia (N = 1247) | Subthreshold insomnia (N = 806) | Moderate insomnia (N = 382) | Severe insomnia (N = 70) | Unknown (N = 147) |
| n | 1083 | 696 | 325 | 61 | 21 |
| Mean | 4.4 | 6.7 | 8.9 | 10.5 | 3.9 |
| StD | 3.15 | 3.25 | 3.53 | 3.90 | 3.02 |
| Median | 4.0 | 6.0 | 9.0 | 11.0 | 3.0 |
| 25%/75% quantile | 2.0/ 6.0 | 4.0/ 9.0 | 7.0/11.0 | 8.0/13.0 | 2.0/ 4.0 |
| Min/Max | 0/19 | 0/17 | 1/19 | 3/21 | 0/12 |

| Table 52.3 - HADS Depression (by ISI group) | | | | |
| --- | --- | --- | --- | --- |
| MAS | | | | |
|  | Absence of insomnia (N = 977) | Subthreshold insomnia (N = 642) | Moderate insomnia (N = 314) | Severe insomnia (N = 56) |
| n | 977 | 642 | 314 | 56 |
| Mean | 4.4 | 6.6 | 8.8 | 10.5 |
| StD | 3.13 | 3.26 | 3.55 | 4.01 |
| Median | 4.0 | 6.0 | 9.0 | 11.0 |
| 25%/75% quantile | 2.0/ 6.0 | 4.0/ 9.0 | 7.0/11.0 | 7.8/13.0 |
| Min/Max | 0/19 | 0/17 | 1/19 | 3/21 |

| Table 53.1 - HADS Depression (categories, overall and by sex) | | | |
| --- | --- | --- | --- |
| ES | | | |
|  | Total (N = 2652) | Female (N = 2023) | Male (N = 629) |
| Normal | 1350 ( 50.9%) | 1005 ( 49.7%) | 345 ( 54.8%) |
| Borderline | 466 ( 17.6%) | 379 ( 18.7%) | 87 ( 13.8%) |
| Abnormal | 261 ( 9.8%) | 204 ( 10.1%) | 57 ( 9.1%) |
| Missing | 575 ( 21.7%) | 435 ( 21.5%) | 140 ( 22.3%) |

| Table 53.1 - HADS Depression (categories, overall and by sex) | | | |
| --- | --- | --- | --- |
| MAS | | | |
|  | Total (N = 1989) | Female (N = 1515) | Male (N = 474) |
| Normal | 1232 ( 61.9%) | 919 ( 60.7%) | 313 ( 66.0%) |
| Borderline | 424 ( 21.3%) | 344 ( 22.7%) | 80 ( 16.9%) |
| Abnormal | 240 ( 12.1%) | 188 ( 12.4%) | 52 ( 11.0%) |
| Missing | 93 ( 4.7%) | 64 ( 4.2%) | 29 ( 6.1%) |

| Table 53.2 - HADS Depression (categories, by age) | | | |
| --- | --- | --- | --- |
| ES | | | |
|  | Age 18-30 (N = 892) | Age 31-65 (N = 1662) | Age >65 (N = 98) |
| Normal | 451 ( 50.6%) | 845 ( 50.8%) | 54 ( 55.1%) |
| Borderline | 159 ( 17.8%) | 291 ( 17.5%) | 16 ( 16.3%) |
| Abnormal | 90 ( 10.1%) | 167 ( 10.0%) | 4 ( 4.1%) |
| Missing | 192 ( 21.5%) | 359 ( 21.6%) | 24 ( 24.5%) |

| Table 53.2 - HADS Depression (categories, by age) | | | |
| --- | --- | --- | --- |
| MAS | | | |
|  | Age 18-30 (N = 666) | Age 31-65 (N = 1258) | Age >65 (N = 65) |
| Normal | 411 ( 61.7%) | 780 ( 62.0%) | 41 ( 63.1%) |
| Borderline | 144 ( 21.6%) | 266 ( 21.1%) | 14 ( 21.5%) |
| Abnormal | 86 ( 12.9%) | 150 ( 11.9%) | 4 ( 6.2%) |
| Missing | 25 ( 3.8%) | 62 ( 4.9%) | 6 ( 9.2%) |

| Table 53.3 - HADS Depression (categories, by ISI group) | | | | | |
| --- | --- | --- | --- | --- | --- |
| ES | | | | | |
|  | Absence of insomnia (N = 1247) | Subthreshold insomnia (N = 806) | Moderate insomnia (N = 382) | Severe insomnia (N = 70) | Unknown (N = 147) |
| Normal | 798 ( 64.0%) | 406 ( 50.4%) | 115 ( 30.1%) | 14 ( 20.0%) | 17 ( 11.6%) |
| Borderline | 150 ( 12.0%) | 186 ( 23.1%) | 113 ( 29.6%) | 15 ( 21.4%) | 2 ( 1.4%) |
| Abnormal | 42 ( 3.4%) | 89 ( 11.0%) | 97 ( 25.4%) | 32 ( 45.7%) | 1 ( 0.7%) |
| Missing | 257 ( 20.6%) | 125 ( 15.5%) | 57 ( 14.9%) | 9 ( 12.9%) | 127 ( 86.4%) |

| Table 53.3 - HADS Depression (categories, by ISI group) | | | | |
| --- | --- | --- | --- | --- |
| MAS | | | | |
|  | Absence of insomnia (N = 977) | Subthreshold insomnia (N = 642) | Moderate insomnia (N = 314) | Severe insomnia (N = 56) |
| Normal | 725 ( 74.2%) | 379 ( 59.0%) | 114 ( 36.3%) | 14 ( 25.0%) |
| Borderline | 137 ( 14.0%) | 168 ( 26.2%) | 106 ( 33.8%) | 13 ( 23.2%) |
| Abnormal | 35 ( 3.6%) | 82 ( 12.8%) | 94 ( 29.9%) | 29 ( 51.8%) |
| Missing | 80 ( 8.2%) | 13 ( 2.0%) | 0 ( 0.0%) | 0 ( 0.0%) |

| Table 54.1 - DBAS-16 (overall and by sex) | | | |
| --- | --- | --- | --- |
| ES | | | |
|  | Total (N = 2652) | Female (N = 2023) | Male (N = 629) |
| n | 2114 | 1611 | 503 |
| Mean | 33.6 | 34.5 | 30.8 |
| StD | 18.26 | 18.06 | 18.64 |
| Median | 32.0 | 33.2 | 27.5 |
| 25%/75% quantile | 19.3/45.3 | 20.3/46.3 | 16.0/41.8 |
| Min/Max | 0/95 | 0/95 | 0/88 |

| Table 54.1 - DBAS-16 (overall and by sex) | | | |
| --- | --- | --- | --- |
| MAS | | | |
|  | Total (N = 1989) | Female (N = 1515) | Male (N = 474) |
| n | 1989 | 1515 | 474 |
| Mean | 33.7 | 34.5 | 31.1 |
| StD | 18.23 | 18.05 | 18.56 |
| Median | 32.1 | 33.2 | 27.8 |
| 25%/75% quantile | 19.3/45.3 | 20.2/46.3 | 16.4/42.2 |
| Min/Max | 0/95 | 0/95 | 0/88 |

| Table 54.2 - DBAS-16 (by age) | | | |
| --- | --- | --- | --- |
| ES | | | |
|  | Age 18-30 (N = 892) | Age 31-65 (N = 1662) | Age >65 (N = 98) |
| n | 711 | 1326 | 77 |
| Mean | 36.4 | 32.5 | 27.0 |
| StD | 18.48 | 17.90 | 18.88 |
| Median | 35.9 | 30.3 | 23.5 |
| 25%/75% quantile | 21.8/47.6 | 18.2/44.3 | 13.2/40.4 |
| Min/Max | 0/95 | 0/95 | 0/91 |

| Table 54.2 - DBAS-16 (by age) | | | |
| --- | --- | --- | --- |
| MAS | | | |
|  | Age 18-30 (N = 666) | Age 31-65 (N = 1258) | Age >65 (N = 65) |
| n | 666 | 1258 | 65 |
| Mean | 36.6 | 32.4 | 28.2 |
| StD | 18.39 | 17.87 | 19.73 |
| Median | 36.0 | 30.1 | 25.4 |
| 25%/75% quantile | 22.0/47.8 | 18.2/44.2 | 13.8/41.2 |
| Min/Max | 0/95 | 0/95 | 0/91 |

| Table 54.3 - DBAS-16 (by ISI group) | | | | | |
| --- | --- | --- | --- | --- | --- |
| ES | | | | | |
|  | Absence of insomnia (N = 1247) | Subthreshold insomnia (N = 806) | Moderate insomnia (N = 382) | Severe insomnia (N = 70) | Unknown (N = 147) |
| n | 1031 | 680 | 332 | 59 | 12 |
| Mean | 25.6 | 36.7 | 47.3 | 63.5 | 26.1 |
| StD | 14.75 | 16.04 | 16.90 | 18.35 | 17.05 |
| Median | 22.8 | 37.1 | 47.0 | 67.8 | 25.5 |
| 25%/75% quantile | 14.7/35.0 | 25.0/47.0 | 36.5/59.6 | 53.2/76.8 | 12.8/36.6 |
| Min/Max | 0/91 | 2/82 | 3/95 | 19/95 | 2/56 |

| Table 54.3 - DBAS-16 (by ISI group) | | | | |
| --- | --- | --- | --- | --- |
| MAS | | | | |
|  | Absence of insomnia (N = 977) | Subthreshold insomnia (N = 642) | Moderate insomnia (N = 314) | Severe insomnia (N = 56) |
| n | 977 | 642 | 314 | 56 |
| Mean | 25.6 | 36.6 | 47.6 | 64.1 |
| StD | 14.61 | 15.97 | 16.81 | 18.05 |
| Median | 22.9 | 37.1 | 47.5 | 67.9 |
| 25%/75% quantile | 14.8/34.8 | 25.0/47.0 | 36.6/59.7 | 53.7/76.9 |
| Min/Max | 0/91 | 2/82 | 3/95 | 19/95 |

| Table 55.1 - CFI (overall and by sex) | | | |
| --- | --- | --- | --- |
| ES | | | |
|  | Total (N = 2652) | Female (N = 2023) | Male (N = 629) |
| n | 2081 | 1587 | 494 |
| Mean | 104.3 | 103.4 | 107.2 |
| StD | 17.14 | 17.06 | 17.09 |
| Median | 106.0 | 105.0 | 109.5 |
| 25%/75% quantile | 94.0/117.0 | 92.0/116.0 | 97.0/119.8 |
| Min/Max | 34/140 | 34/140 | 42/140 |

| Table 55.1 - CFI (overall and by sex) | | | |
| --- | --- | --- | --- |
| MAS | | | |
|  | Total (N = 1989) | Female (N = 1515) | Male (N = 474) |
| n | 1989 | 1515 | 474 |
| Mean | 104.5 | 103.6 | 107.2 |
| StD | 17.17 | 17.11 | 17.09 |
| Median | 106.0 | 105.0 | 109.0 |
| 25%/75% quantile | 94.0/117.0 | 93.0/116.0 | 97.0/119.8 |
| Min/Max | 34/140 | 34/140 | 42/140 |

| Table 55.2 - CFI (by age) | | | |
| --- | --- | --- | --- |
| ES | | | |
|  | Age 18-30 (N = 892) | Age 31-65 (N = 1662) | Age >65 (N = 98) |
| n | 700 | 1302 | 79 |
| Mean | 101.6 | 106.0 | 101.0 |
| StD | 17.29 | 16.99 | 14.87 |
| Median | 102.0 | 108.0 | 101.0 |
| 25%/75% quantile | 91.0/113.0 | 95.0/118.0 | 90.5/110.0 |
| Min/Max | 43/140 | 34/140 | 66/140 |

| Table 55.2 - CFI (by age) | | | |
| --- | --- | --- | --- |
| MAS | | | |
|  | Age 18-30 (N = 666) | Age 31-65 (N = 1258) | Age >65 (N = 65) |
| n | 666 | 1258 | 65 |
| Mean | 101.5 | 106.2 | 101.7 |
| StD | 17.37 | 16.97 | 14.62 |
| Median | 102.0 | 108.0 | 100.0 |
| 25%/75% quantile | 91.0/113.0 | 95.0/118.0 | 92.0/110.0 |
| Min/Max | 43/140 | 34/140 | 68/140 |

| Table 55.3 - CFI (by ISI group) | | | | | |
| --- | --- | --- | --- | --- | --- |
| ES | | | | | |
|  | Absence of insomnia (N = 1247) | Subthreshold insomnia (N = 806) | Moderate insomnia (N = 382) | Severe insomnia (N = 70) | Unknown (N = 147) |
| n | 1023 | 664 | 318 | 57 | 19 |
| Mean | 107.4 | 102.7 | 99.6 | 95.9 | 100.4 |
| StD | 15.82 | 17.28 | 18.03 | 22.41 | 13.62 |
| Median | 109.0 | 104.0 | 100.0 | 97.0 | 102.0 |
| 25%/75% quantile | 98.0/119.0 | 91.8/116.0 | 88.0/112.0 | 83.0/115.0 | 87.5/109.5 |
| Min/Max | 45/140 | 41/139 | 47/140 | 34/132 | 78/131 |

| Table 55.3 - CFI (by ISI group) | | | | |
| --- | --- | --- | --- | --- |
| MAS | | | | |
|  | Absence of insomnia (N = 977) | Subthreshold insomnia (N = 642) | Moderate insomnia (N = 314) | Severe insomnia (N = 56) |
| n | 977 | 642 | 314 | 56 |
| Mean | 107.6 | 102.9 | 99.5 | 95.4 |
| StD | 15.70 | 17.34 | 18.08 | 22.38 |
| Median | 109.0 | 104.0 | 100.0 | 96.0 |
| 25%/75% quantile | 98.0/119.0 | 92.0/116.0 | 88.0/112.0 | 82.8/112.0 |
| Min/Max | 45/140 | 41/139 | 47/140 | 34/132 |
