# Supplementary material for: Insomnia in the Italian Population During Covid-19 Outbreak: A Snapshot on One Major Risk Factor for Depression and Anxiety
Source: Front Psychiatry. 2020 Dec 15;11:579107. doi: 10.3389/fpsyt.2020.579107 (PMC7769843; doi:10.3389/fpsyt.2020.579107)
Supplement: Supplementary file 2 [file Data_Sheet_2.docx]

**Document S2** Multivariable linear regression: Variables preparation and results

*Variables preparation:*

- Sex (categorical: Male vs Female);
- Age (continuous);
- Past mental disorder (categorical: No vs Yes: Insomnia only, Any mental disorder (± insomnia));
- Current mental disorder (categorical: No vs Yes: Insomnia only; Current Mental Disorder ± insomnia);
- ERQ-ES (continuous): emotion regulation expressive suppression strategy;
- ERQ-CR (continuous): emotion regulation cognitive reappraisal strategy;
- CFI (continuous): cognitive flexibility;
- PSS (continuous variable): perceived stress;
- SHI (continuous variable): sleep hygiene behaviours;
- DBAS-16 (continuous variable): dysfunctional beliefs about sleep;
- Exercise behaviour (categorical: Never: Answered “No” to both questions about exercise before and now vs Continued: Answered “Yes” to both questions about exercise before and now vs Quit: Answered “Yes” to exercise before, and “No” to exercise now vs Started: Answered “No” to exercise before, and “Yes” to exercise now);
- Eating behaviour (categorical: No: Answered “no changes from before in eating habits” vs Less healthy: Answered “yes, I eat more” OR “yes, I eat less healthy” vs Less eating: Answered “yes, I eat less” OR “yes, I eat more healthy”);
- Drinking behaviour (categorical: No: Answered “no changes from before in alcohol habits” vs Less healthy: Answered “yes I drink more liquor” OR “yes I drink more alcohol” vs Less alcohol: Answered “yes I drink less alcohol”);
- More use of devices (categorical: Yes vs No);
- Region (categorical: North vs Other);
- Health related work (categorical: Yes vs No);
- Tested positive (categorical: Yes vs No);
- Mourning (categorical: Yes vs No);
- Number of household members (categorical: 1: living alone vs 2: living with one other person vs 3-4: living with 2 or 3 other persons vs ≥ 5: living with 4 or more persons);
- Circadian preference (MEQ: categorical: morning type vs intermediate type vs evening type);
- HADS-A (continuous variable): anxiety symptoms
- HADS-D (continuous variable): depression symptoms

*Multivariable linear regression*

|  | | | |  |
| --- | --- | --- | --- | --- |
|  | | | |  |
|  | **b** | **95% CI** | **p-value** | **Adj. p-value** |
| **Sex** |  |  |  |  |
| **Male** | **Reference** |  |  |  |
| **Female** | **-0.4127** | **[-0.9247, 0.0993]** | **0.1141** | **1.000** |
| **Age** | **0.0048** | **[-0.0136, 0.0231]** | **0.6098** | **1.000** |
| **Past mental disorder** |  |  |  |  |
| **No mental disorder** | **Reference** |  |  |  |
| **Insomnia only** | **0.4511** | **[-1.4523, 2.3546]** | **0.6421** | **1.000** |
| **Any mental disorder (± insomnia)** | **-0.4820** | **[-1.0049, 0.0410]** | **0.0708** | **1.000** |
| **Current mental disorder** |  |  |  |  |
| **No mental disorder** | **Reference** |  |  |  |
| **Insomnia only** | **2.7796** | **[ 0.9760, 4.5833]** | **0.0025** | **0.069** |
| **Any mental disorder (± insomnia)** | **2.5115** | **[ 1.8277, 3.1952]** | **<0.001** | **<0.001** |
| **ERQ-ES** | **0.0111** | **[-0.0288, 0.0509]** | **0.5856** | **1.000** |
| **ERQ-CR** | **-0.0131** | **[-0.0461, 0.0199]** | **0.4368** | **1.000** |
| **CFI** | **0.0137** | **[-0.0009, 0.0283]** | **0.0657** | **1.000** |
| **PSS** | **0.0367** | **[-0.0073, 0.0808]** | **0.1022** | **1.000** |
| **SHI** | **0.1141** | **[ 0.0799, 0.1484]** | **<0.001** | **<0.001** |
| **DBAS-16** | **0.0985** | **[ 0.0851, 0.1119]** | **<0.001** | **<0.001** |
| **Exercise behaviour** |  |  |  |  |
| **Never** | **Reference** |  |  |  |
| **Continued** | **0.2190** | **[-0.2543, 0.6922]** | **0.3642** | **1.000** |
| **Started** | **-0.2903** | **[-1.5430, 0.9624]** | **0.6495** | **1.000** |
| **Quit** | **-0.0459** | **[-0.6353, 0.5436]** | **0.8787** | **1.000** |
| **Eating behaviour** |  |  |  |  |
| **No, not changed** | **Reference** |  |  |  |
| **Yes, less healthy** | **0.1905** | **[-0.3033, 0.6843]** | **0.4494** | **1.000** |
| **Yes, more healthy** | **0.0642** | **[-0.5269, 0.6552]** | **0.8314** | **1.000** |
| **Drinking behaviour** |  |  |  |  |
| **No, not changed** | **Reference** |  |  |  |
| **Yes, more alcohol** | **-0.0893** | **[-0.6964, 0.5178]** | **0.7730** | **1.000** |
| **Yes, less alcohol** | **-0.1731** | **[-0.7650, 0.4188]** | **0.5664** | **1.000** |
| **More use of devices** |  |  |  |  |
| **No** | **Reference** |  |  |  |
| **Yes** | **0.2420** | **[-0.3063, 0.7902]** | **0.3869** | **1.000** |
| **Region** |  |  |  |  |
| **Other** | **Reference** |  |  |  |
| **North** | **0.1647** | **[-0.2552, 0.5846]** | **0.4418** | **1.000** |
| **Health-related work** |  |  |  |  |
| **No** | **Reference** |  |  |  |
| **Yes** | **-0.1223** | **[-1.0555, 0.8109]** | **0.7972** | **1.000** |
| **Tested positive** |  |  |  |  |
| **No** | **Reference** |  |  |  |
| **Yes** | **-0.9287** | **[-2.8029, 0.9454]** | **0.3312** | **1.000** |
| **Mourning** |  |  |  |  |
| **No** | **Reference** |  |  |  |
| **Yes** | **0.3145** | **[-0.4927, 1.1217]** | **0.4449** | **1.000** |
| **Number of household members** |  |  |  |  |
| **1** | **Reference** |  |  |  |
| **2** | **-0.3707** | **[-1.0356, 0.2942]** | **0.2743** | **1.000** |
| **3-4** | **-0.0101** | **[-0.6580, 0.6378]** | **0.9757** | **1.000** |
| **≥5** | **0.3645** | **[-0.6351, 1.3641]** | **0.4747** | **1.000** |
| **Circadian preference** |  |  |  |  |
| **Intermediate type** | **Reference** |  |  |  |
| **Morning type** | **0.0774** | **[-0.4542, 0.6090]** | **0.7752** | **1.000** |
| **Evening type** | **0.5986** | **[-0.0277, 1.2248]** | **0.0610** | **1.000** |
| **HADS-A** | **0.3373** | **[ 0.2532, 0.4215]** | **<0.001** | **<0.001** |
| **HADS-D** | **0.2455** | **[ 0.1651, 0.3259]** | **<0.001** | **<0.001** |
